# Rapid bursts of *androgen-binding protein (Abp) *gene duplication occurred independently in diverse mammals

**DOI:** 10.1186/1471-2148-8-46

**Published:** 2008-02-12

**Authors:** Christina M Laukaitis, Andreas Heger, Tyler D Blakley, Pavel Munclinger, Chris P Ponting, Robert C Karn

**Affiliations:** 1 Department of Medical Genetics, University of Washington, Seattle, USA and Division of Human Biology, Fred Hutchinson Cancer Research Center, Seattle, USA; 2 MRC Functional Genetics Unit, Department of Physiology, Anatomy and Genetics, University of Oxford, Oxford, UK; 3 Department of Biological Sciences, Butler University, Indianapolis, USA; 4 Department of Zoology, Faculty of Sciences, Charles University in Prague, Praha, Czech Republic; 5 MRC Functional Genetics Unit, Department of Physiology, Anatomy and Genetics, University of Oxford, Oxford, UK; 6 Department of Biological Sciences, Butler University, Indianapolis, USA and Department of Genome Sciences, University of Washington, Seattle, USA

## Abstract

**Background:**

The draft mouse (*Mus musculus*) genome sequence revealed an unexpected proliferation of gene duplicates encoding a family of secretoglobin proteins including the androgen-binding protein (ABP) α, β and γ subunits. Further investigation of 14 α-like (*Abpa*) and 13 β- or γ-like (*Abpbg*) undisrupted gene sequences revealed a rich diversity of developmental stage-, sex- and tissue-specific expression. Despite these studies, our understanding of the evolution of this gene family remains incomplete. Questions arise from imperfections in the initial mouse genome assembly and a dearth of information about the gene family structure in other rodents and mammals.

**Results:**

Here, we interrogate the latest 'finished' mouse (*Mus musculus*) genome sequence assembly to show that the *Abp *gene repertoire is, in fact, twice as large as reported previously, with 30 *Abpa *and 34 *Abpbg *genes and pseudogenes. All of these have arisen since the last common ancestor with rat (*Rattus norvegicus*). We then demonstrate, by sequencing homologs from species within the *Mus *genus, that this burst of gene duplication occurred very recently, within the past seven million years. Finally, we survey *Abp *orthologs in genomes from across the mammalian clade and show that bursts of *Abp *gene duplications are not specific to the murid rodents; they also occurred recently in the lagomorph (rabbit, *Oryctolagus cuniculus*) and ruminant (cattle, *Bos taurus*) lineages, although not in other mammalian taxa.

**Conclusion:**

We conclude that *Abp *genes have undergone repeated bursts of gene duplication and adaptive sequence diversification driven by these genes' participation in chemosensation and/or sexual identification.

## Background

Approximately 90% of genes in the dog (*Canis familiaris*), mouse (*Mus musculus*), and human (*Homo sapiens*) genomes have been preserved without duplication, disruption or deletion, since their last common ancestors [1,2]. The remaining ~10% of genes, representing the volatile portion of the mammalian gene complement, in general possess functions that are very different from those of the conserved gene set: they are substantially enriched in functions contributing to chemosensation, reproduction, immunity, host defense and toxin degradation [3]. Moreover, gene families, such as those containing olfactory or vomeronasal receptors, immunoglobulin domain-containing proteins, or cytochromes P450 that are expanded in one lineage are often expanded in another. This likely reflects similar evolutionary responses to similar environmental challenges.

Some among us contributed to initial analyses of the draft mouse genome assembly [2] by identifying mouse-specific gene duplications that remained unduplicated in the human genome. We argued that such genes contribute disproportionately to biology that is specific to the rodent lineage [3]. One of these mouse-specific clusters includes homologs of the α-subunit of the androgen-binding protein (ABP) which was of particular interest owing to evidence that ABP mediates sexual selection in mice [4,5]. Others among us have investigated the biochemical, genetic, molecular and physiological functions, and evolution of ABP molecules since the 1980s. These investigations revealed that the ABP heterodimer consists of an α-subunit (encoded by the *Abpa *gene), disulfide-bridged to either a β- or a γ-subunit (encoded by *Abpb *or *Abpg*; [6,7]), and that *Abp *genes evolved rapidly, potentially because of positive selection [7-11]. In common with all other members of the secretoglobin family to which they belong, ABP subunits have not unequivocally been assigned molecular functions [12-15].

Upon combining our research efforts, we first performed a more detailed comparative analysis of *Abp *gene orthologs in the mouse, rat (*Rattus norvegicus*), chimpanzee (*Pan troglodytes*) and human draft genome assemblies [8]. These analyses established that *Abp *sequences, present within human and chimpanzee regions of conserved synteny, have acquired disruptions to their reading frames and these are highly likely to be pseudogenes. Mouse *Abp *genes appear to have been subject to positive selection with coding sequences acquiring greater numbers of nucleotide substitutions than their introns. Surprisingly, mouse *Abpa *and *Abpbg *genes were found all to be monophyletic with respect to families of orthologs from either rat or wood mouse (*Apodemus*). This is consistent with multiple duplications of *Abpa *and *Abpbg *genes occurring independently in each of the mouse, rat and *Apodemus *lineages, and with the genome of the last ancestor common to rodents and primates containing only single versions of *Abpa *and *Abpbg *genes. It is also consistent with the presence, in the cat genome, of single adjacent *Abpa *and *Abpbg *orthologs, whose protein products form a heterodimer termed Fel dI [16]. In a subsequent study, we investigated the spatiotemporal expression of seventeen *Abp *genes, and concluded that differential expression patterns of mouse *Abp *genes reflect an animal's gender and sexual maturity status [17].

The mouse genome assembly is substantially improved: it is now essentially complete and is of high accuracy (Mouse Genome Sequence Finishing Consortium, submitted). The draft mouse genome previously analyzed (NCBI build 30, mm3; [8] contained four large (>5 kb) gaps, into which we now realize fall many hitherto unrecognized *Abpa *and *Abpbg *paralogues. We were interested in whether these paralogues arose more rapidly than the genome-wide average, for primates and rodents, of 1 duplicate gene fixed per hundred million years [18].

**Figure 1:**
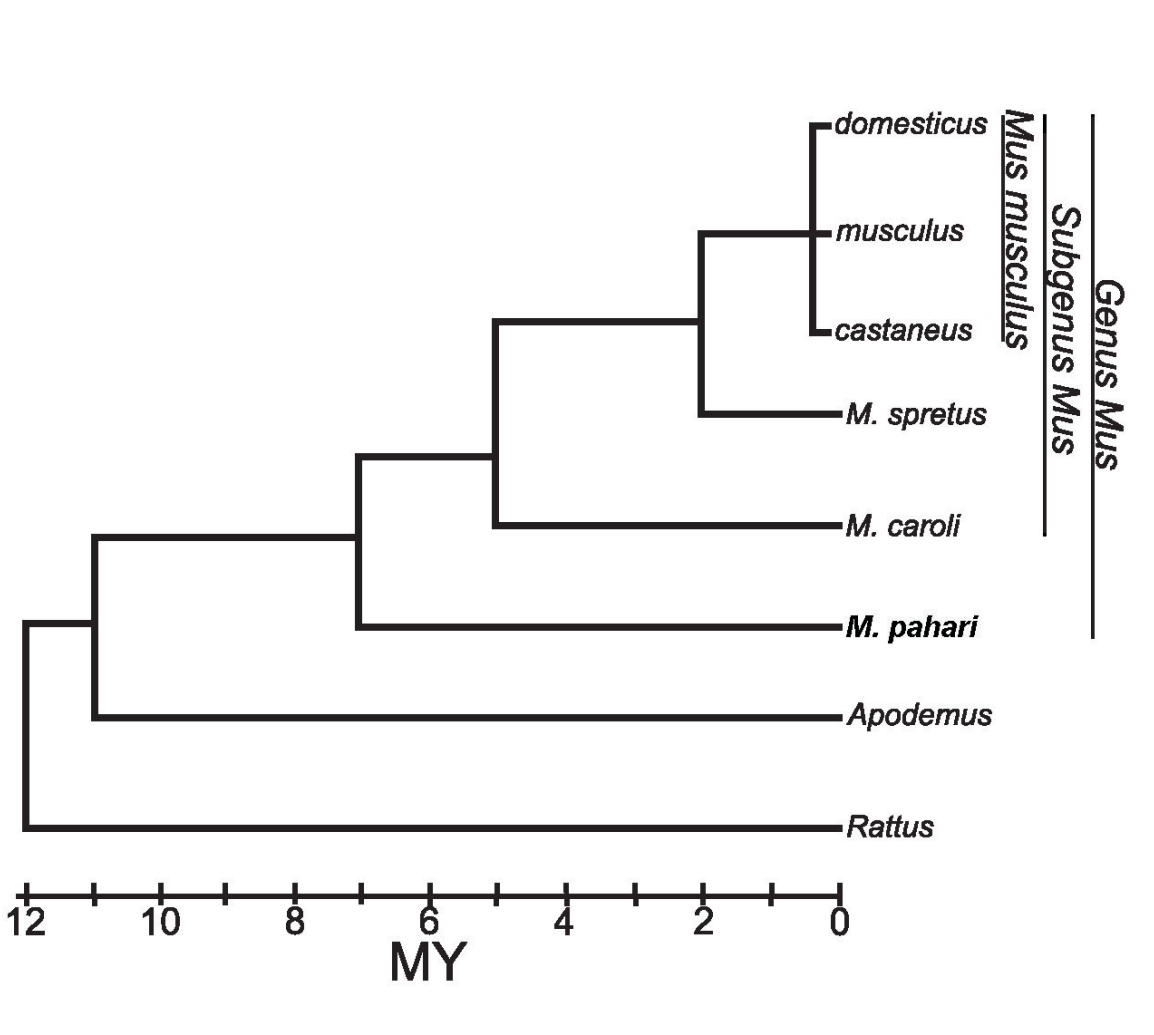
A canonical phylogeny of murid rodents (adapted from [20]).

In the absence of genome sequences from more closely related species, we sequenced *Abpa *homologs from three *Mus musculus *subspecies (*M. m. domesticus*, *M. m. musculus *and *M. m. castaneus*) and three other species within the genus *Mus *(*M. spretus*, *M. caroli*, and *M. pahari *– see Fig. 1 for a phylogeny). *M. musculus *and *M. spretus *represent the Palearctic clade, *M. caroli *is an Asian species serving as an outgroup to the Palearctic species, whereas *Mus pahari *(a.k.a. *Coelomys*; [19]) serves as an outgroup to the subgenus *Mus*; these species all diverged from a common ancestor more than seven million years ago [20]. Our finding of single *M. pahari *orthologs of multiple *Mus musculus Abpa *genes thus supports the rapid duplication of these *Mus *genes within the last seven million years.

As the common ancestor of murid rodents appeared to contain only a single pair of *Abp *genes and because gene duplication events are relatively rare, we predict that single *Abpa*-like and *Abpbg*-like genes will also be present in the genomes of most other eutherian mammals, and of metatherians, such as the opossum (*Monodelphis domestica*). The recent availability of draft assemblies of many species' genomes [21] provides an opportunity to investigate this prediction. While our methodology provides slight underestimates of the numbers of *Abpa *and *Abpbg *paralogues and precludes definitive assignment of predicted sequences as genes or pseudogenes, here we report identification of the expected single pair of *Abpa *and *Abpbg *genes in genome assemblies for multiple diverse mammals. Interestingly, we identified considerably more such paralogues in two mammals, rabbit and cattle, hinting that *Abp *genes have been the substrate for frequent duplication in lineages beyond the murid rodents.

## Results

### Rodent gene predictions and nomenclature

**Figure 2:**
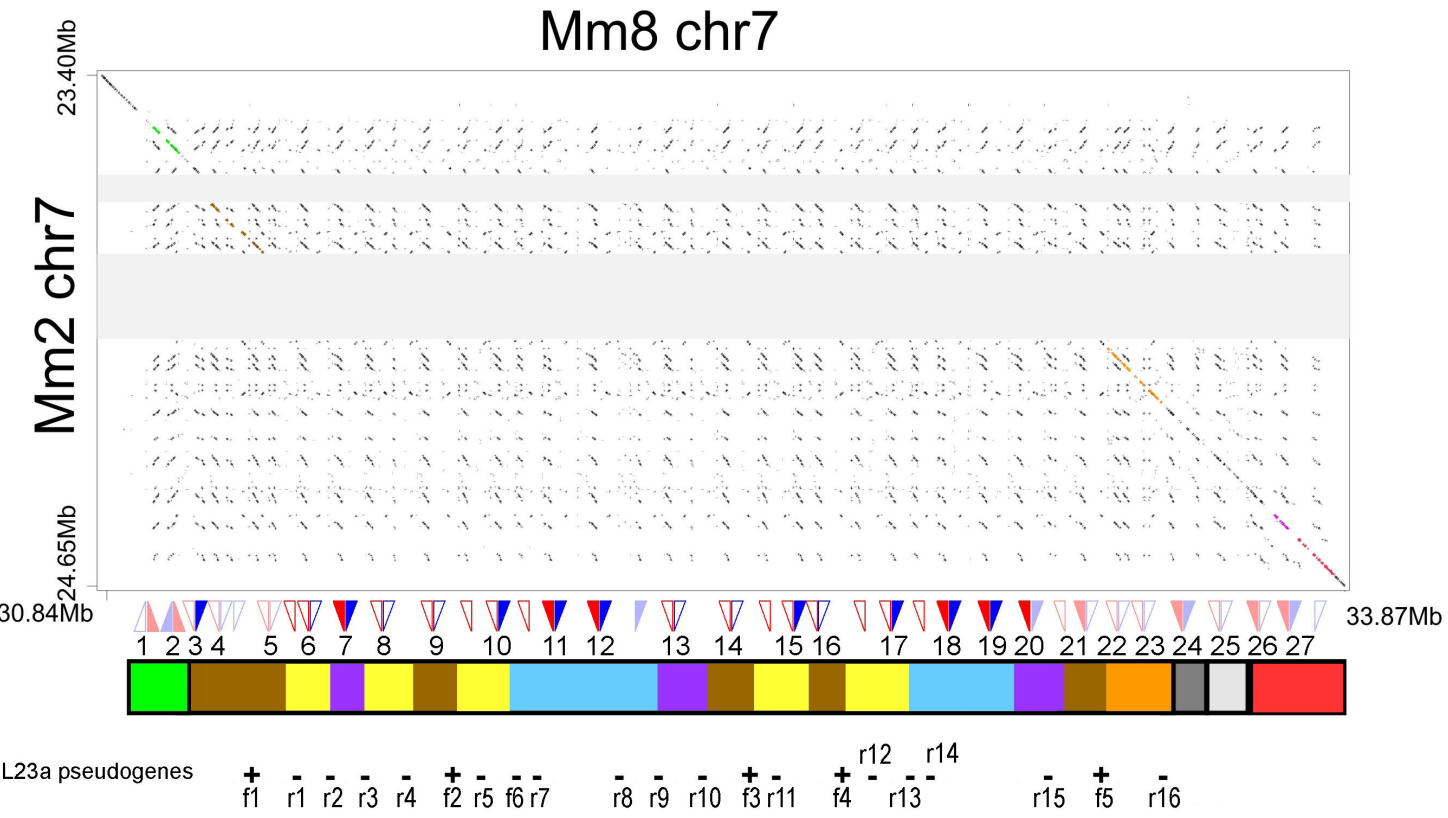
Pipmaker [61] dot plot of Abp-containing regions for the mm2 and mm8 assemblies of the mouse (C57BL6/J) genome sequence. Transposable element sequences have been masked in order to emphasize this region's coding sequences. Two grey shaded regions represent gaps in the earlier mm2 assembly. The second of these can be now seen, in mm8, to harbour a large number of *Abp *genes that were absent from mm2. Predicted genes and pseudogenes are indicated as filled and unfilled triangles, respectively. *Abpa *paralogues are shown in blue and *Abpbg *paralogues are shown in red. The original paralogues described by Emes et al. [8] are faded while the new paralogues we describe here are in bright colors. Ribosomal protein L23a pseudogenes on the forward and reverse strands are shown as '+' and '-' symbols, respectively, and are numbered at bottom. The five *Mus musculus Abpa *clades, each predicted to have originated from a single gene in the last common ancestor with *Mus pahari*, are indicated by rectangles out-lined in black on the bar beneath the paralogue symbols. Each of the five rectangles is annotated with the *Abpa*-*Abpbg *gene pairs (namely 1–2, 3–23, 24, 25, and 26–27) that arose from these single common ancestors and, where appropriate, the identity line in the pip plot is also shaded the appropriate color. More recent, *Mus *lineage-specific, duplications are represented by smaller colored rectangles embedded within the outlined rectangles.

We revisited the *Abp *region of the mouse (C57BL/6J strain) genome sequence considering that a recent assembly (mm8; February 2006) might contain genes that had been hidden within the gaps of the 2002 initial draft assembly (mm2). We report that this region is now free of sequence gaps. By comparing new (mm8) and old (mm2) genome assemblies we identified 41 *Abp *genes in a large (~1.8 Mb) contiguous segment that had presented difficulties when originally assembled (Figure 2). The mouse *Abp *gene region is now seen to be 3 Mb in size, approximately twice as large as previously estimated, and 6-fold larger than estimated for the orthologous region in rat [8].

*Abp *genes were originally described as *Abpa *(alpha subunit; A), *Abpb *(beta subunit; B) and *Abpg *(gamma subunit; G), based on the identification of the three ABP subunits in two different dimeric combinations: AB and AG [6]. The three genes were mapped on the proximal end of mouse chromosome 7 in a study that also demonstrated that *Abpb *and *Abpg *are closely related, both in exon/intron structure and in sequence [7]. When it became apparent that multiple copies of *Abpa *and of *Abpb/Abpg *exist in the genomes of mouse and rat, with 5'-5' orientations of pairs of *Abpa *and *Abpbg*, the nomenclature was modified to represent *Abpb *and *Abpg *as *Abpbg *genes [8].

The mouse *Abp *region contains a total of 30 *Abpa *and 34 *Abpbg *genes or pseudogenes (see Additional files 1 &2 for cDNA sequences and gene locations), over twice the numbers we reported previously [8]. In the report that follows, we distinguished the *Abpa *paralogues obtained from the mouse genome from those we derived from wild-derived rodent taxa by designating them "B6" (an abbreviation of C57BL/6, the source strain for the mouse genome project). New B6 paralogues reported here are designated in bold typeface. Twelve of the 30 *Abpa *genes and 19 of the 34 *Abpbg *genes have missense or nonsense mutations in an exon, while four *Abpa *and three *Abpbg *paralogues have non-canonical splice sites (all marked as pseudogenes on Figure 2; details in Additional file 2). We recognize that pseudogenes that possess full-length open reading frames are mis-assigned as functional genes by this method. It is also possible that one or more of those we have designated pseudogenes could be expressed. Of note is the paralogue B6_*bg2 *(previously numbered *Abpbg*2 [8]), which has a deletion eliminating a conserved stop codon that allows the protein sequence to be extended by two extra amino-acid residues before ending in a non-conserved stop codon. We have assigned this paralogue gene status because we have identified transcripts by RT-PCR for this gene in numerous tissues [17] and have found at least 10 *Mus musculus *ESTs for this slightly longer version in the *Mus musculus *EST database (CK616889 and others). Others working on Swiss-Webster mice reported a different change at the 3' end of this paralogue (AY370634), supporting the possibility of strain-specific polymorphisms within this gene.

Consistent with previous observations, the majority of the newly-predicted genes are located in *Abpa*-*Abpbg *pairs, and are likely to be transcribed in opposite, head-to-head (i.e. 5'-5') arrangements. Our original gene sequence and pair assignments are likewise essentially unchanged from our initial report [8]. We number the *Abpa *and *Abpbg *paralogues by their sequential occurrence in *Abpa*-*Abpbg *gene pairs along the chromosome (i.e., B6_*Abpa1*-B6_*Abpbg*1, B6_*a2*-B6_*bg2*, etc.; Figure 2) despite this resulting in the reassignment of some gene names (see Additional file 2 for current and previous designations).

### The Abp gene cluster in other rodent taxa

The most recent rat genome assembly (rn4; November 2004) contains only three *Abpa*-*Abpbg *gene pairs in a region spanning ~0.2 Mb of chromosome 1 (RNO1), which is ten times fewer than the *Abp *genes distributed over ~3 Mb of the syntenic region of mouse chromosome 7 (MMU7). Originally, we reported that one of the six rat paralogues was a pseudogene [8]. However, we have since discovered a cDNA with a single base sequence change in that gene which eliminates a frameshift mutation resulting from an additional A nucleotide in a run of A's (Laukaitis and Karn, unpublished). Although this region of the rat genome assembly contains many gaps (including 5 contig gaps) there is no evidence, as there was for previous mouse assemblies, from unplaced genomic and cDNA sequences for additional *Abp *gene paralogues.

**Figure 3:**
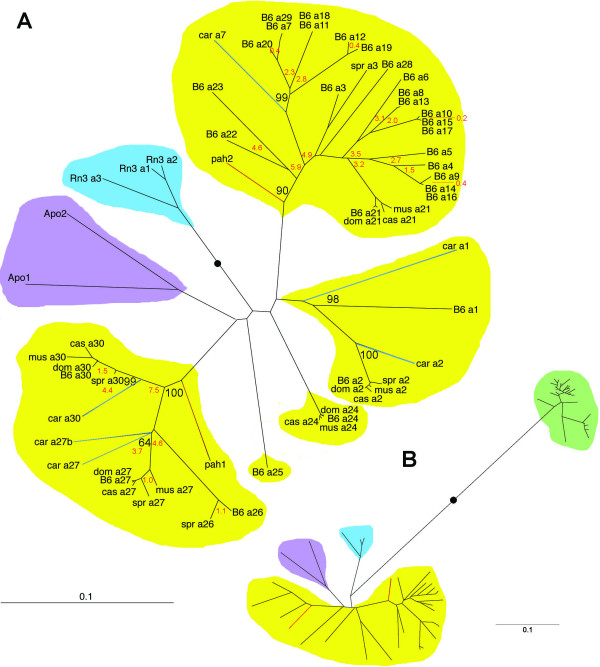
Panel A: NJ phylogeny of intron 2 from rodent *Abpa *genes. Values from a superimposable bootstrap tree were used to annotate a phylogenetic tree calculated with neighbor joining methodology. The 30 *Abpa *genes found in mm8 are shown as B6_*a1*-*a30*. Corresponding genes found in subgenus *Mus *taxa are abbreviated: dom (*M. m. domesticus*), *mus *(*M. m. musculus*), cas (*M. m. castaneus*), spr (*M. spretus*) and car (*M. caroli*). *Abpa *genes found in other murid taxa are abbreviated: pah (*M. pahari*), Apo (*Apodemus*) and Rn3 (2004 *Rattus *genome release). Subgenus *Mus Abpa *clades are shaded in yellow. In the case of two of these clades, an *M. pahari *paralogue appears as an outgroup (branch shaded in red). *M. caroli *paralogues serve as outgroups to *M. musculus *clades (branches shaded in blue). The *Apodemus *clade is shaded purple and the rat clade blue. The bootstrap values for all internal nodes except two exceeded 60%; key bootstrap values are shown in black typeface. Divergence times in millions of years (MY) are shown in red typeface. Panel B: NJ phylogeny of intron 2 from rabbit and rodent *Abpa *genes. The rabbit clade is shown with a green background; the rodent clades are shaded as in Panel A. In both panels, the black dots represent the probable roots of these phylogenies, the locations of which are supported by the locations of rat (Panel A) and rabbit (Panel B) genes which serve as outgroups.

A phylogenetic tree was constructed using the second introns (of length ~800 bp) available from the mouse (genome: "B6"), rat (genome), *Apodemus*, *M. m. domesticus*, *M. m. musculus*, *M. m. castaneus*, *M. spretus*, *M. caroli *and *M. pahari *(Figure 3; see Additional file 3 for intron sequences). As noted in Materials and Methods, this is the first report of intron sequences from *Apodemus*. The *Abpa *genes in *Mus musculus, Apodemus *and *Rattus *cluster separately into three monophyletic clades, indicating that the mouse, *Apodemus *and rat *Abp *gene complements arose by duplication independently from single *Abpa *or *Abpbg *genes in the last common ancestor; this is consistent with our previous results [8]. However, whether these rodent *Abp *gene duplications were relatively contemporaneous, or whether they were more uniformly distributed over time, remained unclear.

To deduce the pace of gene duplication in the *Mus musculus *lineage, we used PCR to obtain *Abpa *gene sequences in closely-related rodent species (see lineage in Figure 1). Thereafter, we used Southern blotting to interrogate genomic DNA from different rodent taxa using *Abpa *probes from *M. m. domesticus *and *M. pahari *paralogues. We chose to focus on *Abpa *genes, because extensive repeats of single bases in *Abpbg *introns ([7] and Karn, unpublished) make it difficult to amplify and sequence. Since most *Abpa *genes are paired with single *Abpbg *genes, our results are also relevant to *Abpbg *genes. The effectiveness of each of these methods necessarily depends on the evolutionary divergence between *Abpa *probe sequences and the other rodent taxa being studied. Such approaches resulted in the identification and partial sequencing of two *Abpa *genes in *M. pahari*, six in *M. caroli*, and five each in *M. spretus*, *M. m. castaneus*, *M. m. musculus*, and *M. m. domesticus*.

By manually reconciling the known species phylogeny (Figure 1) with a phylogeny of these genes and pseudogenes (Figure 3), we deduce that many of the 30 mouse genome *Abpa *genes and pseudogenes arose by duplications within the *Mus *subgenus clade. The basal positions of each of the *Rattus*, *Apodemus *or *M. pahari *clades indicate that the last common ancestor of *Mus*, *Apodemus*, and *Rattus *contained only one *Abpa *gene. The last common ancestor of the *Mus *subgenus with *M. pahari *contained at minimum two and at maximum five *Abpa *genes since the *Abpa *genes from the *Mus *subgenus (*M. m. musculus*, *M. m. domesticus*, *M. m. castaneus*, *M. spretus*, and *M. caroli*) form five well-separated clades, and each of the two *M. pahari Abpa *genes serves as an outgroup to one of these clades.

The six *Abpa *genes from *M. caroli*, the most distant species of those we investigated in the subgenus *Mus*, are all outgroups to the other *Abpa *(pseudo)genes from the subgenus. For all but two of these genes, the branching order is consistent with the species phylogeny without lineage-specific duplication events. For *M. caroli Abpa7*, the branching order supports the origin of eight mouse genome in-paralogues (**B6_*a7*, *a11*, *a12*, *a13*, *a18*, *a19*, *a20 ***and *a2*9; these are all newly reported here except *B6_a2*9 which was previously referred to as *Abpa13 *[8]) after the splitting of the *M. caroli *and *M. musculus *lineages (Figure 3). We calculate that these duplication events occurred between 400,000 and 2.8 million years ago, although the low divergences for introns from some of these paralogues suggest even more recent duplication events. We considered, and eventually discarded, the possibility that gene conversion has caused the low divergence of these intron sequences. For the *B6_a27 *clade (previously reported as *Abpa11*[8] and, originally, *Abpa *[6]), two *M. caroli *paralogues cluster as outgroups, suggesting a *M. caroli*-specific duplication event. B6 duplications within this clade occurred between 1 and 7 million years ago.

### Southern blotting

**Figure 4:**
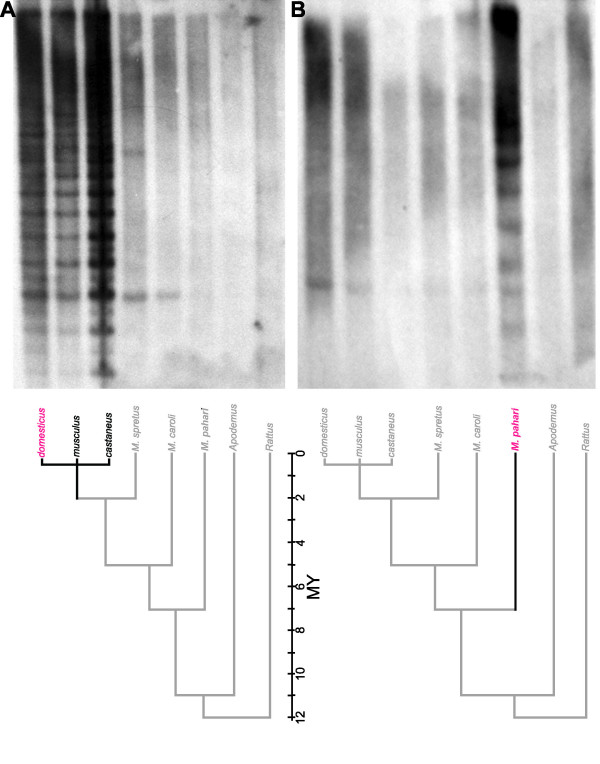
Blots of DNA electrophoresis gels (Southern transfers). Genomic DNA from various rodent taxa were digested with EcoRI and separated on 1% agarose gels. Panels A and B show equivalent transfers probed separately with a *M. m. domesticus Abpa *probe (Panel A; *M. musculus *subspecies highlighted) and a *M. pahari *probe (Panel B; *M. pahari *highlighted). A canonical phylogeny of murid rodents (adapted from [20]) is shown below Panel A.

The patterns produced by probing Southern transfers of DNA from the three subspecies of *M. musculus *with a variety of *Abpa*-derived probes are essentially indistinguishable, independent of probe or subspecies (Figure 4). This consistency suggests that much of the extensive *Abp *gene expansion seen in the C57BL/6J genome ([8] and this paper) was present in the common ancestor of these subspecies approximately 0.5 MYA. Pattern intensity was reduced dramatically in more distant taxa of the subgenus *Mus*, especially at higher stringency levels, reflecting sequence divergence. Bands in three taxa outside the subgenus *Mus *(*M. pahari*, the outgroup to the subgenus; *Apodemus*, the outgroup to the genus *Mus*; and *Rattus*, the most distantly related, Figure 4A), were faint at low stringency and disappeared altogether as stringency was increased.

The two *M. pahari *genes we identified allowed us to develop Southern blotting probes to further investigate the relationship of *M. pahari *paralogues to those discovered in the subgenus *Mus*. Two separate experiments, using probes developed from each of the *M. pahari *paralogues, produced nearly identical patterns of eight bands in the *M. pahari *lane and few or no bands in other taxa (Figure 4B). Conversely, probing *M. pahari *genomic DNA with the *M. m. domesticus *probes revealed only one band, again reflecting sequence divergence between genes from the taxa. The *Apodemus *and *Rattus *lanes contained no distinguishable bands at higher stringency, as was the case when probed with *M. m. domesticus *probes.

### Abp genes found in the genomes of other mammals

**Figure 5:**
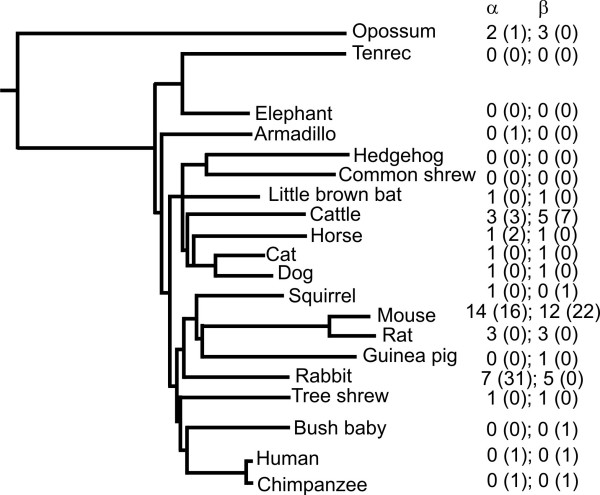
Predicted minimum gene number per species for mammals whose genome sequences are currently available. Genes are indicated below their α or β subunit heading. Pseudogenes are shown in parentheses. The phylogeny is modified from [22]. Species names and data sources are provided in the Materials and Methods. Results for macaque and platypus are not shown owing to the absence of evidence for *Abp *genes in their current genome assemblies.

We previously predicted that the last common ancestors of mouse and rat, and of mouse and human, possessed only a single pair of *Abpa*-*Abpbg *genes [8]. We sought to test this prediction by identifying *Abp *genes in the available assembled genome sequences of seventeen additional mammalian species that sample divergent eutherian lineages, one metatherian, and one monotreme species [22]. *Abpa *or *Abpbg *orthologs were identified for 12 of these species (Figure 5; see Additional files 1 &2 for cDNA sequences and contig assignments). Supporting our prediction, no more than a single *Abpa *or *Abpbg *gene was found for ground squirrel and guinea pig, both of which are rodents. Similarly, single gene pairs, at most, were found for four species (little brown bat, horse, dog, and cat) whose lineages arose prior to the last common ancestor of primates and rodents. We conclude the earliest eutherian possessed only a single pair of *Abpa *and *Abpbg *genes.

The last common ancestor of the eutherian and metatherian lineages 180 MYA possessed both *Abpa *and *Abpbg *genes. Three *Abpa *and three *Abpbg *subfamily members are evident in the metatherian *Monodelphis *genome sequence although many of these, owing to their divergence, were unable to be predicted in their entireties. Two *Monodelphis Abpa *genes appear to have arisen from duplication within the metatherian lineage, as evidenced by their relatively low divergence at synonymous sites (K_S _= 0.06). *Abp *genes, on the other hand, were not detected in the draft platypus genome, but we cannot exclude that sequence divergence disfavours their prediction using the methods we employed. Alternatively, *Abp *genes may simply lie within the ~10% of its genome that is absent from the current assembly.

By way of contrast, the genomes of two further species each contain independent expansions of *Abp *genes. The rabbit genome assembly contains 43 *Abpa *or *Abpbg *sequences, whereas the cattle genome assembly contains 18 such homologs. *Abpa*-*Abpbg *gene pairs are observed in head-to-head (5'-5') arrangements on 7 and 4 occasions within cattle and rabbit contig sequences. Rabbit gene paralogues are more closely related (maximum K_S _= 0.16) than they are to closely-related orthologs in other species (guinea pig or squirrel minimum K_S _= 0.78 and K_S _= 0.97, respectively). Similarly, cattle paralogues are more closely related (maximum K_S _= 0.49) to each other, than they are to their dog or cat orthologs (minimum K_S _= 0.74 and 0.51, respectively). We infer, therefore, that expansions of *Abp *genes have occurred independently in the cattle and rabbit lineages.

In the macaque genome assembly, we were able to identify neither an *Abpbg *exon 1 (which is all that is discernible in the human and chimpanzee genome assemblies [8]), nor an *Abpa *gene. Although it remains possible that *Abpa *and *Abpbg *genes or pseudogenes fall into gaps that are present in the syntenic region of the current macaque genome assembly, evidence for these homologues was also not forthcoming from searches of the macaque trace sequence reads. We are yet unable, therefore, to delineate definitively the time-points in the primate lineage at which *Abpa *and *Abpbg *genes acquired deleterious mutations.

We determined that the numbers of *Abp *genes present in the 2-fold coverage genome assemblies represented reliable estimates and were not adversely affected by large-scale duplications confounding the accurate assembly of trace sequence reads. For each species we aligned the nucleotide sequences of *Abpa *genes' second exons with all available trace sequences using MegaBLAST. For those 2-fold coverage genome assemblies (for armadillo, tree shrew, squirrel, microbat and cat) with only single *Abpa *(pseudo)genes, only 5.6 traces on average (range 2–14), were returned (representing, on average, 6.4 × 10^-7 ^of sequences in the trace archive for these species). For rabbit, by contrast, 140 traces were returned, on average (representing 1.5 × 10^-5 ^of rabbit sequences in the trace archive). The trace archive thus contains evidence for approximately 20-fold more *Abpa *genes or pseudogenes in the rabbit genome than present in the genomes of tree shrew, squirrel, microbat and cat despite these all, including rabbit, having been sequenced at 2-fold coverage. Further evaluation to confirm ascertainment of all gene copies by synteny-based methods is not possible with low-coverage genomes owing to contigs not being placed on chromosomes.

### Positive selection on Abpa and Abpbg paralogs in various species

**Figure 6:**
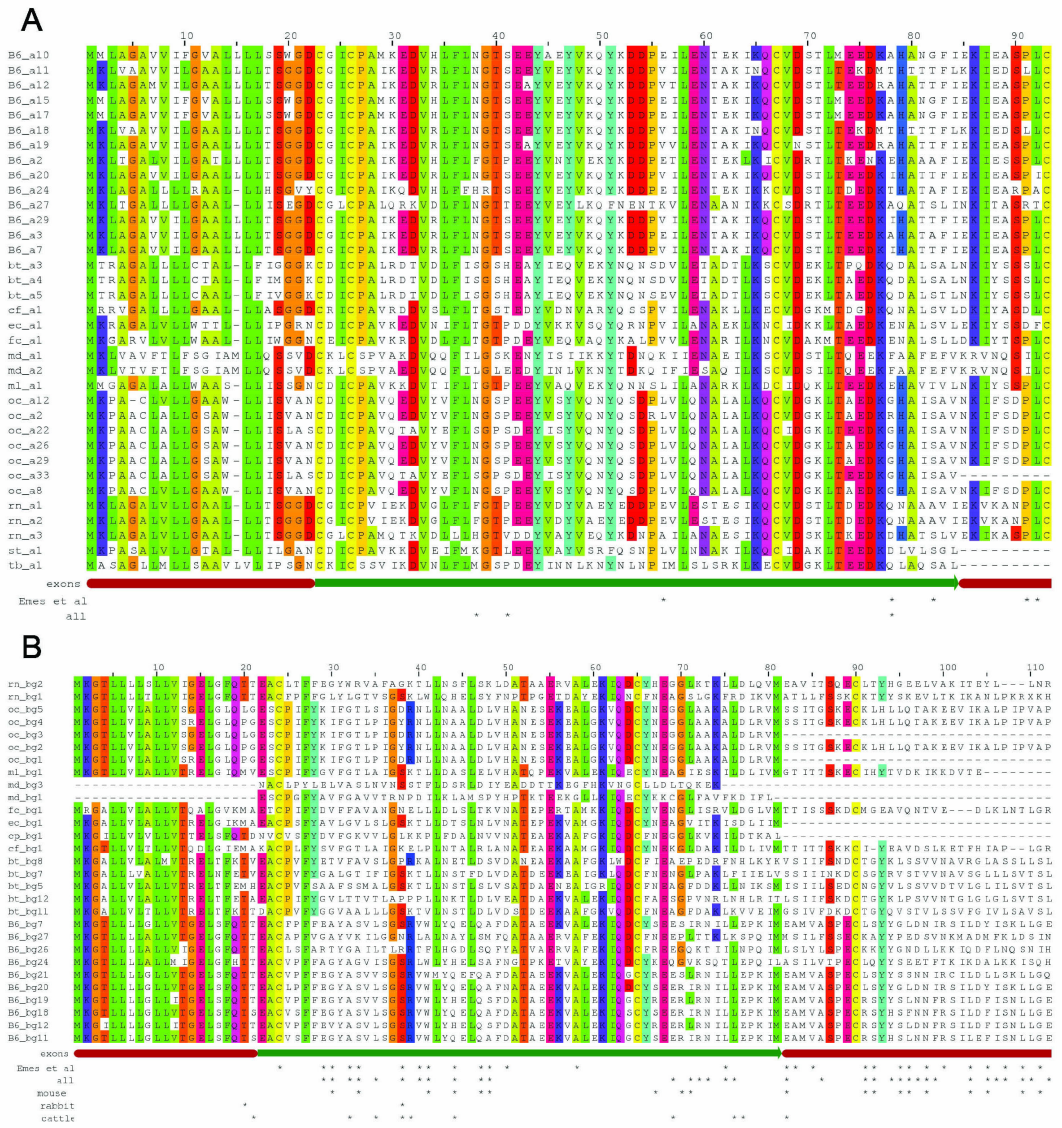
Amino acid sequence alignments for ABP alpha subunits (Panel A) and ABP beta/gamma subunits (Panel B). Asterisks below the sequence alignments denote ω^+ ^sites for all paralogues, mouse paralogues, cattle paralogues, and those previously reported [8]. Colors in columns denote identities. Symbols for the genomes represented by paralogues in the alignment are: B6, mouse genome (C57BL/6J); bt, cattle (*Bos taurus*); cf, dog (*Canis familiaris*); cp, guinea pig (Cavia porcellus); ec, horse (*Equus caballus*); md, opossum (*Monodelphis domestica*); ml, Little brown bat (*Myotis lucifugus*); oc, rabbit (*Oryctolagus cuniculus*); rn, rat (*Rattus norvegicus*); st, ground squirrel (*Spermophilus tridecemlineatus*); and, tb, tree shrew (*Tupaia belangeri*).

The presence of sites under positive selection was established for either *Abpa *or *Abpbg *genes from all mammals we considered, using a log likelihood ratio test comparing paired models M7/M8 and M1/M2 (see Additional file 4). Tests for positive selection were significant for both paired models for *Abpbg *genes (P < 10^-18^). We were especially interested to learn whether positively selected sites ("ω^+^sites") in one lineage map onto equivalent sites in other lineages. For *Abpbg *paralogues among all species investigated, we identified 34 ω^+ ^sites (Figure 6B). Of these, 21 are coincident with the sites we described previously for mouse *Abpbg *genes [8], 13 sites have not been observed previously, and seven of the original 28 sites found in mouse *Abpbg *paralogues were not found in this analysis. Considering *Abpbg *paralogues only from the mouse genome, 16 of 19 sites are coincident with those we described previously [8]. For cattle *Abpbg *paralogues, we found 10 ω^+ ^sites, four of which are shared with mouse. Two rabbit *Abpbg *ω^+ ^sites were found, one of which is shared with mouse. For *Abpa *paralogues (Figure 6A), we were unable to detect strong evidence of positive selection: M1/M2 test did not show evidence of selection (P > 0.25), while the M7/M8 test was highly significant (P < 10^-23^). One of the three potentially positively-selected sites on *Abpa *is consistent with [8] (Figure 6A). Sites undergoing positive selection were mapped onto the protein structure of cat allergen Fel dI [16]. Consistent with previous results [8], positively-selected sites were located almost exclusively on the molecules' surface and away from the dimerization interface (see Additional files 5, 6, 7, 8, 9 for locations of selected sites).

### Expression of Abp paralogues

We searched for ESTs corresponding to each of the 30 *Abpa *and 34 *Abpbg *paralogues we report here. Each of nine *Abpa *sequences and nine *Abpbg *sequences corresponded to at least one report of an EST detected in at least one tissue (Table 1). Given the large number of mouse *Abp *paralogues, it is surprising that we were able to find ESTs corresponding to so few of them. Nonetheless, some of the new *Abp *paralogues we report finding in the mm8 mouse genome build are among these (**B6_*a3*, B6_*a7*, B6_*bg7*, B6_*a12*, B6_*bg12*, B6_*a20*, B6_*bg20***). The *a27*, *bg27 *and *bg26 *paralogues correspond to the alpha, beta and gamma subunits of ABP, respectively, originally discovered and described by Karn and his colleagues as expressed in mouse salivary glands [23-25] and subsequently shown to be under the control of independent genes [6,7,9] located on mouse chromosome 7. The other paralogues are associated with ESTs expressed variously in brain olfactory lobe [17], lacrimal gland [17,26], major olfactory epithelium [17], ovary [17], prostate [17,27], testis [28], skin [29] and vomeronasal organ [17], however no corresponding proteins have yet been described.

**Table 1:** Tissue location of mouse *Abp *gene expression. *Abpa *paralogues (bold) and *Abpbg *paralogues. Expression is shown as the number of the reference publication(s) or gene accession number(s) as they appear in the reference list. Blank squares indicate an absence of evidence for expression.

	brain olfactory lobe	lacrimal gland	major olfactory epithelium	parotid gland	prostate	sublingual gland	submaxillary gland	vomero-nasal organ	Other
**B6_a2**	**[17]**	**[17, 26]**	**[17]**					**[17]**	
B6_bg2	[17]	[17, 26]	[17]					[17]	
B6_bg3		[17]						[17]	
**B6_a3**		**[26]**							
B6_bg7		[26]			[27]				
B6_a7		[26]							
B6_bg12		[26]							
**B6_a12**		**[26]**							
**B6_a29**	**[17]**	**[17, 26]**					**[17]**	**[17]**	
B6_bg20		[26]							
**B6_a20**		**[26]**							
B6_bg23									testis [28]
B6_bg24	[17]	[17]	[17]				[17]	[17]	skin [29]
**B6_a24**		**[17]**							
B6_bg26							ABP gamma [7, 9, 17]	[17]	
**B6_a26**		**[17]**	**[17]**			**[17]**	**[17]**	**[17]**	
B6_bg27		[17]	[17]	[17]	[17]	[17]	ABP beta [7, 9, 17]	[17]	ovary [17]
**B6_a27**		**[17]**	**[17]**	**[17]**	**[17]**	**[17]**	**ABP alpha [7, 9, 17]**	**[17]**	**ovary [17]**

## Discussion

### The Abp gene region within the finished mouse genome assembly

The finished mouse genome assembly (mm8) establishes the ~3 Mb *Abp *gene region as one of the most rapidly-evolving genomic sequences known. Compared with the rat, with which it shared a last common ancestor only approximately 12–24 MYA [30,31], the region has expanded 6-fold and has accumulated, by multiple duplication events, 10-fold more *Abpa *or *Abpbg *(pseudo)genes (Figure 2). The large number of evolutionarily very recent duplications generated many virtually identical sequence regions that proved impossible to assemble for the initial mouse draft genome (mm2).

Although we have, thus far, only considered gene duplication, the homogeneity of *Abpa *genes we observe might also have arisen because of non-allelic gene conversion, as previously observed for interferon-alpha [32] and globin [33] genes, for example. Nevertheless, we discount this explanation by inspecting the phylogenetic tree of ribosomal protein L23a pseudogenes (see Additional file 10), which frequently appear to have co-duplicated with *Abpa*-*Abpbg *gene pairs (Figure 2); these duplications are of recent origin since similar sequences are absent from the syntenic region in the rat. As the phylogenies of the pseudogenes and their associated *Abp *genes (Figure 3) are topologically equivalent, *Abpa*-*Abpbg *gene pairs appear to have arisen primarily by duplication, presumably via non-allelic homologous recombination, rather than by sequence homogenization after non-allelic gene conversion events. Despite expectations that mammalian genes are duplicated and fixed once every 100 million years, and acquire inactivating mutations after approximately 7 million years [18], we observe a highly-unusual expansion of a gene family from 5 to 30 members within only 7 million years.

We previously observed expression of six *Abpa *paralogues and five *Abpbg *paralogues in ten glands and other organs located predominantly in the head and neck (olfactory lobe of the brain, three salivary glands, lacrimal gland, Harderian gland, vomeronasal organ, and major olfactory epithelium; [17]). We positively identified PCR products in this expression study by comparing their sequences to those of the paralogues from which the primers were developed. Nonetheless, because the new paralogues' sequences are often closely-related to those used to develop the expression primers, we identified, using the UCSC genome browser's *in silico *PCR resource (February 2006 assembly), which of the new paralogues that would have been amplified with these primers, were they expressed. Only the original *Abpa13 *(now *B6_a29*) primer pair [17] amplified additional paralogues beyond that for which they were designed. The *Abpa13 *primers amplified two new B6 *Abpa *paralogues in the *in silico *PCR experiment (***B6_a7 ***and ***B6_a20***). However, the sequencing check of the PCR products in that study (described above) did not provide evidence that these new paralogues were expressed in any of the tissues that were positive for the original *Abpa13 *paralogue. Thus the results and conclusions reported in our original report [17] do not appear to warrant revision.

### Expression of Abp paralogues

We find evidence for expression of nine *Abpa *and nine *Abpbg *paralogues that are nearly equally divided between paralogues we originally reported [8,17] and new ones reported here (Table 1). Most expressed paralogs are found in pairs, where both partners of the pair are expressed. Surprisingly, we identified non-canonical splice sites in four paralogues previously amplified from cDNA collections by RT-PCR (B6_*bg3*, B6_*a21*, B6_*bg25 *and B6_*a26; *previously *Abpbg12, Abpa5, Abpbg9*, and *Abpa10 *[8]) and two newly-reported paralogues (**B6_*bg3 ***and **B6_*a26***). We found a single incident of non-canonical splice site changes in other taxa where the *Mus spretus *paralogue, *spr a26*, has the same non-canonical GT-AG change at the beginning of its second intron as B6_*a26*. More difficult to interpret are the ESTs (CN843804.1 and BY706510) that others have reported for B6_*bg23 *in testis, because the genome sequence for that paralogue has an early termination codon in the coding region. We aligned the B6_*bg23 *coding region with those EST sequences and verified the early termination codon in both ESTs (not shown). These unusual changes could be strain-specific changes (e.g. between C3H/HeJ and C57BL6/J) or very new pseudogenization events where control regions have not yet acquired mutations that silence transcription. Either scenario would illustrate the rapid speed at which evolution has shaped this region.

### Expansion of the Mus musculus gene cluster is rooted at the level of the subgenus Mus

We combined computational data with two experimental methods to explore the origin of the complexity of the *Abp *gene cluster within rodent taxa. Paralogue sequences derived from the mouse genome could not be definitively assigned to a single subspecies because of multiple *M. musculus *subspecies contributions to the C57BL/6J genome [34,35]. Thus, we used PCR-based gene finding data to determine sequences for many (although certainly not all) *Abpa *paralogues in the genus *Mus*. Evolutionary analysis of these sequences groups the two *M. pahari *paralogues as outgroups of clades formed by paralogues of the subgenus *Mus*. Neither of the two paralogues that we found in *Apodemus *nor any of the three *Abpa *genes found in the rat genome shows this property, suggesting that the impressive gene family expansion seen in *Mus musculus *began in an ancestor common to *M. pahari *and the subgenus *Mus*. Further, interrogating Southern blots with probes developed from the two *M. pahari *paralogues revealed other *M. pahari *paralogues that were not seen when the blots were probed with *M. m. domesticus *probes and that were not discovered in the PCR-based gene finding experiments. These could reflect an expansion of the *Abpa *gene cluster that is, at least in part, unique to *M. pahari*. This suggests that the common ancestor of *M. pahari *and the subgenus *Mus *possessed a cluster of genes, some of which may have been duplicated separately in both the subgenus *Mus *and *M. pahari *lineages and some of which may have been expanded in the *M. pahari *lineage alone. In the event that their sequences become available, the additional bands in *M. pahari *Southern blots may be associated with other clusters of subgenus *Mus *paralogues. While there may be as yet undiscovered *Apodemus *lineage-specific paralogues, these would not be close relatives of *Mus *genes owing to our inability to detect them by either of the methods we used.

The genome of the last common ancestor of *Mus *and *M. pahari *harbored only approximately five *Abpa *genes. This conclusion is drawn from the branching orders of the two sequenced *M. pahari Abpa *genes and the 30 mouse genome *Abpa *genes and pseudogenes (Figure 2; Figure 3). Of five clades, two possess *M. pahari *genes as outgroups, flanking numerous *Mus*-specific genes as ingroups. The genes from each of the five clades lie in contiguous genomic sequence (Figure 2) implying that sequence duplications of *Abpa *(and, by inference, their partner *Abpbg *genes) generated neighboring tandem duplications. From the *Abpa *phylogeny (Figure 3), and from their order on the chromosome (Figure 2), we predict that of the five *Abpa *genes predicted to be in the common ancestor of *Mus *and *M. pahari *one duplicated to generate B6_*a1 *and B6_*a2 *(formerly *Abpa1 *and *Abpa2 *[8]), a second duplicated on numerous occasions leading to **B6_*a3*-***23 *(all herein are new paralogues except B6_*a21-23 *which were previously numbered as *Abpa5-7 *[8]) and B6_*a28*-*29 *(previously *Abpa12 *and *Abpa13*), a third and a fourth (corresponding to B6_*a24 *and B6_*a25; *formerly *Abpa8 *and *Abpa9 *[8]) remained unduplicated, and the fifth duplicated to yield B6_*a26*-*27 *and B6_*a30 *(formerly *Abpa10-11 *and *Abpa14 *[8]), all in the *Mus *lineage and within an estimated time span of 7 MY.

Deletion, as well as duplication, events are likely to have shaped this region of the mouse genome. Establishing evidence for this is more problematic for unassembled genomes for which it is unknown whether the absence of a gene represents a deletion or simply a lack of sequencing of that region. However, we reject the possibility that rapid birth and death (pseudogenisation) processes are sufficient to explain our striking finding of independent expansions. First, searches of the trace server archives failed to find additional sequences whose high duplication content confounds assembly. Second, the ability of our prediction algorithms to identify divergent genes and pseudogenes (K_S _> 0.97 between rabbit genes and their single ortholog) in distant species provides confidence in their ability to identify even rapidly evolving genes. Third, we have found evidence for retained, but potentially pseudogenized genes that have changed splice sites. Thus, the speed of evolution of this genomic region would not be expected to result in removal of all inactive paralogous gene copies. Nevertheless, in those lineages that have experienced much gene duplication, it is likely that many deletions of genes, by non-allelic homologous recombination, have also occurred.

The picture arising from these data suggests a fascinating scenario in which *Abp *duplications occurred independently in the three lineages leading to *Rattus*, to *Apodemus *and to *Mus*. This was followed by a more complex evolutionary history in the genus *Mus*, in which at least two *Abpa *genes further duplicated to produce additional paralogues in the subgenus *Mus *lineage while other paralogues duplicated independently in the *M. pahari *lineage. Additionally interesting points include: 1) *M. caroli *appears to have duplicated its *Abpa27 *paralogue independently of the other species, suggesting that unique gene duplications may have taken place in some lineages of the subgenus; 2) An orthologue of *Abpa30 *was observed in each species we investigated, in spite of the fact that it is one of the few *Abpa *paralogues unpaired with an *Abpbg *in the mouse genome and is a pseudogene; 3) The *M. m. musculus Abpa27 *paralogue unexpectedly lies outside the *M. spretus *node. This was described previously by Karn et al. [11], who noted an unexpected resemblance between the Palearctic species (*M. m. musculus *excluded) when comparing divergence levels between an *Abpa *intron and single copy number DNA. They hypothesized that this pattern was due to secondary genetic exchanges along the lineages leading to the Palearctic species.

### Independent expansions of Abp have occurred in non-rodent taxa

Our prediction of *Abp *genes in *Monodelphis *demonstrates that these were not a eutherian invention, but were present in the last common ancestor of eutherians and metatherians, approximately 180 MYA. The presence of, at most, one *Abpa *or *Abpbg *gene in most eutherian genomes is most parsimoniously explained by only single versions of these genes in the genome of the earliest eutherian mammal. This single *Abpa*-*Abpbg *gene pair appears to have been conserved in active form in most mammalian lineages (e.g., cat and dog), but was silenced by pseudogenization in primates (represented in this study by bushbaby, chimpanzee and human). By contrast, *Abp *evolutionary history in *Mus*, *Rattus*, *Apodemus*, rabbit and cattle lineages is characterized by rapid and independent expansions of these gene families by gene duplication, with some of the paralogues remaining active and some likely being silenced.

We believe it unlikely that the large numbers of gene predictions in the rabbit assembly arose solely because of errors arising from two-fold statistical coverage. The nucleotide error rate and heterozygosity present in the animals sequenced, taken together, are insufficient to explain the high number of sequence-different paralogues we found. Indeed, the ~74% genome sampling from two-fold coverage sequencing [36] should result in an under-estimate, not an over-estimate, of the gene count. Finally, in this study we have inspected a large number [13] of two-fold coverage genome sequences, all of which have been sequenced and assembled using similar methods, and yet for these we often observe either one or no paralogues. In particular, we discount the possibility that the two-fold coverage genome of rabbit might, by chance, have sampled a single locus to generate a total of 38 sequence-distinct *Abpa *sequences (Figure 5).

Mouse is the only organism in which an extensive survey of *Abp *gene expression has been performed [17]. Expression of many *Abpa *and *Abpbg *paralogues were noted, primarily in glands of the face and neck. Expression in the organs otherwise appeared to be restricted to prostate and ovaries; only a single EST has been found in mouse skin. The single *Abpa*/*Abpbg *gene pairs in the house cat (*Fel dI*; [37]) and, possibly, the dog [38] are expressed in salivary glands and sebaceous glands of the skin. Both the cat [39] and the mouse [5] coat their pelts with *Abp *during grooming. Cattle *Abp *sequences and similar sequences in sheep and goat have been found in skin EST libraries (see Additional file 11). Skin glands are known to be pheromone sources in cattle [40] and the minimal *Abp *gene expression in mouse skin suggests that this source has been supplanted in rodents by the complex, multi-gland expression in the face and neck [17]. ABP thus provides the potential to present an olfactory profile for an animal of the same or a closely related species. In primates, however, these genes are conspicuously silent, suggesting a shift to a reliance on visual cues.

It is clear that *Abp *genes have diverged widely through evolutionary time, both in sequence and in gene copy number, and remain under the influence of positive selection, which has shaped the gene family in rodents. Other gene families shaped by positive selection include those involved in chemosensation, pathogen defense and sexual identification [3]. We previously proposed that *Abp *is involved in chemosensation and/or sexual identification [4,5]. Different organisms have devised different physiological mechanisms for accomplishing this function [41] and this is reflected in the evolutionary history of the relevant genes (e.g., lineage-specific duplications in V1R vomeronasal receptors [42-44]). Both the finding of different positively-selected sites in different organisms with *Abp *expansions, as well as the independent gene duplication events, reflect the effect these diverse mechanisms of evolutionary adaptation have exerted on *Abpa*.

## Conclusion

We identified 30 *Abpa *and 34 *Abpbg *genes or pseudogenes in a 3 Mb region of mouse chromosome 7. These genes occur in pairs that have arisen primarily by independent bursts of tandem duplication from approximately five *Abp *genes found in the last common ancestor of the subgenus *Mus*. At least one *Abpa *and *Abpbg *gene was present in the last common ancestor of eutherians and metatherians, approximately 180 MYA, and this single *Abpa*-*Abpbg *gene pair has been conserved in active form in most mammalian lineages, but was silenced by pseudogenization in primates. By contrast, *Abp *evolutionary history in *Mus*, *Rattus*, *Apodemus*, rabbit and cattle lineages is characterized by rapid and independent expansions of these gene families by gene duplication, with some of the paralogues remaining active and some likely becoming silenced. *Abp *genes have diverged widely through evolutionary time, both in sequence and in gene copy number, and remain under the influence of positive selection.

## Methods

### Predicting Abpa and Abpbg genes from the finished mm8 mouse genome assembly

Using CLUSTALW [45], we constructed multiple sequence alignments of genomic DNA encompassing the previously known mouse *Abpa *or *Abpbg *genes. These two alignments were unambiguous owing to the low divergences among these paralogues. In order to predict additional genes, hidden Markov models [46] were constructed from these alignments and then compared against both strands of the mouse and rat *Abp *genomic regions (mm8 for mouse; rn4 for rat). Unusually, the prediction of highly divergent *Abp *coding exons is greatly assisted by the lower divergence of neighboring intronic sequence [8]. High scoring alignments were re-aligned against the previous multiple sequence alignments. Manual curation of multiple genomic DNA sequence alignments was performed to ensure: (a) that the third exon, containing only a short portion of rapidly evolving coding sequence, was predicted correctly and without exons from different loci being inadvertently joined in erroneous gene predictions, and (b) that we were able to identify splice site consensus dinucleotides.

### Predicting Abpa and Abpbg genes from other mammalian genome assemblies

The following 17 genome sequence assemblies were also searched for the presence of *Abpa *or *Abpbg *orthologous genes: Guinea pig (*Cavia porcellus*); Ground squirrel (*Spermophilus tridecemlineatus*); European rabbit (*Oryctolagus cuniculus*); Tree Shrew (*Tupaia belangeri*); Bushbaby (*Otolemur garnetti*); Common Shrew (*Sorex araneus*); European hedgehog (*Erinaceus europaeus*); Little brown bat (*Myotis lucifugus*); Cattle (*Bos taurus*) at 7.1-fold coverage [47]; Cat (*Felis catus*); Dog (*Canis familiaris*) at 7.6-fold coverage; Nine-banded armadillo (*Dasypus novemcinctus*); Lesser hedgehog (tenrec; *Echinops telfairi*); African savannah elephant (*Loxodonta africana*); Horse (*Equus caballus*) at 6.8-fold coverage; South American opossum (*Monodelphis domestica*) at 6.5-fold coverage; and Duck-billed platypus (*Ornithorhynchus anatinus*) at 6-fold coverage [48]. Except where indicated, these data were obtained from the Broad Institute, MA [49] and represent approximately 2-fold statistical coverage of the genomes. To these, we added our findings on mouse, *Apodemus*, rat, human, chimpanzee, and macaque (*Macaca mulatta*) making a total of 23 species. For a phylogeny of these mammalian species (excepting horse) see Margulies et al. [22].

*Abpa *and *Abpbg *transcripts in these genomes were predicted on the basis of homology to a set of template protein sequences (see below) using the program Exonerate [50]. Transcript prediction proceeded in two steps: (1) Genomic regions containing putative transcripts of *Abp *were identified first using Exonerate in heuristic mode (options: – maxintron 50000 – proteinwordthreshold 3 – proteinhspdropoff 5 – proteinwordlen 5 – score 80); and, (2) Transcripts were then predicted using Exonerate in Genewise mode (-exhaustive) in all regions identified in step 1, but extended by 150 kb on either side. Identical predictions of transcripts were removed and overlapping transcripts were combined into individual genes.

For each genome, prediction of *Abp *genes was initiated with experimentally well-established mouse α and β/γ peptide reference sequences (NP_987098, NP_033726, NP_919319, and NP_840093). After a first round of gene prediction, predictions arising from all species' genomes were added to these four reference sequences (above), and the entire process of gene prediction was repeated. Parameters used for Exonerate searches were optimized specifically for *Abp *gene predictions resulting, for each species, in sequences with matched intron-exon boundaries. Further manual intervention ensured, for example, that dog predictions were orthologous to those in the house cat (*Fel dI*).

Exonerate often failed to predict the short third exon of *Abpa *genes (~9 codons). In order to arrive at complete transcripts, we re-predicted all transcripts with Genewise [51] using separate hidden Markov models of the first two exons and of the third exon. If the first two exons were successfully predicted, a search for the third exon was initiated in the 5 kb downstream segment. On success, the two predictions were combined into a single transcript. The hidden Markov models were constructed using HMMer [52] from the coding sequences of the manually curated multiple sequence alignment of the mouse and rat *Abp *genomic DNA alignments (including novel predictions from the mm8 mouse genome assembly). This step also served as a filter for spurious predictions because those not yielding alignments using Genewise were discarded. Genewise exhibits a bias towards producing artefactual undisrupted coding sequences when faced with frameshifts or in-frame stop codons, which were likewise culled by manual curation. In order to decrease the possibility of missing paralogues in unassembled sequence data, we looked for additional copies with species-specific searches of the trace archive used MegaBLAST at the National Center for Biotechnology Information [53] and default parameters, as well as using evidence from known cDNA or EST sequences. This was also used to search for evidence of expression of paralogues with non-canonical splice sites.

*Abp *gene predictions in the target genome were classified as members of either the *Abpa *or *Abpbg *family using HMMer and HMMs derived from mouse and rat *Abp *coding sequences (see above). Amino acid sequences of members of the *Abpa *and *Abpbg *families were multiply aligned using MUSCLE [54]. Nucleotide alignments were derived from the amino acid sequence alignment. To clearly distinguish mouse genome *Abp *paralogues predicted here from those previously reported [8] and from those derived from other species/subspecies, we designate our current paralogues with the prefix B6, in reference to the C57BL/6J strain whose genome has been sequenced. Predictions with frameshifts, in-frame stop codons, or non-canonical splice sites were labeled as pseudogenes.

### Gene finding in closely-related murid rodents

Genomic DNA was obtained from Jackson Laboratories (Bar Harbor, ME) for ZALENDE/EiJ, (*M. m. domesticus*), CZECH/II (*M. m. musculus*), CAST/EiJ (*M. m. castaneus*), *M. caroli*, SPRET/EiJ (*M. spretus*), and *M. pahari*. Genomic *R. norvegicus *DNA was obtained from Bioline (Randolph, MA). *Apodemus sylvaticus *genomic DNA was obtained from the collections of the Department of Zoology, Charles University in Prague.

Our strategy for gene finding involved developing primers capable of amplifying *Abpa *gene sequences in one or more of a variety of rodent taxa. First, we amplified *Abpa *sequences from the genomic DNAs listed above using the primer sets we developed previously for published mouse and rat paralogues [14,17]. Next, we performed multiple alignments of the sequences we obtained in the new taxa, and those identified in the mouse and rat genomes, to identify additional unique and conserved primer sites. Third, we made these new primer sets and tested them on the various taxa. In the process, we also mixed and matched forward and reverse primers from the old and new sets for additional combinations. Finally, we again aligned the sequences we obtained and repeated the process.

PCR was performed with a 62°C annealing temperature and 20 second stages for 25 cycles. Products were assayed by agarose gel electrophoresis. Unincorporated primers were removed using a QiaQuick PCR clean-up kit (Qiagen, Valencia, CA.) and products were sequenced by MC Labs (San Francisco, CA).

We amplified and sequenced genomic DNA from nine of the fourteen paralogues reported by Emes et al [8] in five taxa from the subgenus *Mus, M. pahari*, and *Apodemus*. Sequences represented much of these genes' exons 2 and 3 and all of their intervening second intron. It should be noted that intron sequences have not been previously reported for *Apodemus *paralogues and thus the sequences we report here should not be confused with the cDNA sequences we used in an earlier study [8].

### Evolutionary analyses

*Abpa *intron 2 sequences from *Mus*, *Apodemus*, *Rattus*, and rabbit were aligned using CLUSTALX [55,56]. Phylogenetic trees were constructed from the alignments using the program PAUP* [57] and these were displayed in TreeView [58]. In PAUP*, neighbor-joining distance parameters with Jukes-Cantor correction and random-seeding were used to calculate divergences between rodent sequences and to create trees with proportional branch lengths. These were compared to 1000-replicated bootstrap distance trees to validate an essentially identical topology. The bootstrap values from the 1000-replicate tree are related to the NJ tree as indicated in the figure legend.

After manual reconciliation of gene and species trees, we estimated the ages of B6 in-paralogues by normalizing their divergences (in percent identity) to their divergence with their inferred *M. pahari *or *M. caroli *ortholog, and multiplying by the estimated divergence time of *M. pahari *or *M. caroli *lineages from *M. m. domesticus *[20]. For example, the divergence time between *a26 *and *a27 *was calulated as:

$$\text{Divergence time }(\text{MYA})=\frac{\text{d}(a26-a27)\times 7(\text{MY})}{0.5((\text{d}(a26-pah1)+\text{d}(a27-pah1))}$$

where d(*a26*-*a27*) is the divergence between B6_*a26 *and B6_*a27*, d(*a26*-pah*1*) is the divergence between B6_*a26 *and *M. pahari *paralogue 1 and d(*a27*-*pah1*) is the divergence between B6_*a27 *and *M. pahari *paralogue 1 and 7 MY is the estimated divergence time between *M. m. domesticus *and *M. pahari *(Figure 1 and [20]).

K_A _and K_S _values, synonymous substitution rates and sites under positive selection were inferred using the codeml program of the PAML package [59] and the F3X4 codon model. The presence of sites under positive selection was established for either *Abpa *or *Abpbg *genes from all mammals we considered, using a log likelihood ratio test comparing paired models M7/M8 and M1/M2. Sites were predicted to be under positive selection if they showed a posterior probability (BEB) of *P *> 0.9 in one test and at least *P *> 0.5 in the other test. Polypeptide sequences were aligned with MUSCLE [54]. The alignment was manually checked for errors and then back-translated into nucleotide sequences, removing frame shifts and in-frame stop codons. Rate analyses were always performed on the same alignment; sequences not of interest for a particular analysis were simply excluded. Phylogenetic trees were estimated using the program KITSCH from the PHYLIP package [60].

### Southern transfers

2.5 μg of genomic DNA was digested with 60 Units of EcoRI in a 100 μl reaction at 37°C for 16 hours. This enzyme was chosen because only one of the thirty *Abpa *paralogues in the mouse genome and none of the three rat paralogues exhibited an internal restriction site. Varying the EcoRI digestion over a period of 4–16 hr had no effect on the banding patterns we obtained, supporting complete and specific digestion; complete digestion was also confirmed by running 5% of the digestion on a test gel. The digestion was stopped by adding 14 μL of 1% SDS/100 mM EDTA and ethanol precipitated after the addition of 14 μl of 3 M NaAc, this was resuspended in 20 μl 3× loading buffer, and separated by electrophoresis on a 1% agarose gel in 0.5 × TBE for 2–6 hours. The gel was denatured for 30 minutes in 0.5 M NaCl/0.5 M NaOH. Transfer materials were pre-moistened in denaturation solution and transferred to Nytran nylon transfer membrane overnight in the same solution. Following transfer disassembly, the membrane was neutralized for 10 minutes in 3 M NaCl/0.5 M Tris pH 7.4 and the DNA fixed by baking at 60°C for 1 hour. The blot was pre-hybridized for 30 minutes at 60°C in 7% SDS/0.5 M PBS pH 7.4/1 mM EDTA; this solution was also used for hybridization.

Blots were probed with labeled ~200 bp PCR products amplified from *M. m. domesticus *(Zalende/EiJ) genomic DNA by PCR with primers spanning a splice junction between exon 2 and intron 2 in B6_*a2 *or B6_*a27 *or from *M. pahari *genomic DNA using similarly-located primers. B6_*a2 *and B6_*a27 *were selected because they are located close to the two extremities of the mouse ~3 Mb *Abp *genomic region (Figure 2). Probes were labeled by random-primed incorporation of α-^32^P-dATP into PCR products using a modification of the Amersham (Piscataway, NJ) Mega Prime random-primed system and protocol. For a 25 μl reaction, 10% of a 25 μl PCR reaction was used as template and was mixed with 1 μl of each of the original 10 μM forward and reverse PCR primers as well as 2.5 μl of the Mega Prime random primer mix. Unincorporated nucleotides were removed using a QiaQick PCR clean-up kit and the probe was boiled for 5 min in 5 ml of hybridization solution, followed by 2 min of incubation on ice, before being placed on the edge of the blot and incubated at 60°C overnight. Subsequently, the blot was washed with an increasingly stringent series of washes, using 5× to 0.1 × SSC and 0.1% SDS, which produced consistent bands with a decrease in background at higher stringency. Blots were exposed to Kodak x-ray film for 18 hours to 15 days between washes and were stripped for re-probing using two fifteen minute washes of boiling 0.1 × SSC/0.1% SDS. Gels were scanned with a Nikon scanner and prepared for publication using Adobe Photoshop v. 7.0.

### Expression analysis of Abp paralogues

The BLAST algorithm of the NCBI genome browser was used to identify mouse ESTs, using the *Abpa *and *Abpbg *cDNAs reported here as search strings. An EST was assigned to a particular paralogue if it had 99+% coverage with 100% identity.

## Authors' contributions

CML designed and conducted the DNA blotting (southern transfers) and probing procedures and assisted with experimental identification of *Abp *genes from rodent DNA. AH predicted *Abp *genes from sequence assemblies. TB assisted in *Abp *gene prediction in the mouse and other organisms. PM purified the DNA from *Apodemus *caught wild in the Czech republic and assisted in the PCR experiments used to obtain *Abpa *gene sequences in closely-related rodent species, including *Apodemus*. CPP reconstructed the mouse *Abp *gene region, using the new, gap-free mouse genome data and assisted in gene prediction and results interpretation. RCK (assisted by PM) developed and implemented the PCR experiments used to obtain *Abpa *paralogues from a variety of species in the subgenus/genus *Mus*. RCK also assisted with Southern blotting and performed the EST searches. All coauthors participated in manuscript preparation and review.

## Supplementary Material

Additional file 1 cDNA sequences of *Abp *genes predicted from genomic sequencing data. FASTA formatted *Abp *gene sequences. Click here for file

Additional file 2 Names, strands, and genomic locations of *Abp *genes. Table showing *Abp *genes, locations, and genomic locations. Old and new names are listed and cross-referenced. Click here for file

Additional file 3 Predicted and sequenced intron sequences for rodent and rabbit *Abpa *genes. FASTA formatted intron 2 sequences for rodent and rabbit *Abpa *genes used in constructing Figure 2. Click here for file

Additional file 4 PAML values for tests of positive selection on *Abp *genes. Table of PAML log likelihood ratio test statistics comparing models M7/M8 and M1/M2 for *Abpa *or *Abpbg *genes. Click here for file

Additional file 5 Selected sites on *Abpbg *superimposed on ribbon structure of cat Fel dI. Beta subunit backbone is shown in blue with side chains of selected amino acids colored red. Analysis was performed with all cow, rabbit, and mouse paralogs. Click here for file

Additional file 6 Selected sites on *Abpbg *superimposed on ribbon structure of cat Fel dI. Beta subunit backbone is shown in blue with side chains of selected amino acids colored red. Analysis was performed with only mouse paralogs. Click here for file

Additional file 7 Selected sites on *Abpa *superimposed on ribbon structure of cat Fel dI. Alpha subunit backbone is shown in blue with side chains of selected amino acids colored red. Analysis was performed with only rabbit paralogs. Click here for file

Additional file 8 Selected sites on *Abpbg *superimposed on ribbon structure of cat Fel dI. Beta subunit backbone is shown in blue with side chains of selected amino acids colored red. Analysis was performed with only rabbit paralogs. Click here for file

Additional file 9 Selected sites on *Abpbg *superimposed on ribbon structure of cat Fel dI. Beta subunit backbone is shown in blue with side chains of selected amino acids colored red. Analysis was performed with only cow paralogs. Click here for file

Additional file 10 L23a pseudogene tree. Neighbor-joining tree showing phylogenetic relationships of L23a pseudogenes found in the *Abp*-containing region of mouse chromosome 7. Click here for file

Additional file 11 *Abp *EST references for non-rodent taxa. References to ESTs corresponding to *Abp *genes in non-rodent taxa. Click here for file
