# Supplementary material for: Rapid bursts of androgen-binding protein (Abp) gene duplication occurred independently in diverse mammals
Source: BMC Evol Biol. 2008 Feb 12;8:46. doi: 10.1186/1471-2148-8-46 (PMC2291036; doi:10.1186/1471-2148-8-46)
Supplement: Additional file 1 — cDNA sequences of Abp genes predicted from genomic sequencing data. FASTA formatted Abp gene sequences. [file 1471-2148-8-46-S1.doc]

>dn_a1 :+:28:3269

ACCATGAGACTTGGCATTTTGTTCCTGCTGGTCACTCTCGCCCTTTGCTGCTACCAGGCCAATGCAGTGGTCTGTCCAAATGTTTTGGAAGAATTAACAGCCTTCCTAACTGGTGATGACAGTTCGTACAAGATGATACTTGAATCCTATAATCCACCGCCAGAAGATATTGAGGCAAAACTGGAAGTGAAGAAATGCCTAGATCAGATCTCTGCACCGAAAAAACAACTTATTATAAATATAACGAGAAGCATAAGAGTTTCAACATCTTGC

>og_bg1 contig_44956:+:917:1605

ACGAAGGGGGCACTGCTTATGCTGGTCTTGCTGGTGACTGGAGAGCTGAGCTTCAAGACGGCAGAAGCTTGCCCTCTTTTCTATGCAGTCTTTGCTACAATTGGCCTTGGAAATAAGCACATAATGGACATTGTCCTCAGTGAGGCCAATGCTCCCCAACCCAGAAAAGGCAGCCTTTGAAAAAGTCCAGGAATGCTACAATGAGGGGGGTCTCAAAGCAAGACGC

>abp_bushbaby5|2|2|PP :+:1121:1330

CTTAAAGTAAGCCTTTCATTCTTTATGAGAAGCTTGCCCTCTTTTCTATGCAGTCTTTGCTACAATTGGCCTTGGAAATAAGCACATAATGGACATTGTCCTCAGTGAGGCCAATGCTACCCAACCCAGAAAAGGCAGCCTTTGAAAAAGTCCAGGAATGCTACAATGAGGGGGGTCTCAAAGCCAAGACGCTGGATACGATCGCCTTG

>abp_commonshrew5|1|1|UG :+:1307:1550

ATGAGAGAAAACTTTGCTCGTGTATTGTATTTTCTTTCCCTCCATTTAGCTGACGCAGCCATAATTTGCCCAGCCCTTGCGGGAGAATTGACTGCATTTGTTACCGGCACAGCAGATGTCTATAGACTATACATTGCTCAATTTAATGCCCCTGAGGCTGTTGTGGAGGACAAGATGCGTCCGAAGGTCTGCCTCGATGAATCCGTGCCCGCTGAAGGGAGAGCACTAATTGGAAAATTTTTG

>bt_a1 scaffold8101:+:20682:22039

ATGACGCGGGCTGGAGCTCTCCTGCTGCTCTGCGCTGCCTTGCTCCTCATCACAGGCAGAAAGTTTGATGACCTCTGTCCAGCCTTGAGGGACACTGTTGACCTGTACATATCGGGAAGCCATGAGGCCTATGTTGAACAAGTTGAAAAGTATAACCAAAACTCTGATGTACTGGAAACTGCCAATACAGTAAAGAGCTGTACTGATGAGAAGTTGACACCACAGGATAAGCAGGATGCTCTCAATGTGCTGAATAAAATATACTCAAGTTCTCTCTGC

>bt_a6 scaffold1810:+:29050:30413

ATGATGCAGGCTGGAGCTGTCCTGCTGCTCTGTGCTGCCTTGCTCCTCATCGCGGGCAGAAACTGTGACATCTGCCCAGCAGTGAGGGAGGATGTTTACCTGTTTGTGAGGGGGACCCCTGAAGAATACATTGCTAAAGTGAAAGAATACAACACAAACAGTGCGATAGTGGCCAATGCCAGGAGGCTGAAAGACAGAGTTGATGAGAAGTTGACAGAAGAGGATAAGCAGAATGCTTTCAGTGTCCTGAATAAAATATACTCAAGTTCTCTCTGC

>bt_a2 scaffold2740:+:121868:123257

ATGACACGGGCTGGAGCTCTTCTGCTGCTCTGCGCTGCCTTGCTCTTCATTGTGGGCGTTAAGTGTGATGACATCTGCCCAGCCTTGAGGGACACTGTTGACCTGTTCCTATCAGGAAGCCATGAGGCCTATATTGAACAAGTTGAAAAGTATAACCAAAACTCTGATGTACTAGAAACCGCTGATACCCTGAAGAGCTGTGTTGATGAGAAGTTGACACCAGAGGATAAACAGGATACTCTCAGTGCTCTGAATAAGATATACTCAAGTTCTCTCTGC

>bt_bg7 scaffold2740:+:55274:57322

ATGAAGGGAGCACTGCTTTTGGTGGCCTTGTTGGTGACCAGAGAGCTGAACTTTGAGACACATGAGGTGGAAGCCTGCCCTGTGTTTTATGGAGCCCTTGGTACAATTTTTATTGGAAGCAAAACATTGTTGAACTCAACGTTCGATTTGGTTGATGCTACTGATGAGGAAAAGGAAGCTATTGGAAAACTCCAGGATTGCTTCAATGAGAATGGACTTCCTGCCAAGCTTTTCATTATAGAACTTGTGAGTTCCATCATTATCAATAAGGATTGCTCTGGCTATAGAGTGTCCACAGTGGTGAATGCTGTTTCAGGATTACTTCTCAGTGTAACGTCTTTG

>bg_bg6 scaffold2740:+:13416:16115

ATGAAGGGAGCATTGCTTGTTCTGGCCTTTCTGGTGACCTGAGAGCTTACCTTCAAGATGTGTGAGGTCTGCCCTATTTTTTATAAAGCCCTTGGTATTGTGAGCCTTGGACTCCCAAGTAGAGCATTGAATGACAACTTCGATTAGATTGATGCTGTGCTGTGCTTAGTCGATAGATTGATGCTAATGAAACAGAAAAAGCAGCCATTGGAAAAATCCAGGATTGCTTCAAAGAGGACCTGAAAACAGGTTTAATTATCGCTTCTGTCACTTTTAGTGACATTTGCTCTGGCTATAAAGTGTCCTCAGTGGTGAATGCCTTTATTGGATTCAGTTCTAGTCTGCTGTCTATG

>bt_bg8 scaffold5723:+:89716:91772

ATGAAGGGAGCACTGCTTGTGCTGGCCTTGATGGTGACCAGAGAGCTGACCTTCAAGACACATGAGGTGGAAGCCTGCCCTGTGTTTTATGAAACCGTTTTTGCTGTGAGCCTTGGACTCCCACGTAAAGCATTGAATGAAACCTTGGATTCGGTCGATGCTAATGAAGCTGAAAAAGCAGCCTTTGGAAAACTCTGGGATTGCTTCATTGAAGCAGAACCTGAAGACAGGTTTAATCATCTGAAATATAAGGTGTCTATCATCTTCAGTAATGATTGCACCGGCTATAAATTGTCCTCGGTGGTGAATGCTGTTAGAGGATTGGCTTCCAGTCTGCTCTCTTTG

>bt_bg9 scaffold5723:+:28905:30967

ATGAAGGGAGCACTGCTTGTACTGGCCTTGCTGGTGACCAGAGAGCTGACCTTCAAGACAACGGAAGCCTGCCCTGTGTTTTATGAAGTCCTTAGTGCTGTGATCCTTGGACTCCCAAGTAAAGCATTGAACGAAACCTTGGATTCGATTGATTTAGCATCAGTGAAGCTGAAAAAGCAGCTGCTGAAAAAATCCGGCAGTGCTTTGTTGAAGCAGGACCTGAGAACAGGCTTATTGATCTGAAATTTAAGCTGTCCATCATCTTCAGTAATGATTGCATTGGCTATAAATTGTCCTTGGTGGTGAATGGTGTTAGAGGATTGGCTTCCAATCTGCTATCTTTG

>bt_bg10 scaffold5723:-:38084:40190

ATGAAGGGAGCACTGCTTCTGCTGGCCTTGCTAGTGACCAGAGAGTTGACCTTTGAGATGTTTGAGGCAGAAGCCTGCCCTATATTTTATGGAATGTTTGGCACAGTGGGCCTTGGAAACAAAACACTGTTAAACACAACACTCGATTTGGTTGATGCTACTGTTGCAGAAAAAAACAGCTCTAAGAAAAATCCAAGATTGCTCCAATGAGGTGGGATTTGATGCCTAAGTGTCAGATCTGAAACTCACAGCTTCCATCAACTTCAGCAAGGATTGCATCAAATATTTAGTGTCTTTGCTGGTGAGTGCAAATCTTCCCTGTTCTCATTCTCAACTGAAACTG

>bt_a5 scaffold2740:-:32881:34255

ATGACGCGGGCTGGAGCTCTCCTGCTGCTCTGCGCTGCCTTGCTCTTCATCGTGGGCGGAAAGTGTGATGACATCTGCCCAGCCTTGAGGGACACTGTTGACCTGTTCATATCAGGAAGCCATGAGGCCTATATTGAACAAGTTGAAAAGTATAACCAAAACTCTGAGGTACTGGAAACTGCCGATACCCTGAAGAGCTGTGTTGATGAGAAGTTGACAGCGGAGGATAAGCAGGATGCTCTGAGCACTCTGAATAAAATATACTCAAGTTCTCTCTGC

>bt_a4 scaffold2740:-:20636:22020

ATGACGCGGGCTGGAGCTCTCCTGCTGCTCTGCACTGCCTTGCTCTTCATCATGGGCGGGAAGTGTGATGACATCTGCCCAGCCTTGAGGGACACTGTTGACCTGTTCATATCGGGAAGCCATGAGGCTTATATTGAACAAGTTGAAAAGTATAACCAAAACTCTGATGTACTGGAAACAGCCGATACCCTGAAGAGCTGTGTTGATGAGAAGTTGACAGCGGAGGATAAGCAGGATGCTCTCAGTGCTCTGAATAAAATATACTCAAGTTCTCTCTGC

>bt_a3 scaffold7757:-:59233:60613

ATGACGCGGGCTGGAGCTCTCCTGCTGCTCTGCACTGCCTTGCTCTTCATCGGGGGTGGAAAGTGTGATGACATCTGCCCAGCCTTGAGGGACACTGTTGACCTGTTCATATCAGGAAGTCATGAGGCCTATATTGAACAAGTTGAAAAGTATAACCAAAACTCTGATGTACTGGAAACAGCTGATACCCTGAAGAGCTGTGTTGATGAGAAGTTGACACCACAGGATAAGCAGGATGCTCTCAGTGCTCTGAATAAAATATACTCAAGTTCTCTCTGT

>bt_bg11 scaffold1810:-:26845:28863

ATGAAGGGAGCACTGCTTGTGCTGACCCTGCTGGTGACCAGAGAGCTGACCTTCAAGACGACTGACGCCTGCCCTGTGTTTTATGGCGGGGTTGCTGCGCTGTTACTTGGAAGCAAAACAGTGTTGAATTCAACGCTCGATTTGGTTGATTCTACTGATGAGGAAAAGGCTGCCTTTGGAAAAGTCCAGGATTGCTTCAATGAGGCGGGATTTGATGCCAAGCTGAAAGTTGTAGAAATCATGGGTTCCATCGTCTTTGACAAGGATTGCACCGGCTATCAAGTGTCCACGGTTCTGAGCAGTGTTTTTGGAATAGTTCTCAGTGCAGTCTCTTTG

>bt_bg2 scaffold8101:-:160895:162964

ATGAAGGGAGCACTGCTTGTGCTGGCCTTGATGGTGACCAGAGAGCTGATCTTCCAGACAACGGAAGCCTGCCCTGTGTTTTATGGAGTGTTTGGCACACTGATCCTTGGAAGCAAAACGTTGTTGAATACAACACTTGATTTTGTTGCTGCTACCGATGAGGAAAAAGCAGCTCTAGGAAAAATCCAGGATTGCTATGATGAGGCGGGATTTGATGACAAGGTTTTGGATCTGCTACTCATGGCTTCCATCAACTTGAGTACGGATTGCATCAAACATTCAGTGTCCTCGGTGGTGAATTCCATTTTCTGAGCCATACTCAGTATGCTCTCTTTG

>bt_bg12 chr18:-:11643:14161

ATGAAGGGAGCACTGCTTGTGCTGGCCTTGCTGGTGACCAGAGAGCTGACTTTTGAGACTCGTGAGGCCGAAGCCTGCCCTATTTTTTATGGAGTCCTTACCACTGTAACCCTTGCATTACCACCGCCATTGTTGAACAAAACCCTCGATTTGGTTGAAGCTACTGATGCAGAAAAAGTAGCACTAGAAAAAATCCAGGATTGCTTCGCTGAGAGTGGACCTGTGAACAGGTTGAATCATCTGAGAATCACGCTTTCCATCATCTTCAGTAAAGACTGCACTGGCTATAAGTTGCCCTCGGTGGTGAACACTGGTTTAGGATTAGGTCTCAGTGTGACATCTTTG

>bt_bg1 scaffold1810:-:16363057:16363800

ATGAAGGGAGCACTGCTTGTGCTGGCCCTGCTGATGACCAGAGAGCTGACCTTCAAGATGATTGAGGTAGAAGCCTGCCCTGCCTTTTATGCTCTGCTTTCTGGTGTTAGTTGGAAATGAAACATTGTTGAACTCAACATTGGATTTGGTTGATGCTACTGATGAAGGCAGGCTTTGGAAAAAATCCAAGATTGCTTCAATGAGATTTAATGCCAAGCTGCATATCACACAAATTGTGGTAATT

>bt_bg5 scaffold1810:-:58369:60419

ATGAAGGGAGCACTGCTTGTGCTGGCCTTGCTGGTGACCAGAGAGCTGACCTTTGAGATGCATGAGGCCAAAGCCTGCCCTGTGTTTAGTGCAGCGTTTAGCTCAATGGCCCTTGGAAGCAAAACATTGTTGAACTCAACGCTCAGTTTGGTCAGTGCTACTGATGCAGAAAATGAAGCTATAGGAAGAATCCAGGATTGCTTCAATGAGGCGGGATTTGATGACAAACTGTTGAATATAAAAAGCATGATTTCCATCATCTTGAGTGAGGATTGCAATGGCTATGTACTGTCCTCAGTGGTGGATACTGTTTTAGGATTAATTCTCAGTGTGACATCTTTG

>bt_bg4 scaffold1810:-:90204:90978

ATGAAGGGAGCACTGCTTGTGCTGGCCTTGATGGTAACTAGAGAGCTCACCTTTGAGATGCGTGAGGAAGCCTGCCCTGTGTTTTATGAAGTGTTTATTTAGCACAATGTCCTTTGGAAACAAGACACTGTTGGACCTGTTCCTGGGTATGGTCAGTGCTACTGATTCAGTAAAAGCTGCCATAGGAAAAATCTAGGATTGCTACAGTGAGCTGGGACTTATTTTCAAGTTTTTGGTTCTGAAATTCTTG

>bt_bg3 :+:101611:103640

ATTAAGGGAGCACTGCTTGTGCTGGCCTTGCTGGTGACCAGAGAGCTGGCCTTTGAGATGTGTGAGGTGGAAGCCTGCCCTGTTTTTTATGAAATGCAGATCATGGTGAACTTTGTATTACCAAGGGCAGCATTAAATGAAACCTTTGATTCAGTCAATGCTACAGATGAGGAAAAAGCAGCCTTTGGAAAAATCCAGGATTGCTTCATTGAAGTAGGACATCAGAACAGGGTTAACCATCTGAAATTTAAGCTCGCTATCTTCAGTAAGGATTGCACTGGCTATAGAGTGTCCACATTGGTGAATGTTATTAGAGGATTCATTTCCAGTCTGCCCTCTTTA

>cf_a1 chr1:-:120709581:120710859

ATGAGGCGGGTCGGGGCGCTGCTGCTTCTCGGGGCTGCCCTGCTCCTGGCCTCGGGCGGAGACTGTAGGATCTGCCCAGCCGTGCGGGACGATGTGAGCCTGTTCTTGACGGGCTCCACCGAGGACTACGTGGACAACGTGGCGCGGTACCAGAGCAGCCCTGTGATACTGGAAAATGCGAAACTACTGAAGGAGTGCGTGGATGGGAAAATGACAGACGGCGACAAGCAGAATGCCCTTTCGGTGCTGGATAAAATATACGCAAGTGACTTGTGT

>cf_bg1 :+:4910163:4912410

ATGAAGGGGACACTCCTCGTGCTGACCTTGCTGGTGACCCAAGACCTGGGCATCGAAATGGCGAAAGCCTGCCCCCTTTTCTATTCGGTCTTTGGTACTTTGGCCATTGGAAAGGAGCTTCCGCTGAACACGGCCCTTAGACTAGCCAATGCTACTGAAGCAGAAAAAGCAGCCATGGGAAAAATCCAGGACTGCTACAATGAGAAGGGACTCGATGCCAAGATATTGGATTTGATTGTCATGACTACCATCACCACCAGCAAGAAATGCATCTATGAAGCAGTGGACTCATTGAAGGAGACCTTCCACATAGCCCCTTTGGGGAGA

>cp_bg1 :+:509:1263

ATGAAGGGGATACTCCTTGTGCTGGTTTTGCTAGTGACCACGGAGTTGAGCTTCCAGACAGATAATGTATGTGTTAGTTTTTATGATGTTTTTGGGAAAGTGGTCTTGGGATTGAAGAAACCATTGTTTGATGCCCTTAATGTAGTAAATGCTACTGAAGCAGAAAAGGCAGCCTTTGGAAAAATCCAGGATTGTTTCAATGAAGGAGGACTCAAAGTCAAGATATTGGATACTAAGGCCTTG

>ec_a3 scaffold_71.5000001-9474437:+:971957:973268

CTGAAGCTGGCTGGTGCTCTTATGCTACTCTGTGACATCTTGTTTCTGTTCTCCAGGAGAAATTGTGACATTTGTCCAGTTGTACATAGGAATGTTAGCATATTGGGCACCACTGTCAATAATGCTAATATAGTGGCAAAATATCAAGACAACTCTGTGATGCTGCAAATGCCAGAAAACTGAAGCGCTACTTTGATGAGAAAATGACACCAGAGTATAAGGAGCATGTGCTTGATGTGCTTCCCAAGAATGAATCCTCTCTCAACTGC

>ec_a2 scaffold_71.5000001-9474437:+:928533:933256

CTGAAGCTGGCTGGTGCTCCTGTGTTGCTCTTGTCATTTCAGGGAGTGTCACCCTGCCTTTGTGGTCCAGATTGTGGCATTTGCCCAGTTAGGCATAAGGATGTTAGCTTATTACTGACAGGTACTCCTTATAGATTTACAAATGTAGTTGCAAAATATCAAGAGAAGCTTGCTGTATAGCCATAGCCAGAAAACAGAAAAACTTTGTTAATGAGAAAATGACACCAGAAAATAAGGAACAGGCACTCAATTTGCTTGAGAAAACATATTCCTCATCAGGGTGT

>ec_bg1 scaffold_71.5000001-9474437:-:616040:616807

ATGAAGGGGGCACTGCTTGTGCTGGCCTTGCTGGTGACCAGAGAGCTGGGCATCAAGATGGCGGAAGCTTGCCCGAGTTTTTATGCAGTCCTTGGTGTGTTGTCCCTTGGAAGCAAGACACTGTTGGACACCTCCCTCAATCTGGTCAATGCTACTGAACCGGAAAAAGTAGCCATGGGAAAAATCCAGGATTGCTACAATGAGGCGGGAGTCATAACCAAGATCTCGGATCTGATCATCATG

>ec_a1 :+:3862550:3863999

ATGAAGCGGGCTGGTGCTCTCGTGCTGCTCTGGACCACCTTGCTTCTGATCCCAGGCAGAAATTGTGACATTTGCCCAGCCGTGAAGGAAGATGTTAATATATTCCTGACAGGAACCCCTGATGACTATGTTAAAAAAGTTTCACAGTACCAACGCAATCCTGTAATATTGGCCAATGCTGAAAAGCTAAAGAACTGCATTGATAAGAAATTGACAGCCGAGGATAAGGAGAATGCCCTCAGTGTGCTGGAGAAAATATACTCAAGTGATTTTTGT

>ml_a1 contig_298238:-:6596:7918

ATGATGGGGGCCGGTGCCCTCGCGCTGCTCTGGGCTGCCTCCCTCCTGATCTCCAGCGGAAATTGTGACATCTGCCCAGCCGTGAAAAAAGATGTTACCATATTCCTGACGGGAACCCCAGAAGAATATGTTGCACAAGTGGAAAAATACCAAAACAACTCTTTAATATTGGCCAATGCCAGGAAACTGAAGGACTGCATTGATCAGAAATTGACAGAAGAGGATAAGGAGCATGCCGTCACCGTGCTGAATAAAATATACTCAAGTCCTTTATGT

>ml_bg1 :+:11021:13181

ATGAAGGGGACACTGCTTGTGCTGGCCTTGCTGGTGACCAGAGAGCTGGGCATCCAGATGGTGGAATCTTGCCCTATTTTTTATGGAGTCTTTGGTACGTTGGCTATTGGAAGCAAAACACTGTTGGACGCCAGCCTCGAACTGGTCCACGCTACCCAACCAGAAAAAGTAGCCTTAGAAAAAATCCAGGAATGCTACAATGAGGCGGGAATCGAATCCAAGATCTTGGATCTGATCGTCATGGGCACTATCACCACCAGCAAGGAATGCATCCACTACACAGTGGACAAGATAAAGAAGGATGTCACCGAA

>md_a3 chr1:+:425454167:425458814

TGTTCCTTATCCTGTGTTTTTCTCAAGACTGAGAATGAAGATCTTATTCTCTCTATTCCAGATTCTGAGCCTCTCTGCCCCGAACTATTAAACACCATAGATCTGTTTTTGACTGGAGAAAAAAGCACCTTCATGGATAAGGTGATGGATTACACCTCAGATAATGAAACCATAAGTGCTGCTGAGGAACTAAAAAATTGTGTGGACTCAACCATAGAGCCTCAGTATCAACAGAGCATGAATAAATATATGAATGAGATAGAGAGCTCCGAATCTTGC

>md_a1 chr1:-:322767432:322770168

ATGAAGTTAGTCGCAGTCTTCACACTGTTCAGTGGCATTGCCATGTTGTTGCAGTCTTCAGTGGACTGTAAACTATGCTCTCCTGTGGCAAAGGATGTCCAACAATTTATTCTTGGATCAAAAGaaaattatataagtataattaaaaaataCACAGATAACCAGAAAATCATAGAAAATGCAGAAATTCTGAAATCCTGTGTGGATTCAACACTGACACAAGAAGAGAAGTTTGCTGCTTTCGAATTTGTGAAGAGAGTGAACCAGAGTATTCTCTGT

>md_a2 :+:322692291:322695975

ATGAAGTTAGTCACAGTCTTCACACTGTTCAGTGGCATTGCCATGTTGTTGCAGTCTTCAGTGGACTGTAAACTATGCTCTCCTGTGGCAGAAGATGTCCAACAATTTATTCTTGGATTAGAAGAAGATTATATAAATTTAGTAAAAAACTATACAGATAAACAGATATTCATAGAAAGTGCCCAAATTCTGAAATCCTGTGTGGATTCAATACTGACACAAGAAGAGAAGTTTGCTGCTTTCGAATTTGTGAAGAGAGTGAACCAGAGTATTCTCTGT

>oc_a14 super_64544:+:9253:10579

ATGAAGCCTGCTGCTTGCCTGGTGCTGCTCGGCGCTGCCTGGCTCCTGTTCTCTCTTGCCAATTGTGACATCTGCCGGCTGTGCAGACAGCTGTTGATGAATTTCTGTCAGGACCCTCCGATGAGTATATTTCCTATGTGCAAAATTACCAAAGTGACCCGCTAGTGCTGCAAAACGCCCTTGCGCTGAAGCAGTGGGAGATGGGAAACTGACAGCAGAGGAGAAGGGGCACGCGATCAGTGCTGTGAGTAAAATCTTCTCAGATCCTCTTTGT

>oc_a29 super_118378:+:1265:2068

ATGAAGCCTGCTGCTTGCCTGGCACTGCTCGGCTCTGCCTGGCTCCTGATCTCTGTTGCCAATTGTGACATCTGCCCGGCTGTGCAGGAAGATGTTTACGTGTTCCTGAACGGATCCCCTGAAGAGTATGTTTCCTATGTGCAAAATTACCAAAGTGACCCGCTAGTGCTGCAAAACGCCCTGGCGCTGAAGCAGTGCGTTGATGGGAAACTGACAGCGGAGGACAAGGGGCACGCGATCAGTGCCGTGAATAAAATCTTCTCAGATCCTCTTTGT

>oc_a4 super_168942:+:1454:2781

ATGAAGCCTGCTGCTTGCCTGGTGCTGCTCGGCGCTGCCTGGCTCCTGTTCTCTCTTGCCAATTGTGACATCTGCCGGCTGTGCAGACAGCTGTTTATGAATTTCTGTCAGGACCCTCCGATGAGTATATTTCCTATGTGCAAAATTACCAAAGTGACCCGCTAGTGCTGCAAAACGCCCTGGCGCTGAAGCAGTGCGTGATGGGAAACTGACAGCAGAGGAGAAGGGGCACGCGATCAGTGCCGTGAGTAAAATCTTCTCAGATCCTCTTTGT

>oc_a19 super_207046:+:1713:3591

ATGAAGCCTGCTGCTTGCCTGGTGCTGCTCGGCGCTGCCTGGCTCCTGTTCTCTTGCCANNNNNNNNNNNNNNNNNNNNNNNNNNNNNNNNNNNNNNNNNNNNNNNNNNNNNNNNNCGATGAGTATATTTCCTATGTGCAAAATTACCAAAGTGACCCGCTAGTGCTGCAAAACGCCCTTGCGCTGAAGCAGTGGGAGATGGGAAACTGACAGCAGAGGAGAAGGGGCACGCGATCAGTGCTGTGAGTAAAATCTTCTCAGATCCTCTTTGT

>oc_a26 super_175060:+:14465:15270

ATGAAGCCTGCTGCTTGCCTGGCGCTGCTTGGCTCTGCCTGGCTCCTGATCTCTGTTGCCAATTGTGACATCTGCCCGGCTGTGCAGGAAGATGTTTACGTGTTCCTGAACGGATCCCCTGAAGAGTATGTTTCCTATGTGCAAAATTACCAAAGTGACCCGCTAGTGCTGCAAAACGCCCTGGCGCTGAAGCAGTGCGTTGATGGGAAACTGACAGCGGAGGATAAGGGGCACGCAATCAGTGCCGTGAATAAAATCTTCTCAGATCCTCTTTGT

>oc_a21 super_163925:+:44639:45962

ATGAAGCCTGCTGCTTGCCTGGTGCTGCTCGGCGCTGCCTGGCTCCTGTTCTCTCTTGCCAATTGTGACATCTGCCGGCTGTGCAGACAGCTGTTTATGAATTTCTGTCAGGACCCTCCGATGAGTATATTTCCTATGTGCAAAATTACCAAAGTGACCCGCTAGTGCTGCAAAACGCCCTGGCGCTGAAGCAGTGCGTGATGGGAAACTGACAGCAGAGGAGAAGGGGCACGCGATCAGTGCTGTGAGTAAAATCTCAGATCCTCTTTGT

>oc_a17 super_164348:+:10127:11446

ATGAAGCCTGCTGCTTGCCTGGTGCTGCTCGGCACTGCCTGGCTCCTGTTCTCTCTTGCCAATTGTGACATCTGCCGGCTGTGCAGACAGCTGTTTATGAATTTCTGTCAGGACCCTCCGATGAGTATATTTCCTATGTGCAAAATTACCAAAGTGACCCGCTAGTGCAGCCCTTGCGCTGAAGCAGTGCGAGATGGGAAACTGACAGCAGAGGAGAAGGGGCACGCGATCAGTGCCGTGAGTAAAATCTTCTCAGATCCTCTTTGT

>oc_a18 super_134549:+:8518:9847

ATGAAGCCTGCTGCTTGCCTGGTGCTGCTCGGCGCTGCCTGGCTCCTGTTCTCTCTTGCCAATTGTGACATCTGCCGGCTGTGCAGACAGCTGTTTATGAATTTCTGTCAGGACCCTCCGATGAGTATATTTCCTATGTGCAAAATTACCAAAGTGACCCGCTAGTGCTGCAAAACGCCCTTACGCTGAAGCAGTGGGAGATGGGAAACTGACAGCAGAGGAGAAGGGGCACGCGATCAGTGCTGTGAGTAAAATCTTCTCAGATCCTCTTTGT

>oc_a8 super_142518:+:5719:6522

ATGAAGCCTGCTGCTTGCCTGGTGCTGCTCGGCGCTGCCTGGCTCCTGATCTCTGTTGCCAATTGTGACATCTGCCCGGCTGTGCAGGAAGATGTTTACGTGTTCCTGAACGGATCCCCTGAAGAGTATGTTTCCTATGTGCAAAATTACCAAAGTGACCCGCTGGTGCTGCAAAACGCCCTGGCGCTGAAGCAGTGCGTTGATGGGAAACTGACAGCGGAGGACAAGGGGCACGCGATCAGTGCCGTGAATAAAATCTTCTCAGATCCTCTTTGT

>oc_a12 super_187678:+:1564:2363

ATGAAGCCTGCTTGCCTGGTGCTGCTCGGCGCTGCCTGGCTCCTGATCTCTGTTGCCAATTGTGACATCTGCCCGGCTGTGCAGGAAGATGTTTACGTGTTCCTGAACGGATCCCCTGAAGAGTATGTTTCCTATGTGCAAAATTACCAAAGTGACCCGCTAGTGCTGCAAAACGCCCTGGCGCTGAAGCAGTGCGTTGATGGGAAACTGACAGCGGAGGACAAGGGGCACGCGATCAGTGCCGTGAATAAAATCTTCTCAGATCCTCTTTGT

>oc_a23 super_111552:+:4560:4932

ATGAAGCCTGCTGCTTGCCTGGCGCTGCTCGGCACTGCCTGGCTCCTGATCTCTCTTGCCAATTGTGACATCTGCCGGCTGTGCAGACAGCTGTTTATGAATTTCTGTCAGGACCCTCCGATGAGTATATTTCCTATGTGCAAAATTACCAAAGTGACCCGCTAGTGCAGCCCTTGCGCTGAAGCAGTGCGAGATGGGAAACTGACAGCAGAGGAGAAGGGGCACGCGATCAGTGCCGTG

>oc_a2 super_153398:+:5731:6535

ATGAAGCCTGCTGCTTGCCTGGCGCTGCTTGGCTCTGCCTGGCTCCTGATCTCTGTTGCCAATTGTGACATCTGCCCGGCTGTGCAGGAAGATGTTTACGTGTTCCTGAATGGATCCCCTGAAGAGTATGTTTCCTATGTGCAAAATTACCAAAGTGACCGGCTAGTGCTGCAAAACGCCCTGGCGCTGAAGCAGTGCGTTGATGGGAAACTGACAGCGGAGGACAAGAGGCACGCGATCAGTGCCGTGAATAAAATCTTCTCAGATCCTCTTTGT

>oc_a16 super_137086:+:1269:2589

ATGAAGCCTGCTGCTTGCCTGGTGCTGCTCGGCGCTGCCTGGCTCCTGTTCTCTCTTGCCAATTGTGACATCTGCCGGCTGTGCAGACAGCTGTTTATGAATTTCTGTCAGGACCCTCCGATGAGTATATTTCCTATGTGCAAAATTACCAAAGTGACCCGCTAGTGCAGCCCTTGCGCTGAAGCAGTGCGAGATGGGAAACTGACAGCAGAGGAGAAGGGGCACGCGATCAGTGCCGTGAGTAAAATCTTCTCAGATCCTCTTTGT

>oc_a10 super_130945:+:1009:2334

ATGAAGCCTGCTGCTTGCCTGGTGCTGCTCGGCGCTGCCTGGCTCCTGTTCTCTGTTGCCAATTGTGACATCTGCCGGCTGTGCAGACAGCTGTTTATGAATTTCTGTCAGGACCCTCCGATGAGTATATTTCCTATGTGCAAAATTACCAAAGTGACCCGCTAGTGCAGCCCTTGCGCTGAAGCAGTGGGAGATGGGAAACTGACAGCAGAGGAGAAGGGGCACGCGATCAGTGCCGTGAGTAAAATCTTCTCAGATCCTCTTTGT

>oc_a6 super_125878:+:1753:3073

ATGAAGCCTGCTGCTTGCCTGGCGCTGCTCGGCACTGCCTGGCTCCTGATCTCTCTTGCCAATTGTGACATCTGCCGGCTGTGCAGACAGCTGTTTATGAATTTCTGTCAGGACCCTCCGATGAGTATATTTCCTATGTGCAAAATTACCAAAGTGACCCGCTAGTGCAGCCCTTGCGCTGAAGCAGTGCGAGATGGGAAACTGACAGCAGAGGAGAAGGGGCACGCGATCAGTGCCGTGAGTAAAATCTTCTCAGATCCTCTTTGT

>oc_bg2 super_203750:-:10170:12025

ATGAAGGGGACTCTGCTTGTGCTGGCCTTGCTGGTGAGCGGAGAGCTGGGCTTGCAGCCGGGAGAGAGTGGTTGCCCAATATTTTATAAGATCTTTGGGACGCTGCCCATTGGATACAGGAACCTGTTGAACGCAGCTTTGGATTTGGTCCATGCTAATGAATCGGAAAAAGAAGCCCTTGGAAAAGTCCAGGACTGCTACAATGAGGGAGGGCTTGCAGCCAAGGCCTTGGATCTGAGGGTCATGTCATCCATCACTGGCAGCAAAGAATGCAAACTCCACTTACTGCAGACAGCCAAGGAGGAAGTTATAAAGGCCCTTCCCATTCCCGTAGCACCT

>oc_a25 super_110373:-:91356:91696

ATGAAGCCTGCTGCTTGCCTGGTGCTGCTCGGCGCTGCCTGGCTCCTGTTCTCTCTTGCCAATTGTGACATCTGCCGGCTGTGCAGACAGCTGTTTATGAATTTCTGTCAGGACCCTCCGATGAGTATATTTCCTATGTGCAAAATTACCAAAGTGACCCGCTAGTGCTGCAAAACGCCCTTGCGCTGAAGCAGTGGGAGATGGGAAA

>oc_a1 super_65402:-:1358:2243

ATGAAGCCTGCTGCTTGCCTGGCGCTGCTTGGCTCTGCCTGGCTCCTGATCTCTGTTGCCAATTGTGACATCTGCCCGGCTGTGCAGAGATGTTTACGTGTTCTTGAACGGATTCTCCTGAGAGTATGTTTCCTATGTGCAAAATTACCAAAGTGACCCGCTAGTGCTGCAAACGCCNNNNNNNNNNNNNNNNNNNNNNNNNNNNNNNNNNNNNNNNNNNNNNNNNNNNNNNNNNNNNNNNNNNNNAATAAAATCTTCTCAGATCCTCTTTGT

>oc_a31 super_129236:-:5076:6394

ATGAAGCCTGCTGCTTGCCTGGTGCTGCTCGGCGCTGCCTGGCTCCTGTTCTCTCTTGCCAATTGTGACATCTGCCGGCTGTGCAGACAGCTGTTTATGAATTTCTGTCAGGACCCTCCGATGAGTATATTTCCTATGTGCAAAATTACCAAAGTGACCCGCTAGTGCAGCCCTTGCGCTGAAGCAGTGCGAGATGGGAAACTGACAGCAGAGGAGAAGGGGCACGCGATCAGTGCCGTGAGTAAAATCTTCTCAGATCCTCTTTGT

>oc_a5 super_148034:-:7463:8789

ATGAAGCCTGCTGCTTGCCTGGTGCTGCTCGGCGCTGCCTGGCTCCTGTTCTCTCTTGCCAATTGTGACATCTGCCGGCTGTGCAGACAGCTGTTTATGAATTTCTGTCAGGACCCTCCGATGCGTATATTTCCTATGTGCAAAATTACCAAAGTGACCCGCTAGTGCAGCCCTTGCGCTGAAGCAGTGCGAGATGGGCAACTGACAGCAGAGGAGAAGGGGCACGCGATCAGTGCCGTGAGTAAAACCTTCTCAGATCCTCTTTGT

>oc_a13 super_83292:-:5092:6412

ATGAAGCCTGCTGCTTGCCTGGTGCTGCTCGGCTCTGCCTGGCTCCTGATCTCTCTTGCCAGTTGTGACATCTGCCCAGCTGTGCAGACAGCTGTTTATGAATTTCTGTCAGGACCCTCCGATGAGTATATTTCCTATGTGCAAAATTACCAAAGTGACCCGCTAGTGCTGCAAAACGCCCTGGCTCTGATACAGTGCGTGATGGGAAACTGACAGCAGAGGATAAGGGGCATGCGATCAGTGCCGTGAATAAAATCTTCTCAGATCCTCTTTGT

>oc_a36 super_84618:-:683:2009

ATGAAGCCTGCTGCTTGCCTGGTGCTGCTCGGCGCTGCCTGGCTCCTGTTCTCTCTTGCCAATTGTGACATCTGCCGGCTGTGCAGACAGCTGTTTATGAATTTCTGTCAGGACCCTCCGATGCGTATATTTCCTATGTGCAAAATTACCAAAGTGACCCGCTAGTGCTGCAAAACGCCTTTGCGCTGAAGCAGTGCGAGATGGGAAACTGACAGCAGAGGAGAAGGGGCACGCGATCAGTGCTCGTGAGTAAAATCTTCTCAGATCCTCTTTGT

>oc_a37 super_200987:-:1302:2617

ATGAAGCCTGCTGCTTGCCTGGTGCTGCTCGGCGCTGCCTGGCTCCTGTTCTCTCTTGCCAATTGTGACATCTGCCGGCTGTGCAGACAGCTGTTTATGAATTTCTGTCAGGACCCTCCGATGAGTATATTTCCTATGTGCAAAATTACCAAAGTGACCCGCTAGTGCAGCCCTTGCGCTGAAGCAGTGGGAGATGGGAAACTGACAGCAGAGGAGAAGGGGCACGCGATCAGTGCCGTGAGTAAAATCTTCTCAGATCCTCTTTGT

>oc_a24 super_173890:-:39584:40909

ATGAAGCCTGCTGCTTGCCTGGTGCTGCTCGGCGCTGCCTGGCTCCTGTTCTCTCTTGCCAGTTGTGACATCTGCCGGCTGTGCAGACAGCTGTTTATGAATTTCTGTCAGGACCCTCCGATGAGTATATTTCCTATGTGCAAAATTACCAAAGTGACCCGCTAGTGCTGCAAAACGCCCTTGCGCTGAAGCAGTGGGAGATGGGAAACTGACAGCAGAGGAGAAGGGGCACGCGATCAGTGCTGTGAGTAAAACCTTCTCAGATCCTCTTTGT

>oc_a20 super_133954:-:48845:50168

ATGAAGCCTGCTGCTTGCCTGGTGCTGCTCGGCACTGCCTGGCTCCTGTTCTCTCTTGCCAATTGTGACATCTGCCGGCTGTGCAGACAGCTGTTTATGAATTTCTGTCAGGACCCTCCGATGAGTATATTTCCTATGTGCAAAATTACCAAAGTGACCCGCTAGTGCAGCCCTTGCGCTGAAGCAGTGCGAGATGGGAAACTGACAGCAGAGGAGAAGGGGCACGCGATCAGTGCCGTGAGTAAAATCTTCTCAGATCCTCTTTGT

>oc_a7 super_150613:-:9229:10555

ATGAAGCCTGCTGCTTGCCTGGTGCTGCTCGGCGCTGCCTGGCTCCTGTTCTCTCTTGCCAATTGTGACATCTGCCGGCTGTGCAGACAGCTGTTTATGAATTTCTGTCAGGACCCTCCGATGAGTATATTTCCTATGTGCAAAATTACCAAAGTGACCCGCTAGTGCTGCAAAACGCCCTGGCGCTGAAGCAGTGCGTGATGGGAAACTGACAGCAGAGGAGAAGGGGCACGCGATCAGTGCTGTGAGTAAAATCTTCTCAGATCCTCTTTGT

>oc_a15 super_80321:-:1642:5321

ATGAAGCCTGCTGCTTGCCTGGTGCTGCTCGGCCCTGCCTGGCTCCTGTTCTCTCTTGCCAATTGTGACATCTGCCGGCTGTGCAGACAGCTGTTTATGAATTTCTGTCAGGACCCTCCGATGAGTATATTTCCTATGTGCAAAATTACCAAAGTGACCCGCTAGTGCAGCCCTTGCGCTGAAGCAGTGCGAGATGGGAAACTGACAGTGGAGGAGAAGGGGCACGCGATCAGTGCCGTGCTGAGAAGCTGAGTGG

>oc_a34 super_37356:-:1562:2886

ATGAAGCCTGCTGCTTGCCTGGTGCTGCTCGGCGCTGCCTGGCTCCTGTTCTCTTGCCAATTGTGACATCTGCCGGCTGTGCAGACAGCTGTTGATGAATTTCTGTCAGGACCCTCCGATGCGTATATTTCCTATGTGCAAAATTACCAAAGTGACCCGCTAGTGCTGCAAAACGCCCTTGCGCTGAAGCAGTGGGAGATGGGAAACTGACAGCAGAGGAGAAGGGGCACGCGATCAGTGCTGTGAGTAAAATCTTCTCAGATCCTCTTTGT

>oc_a28 super_138316:-:430:1748

ATGAAGCCTGCTGCTTGCCTGGTGCTGCTCAGCGCTGCCTGGCTCCTGTTCTCTCTTGCCAATTGTGACATCTGCCGGCTGTGCAGACAGCTGTTTATGAATTTCTGTCAGGACCCTCCGATGCGTATATTTCCTATGTGCAAAATTACCAAAGTGACCCGCTAGTGCAGCCCTTGCGCTGAAGCAGTGCGAGATGGGAAACTGACAGCAGAGGAGAAGGGGCACGCGATCAGTGCCGTGAGTAAAATCTTCTCAGATCCTCTTTGT

>oc_a11 super_25061:-:5716:7053

ATGCAGCCTGCTGCTTGCCTGGTGCTGACCGTTACATAGACGCGTGCGCTCCTGTTCTCTCTTGCCAATTGTGACATCTGCCGGCTGTGCAGACAGCTGTTTATGAATTTCTGTCAGGACCCTCCGATGAGTATATTTCCTATGTGCAAAATTACCAAAGTGACCCGCTAGTGCTGCAAAACGCCCTGGCGCTGAAGCAGTGGGAGATGGGAAACTGACAGCAGAGGAGAAGGGGCACGCGATCAGTGCCGTGAGTAAAATCTTCTCAGATCCTCTTTGT

>oc_a27 super_74358:-:1621:1993

ATGAAGCCTGCTGCTTGCCTGGTGCTGCTCGGCGCTGCCTGGCTCCTGTTCTCTCTTGCCAATTGTGACATCTGCCGGCTGTGCAGACAGCTGTTGATGAATTTCTGTCAGGACCCTCCGATGAGTATATTTCCTATGTGCAAAATTACCAAAGTGACCCGCTAGTGCAGCTCTTGCGCTGAAGCAGTGCGAGATGGGAAACTGACAGCAGAGAGAATGGGGCACGCGATCAGTGCCGTG

>oc_a32 super_64951:-:811:1190

ATGAAGCCTGCTGCTTGCCTGGTGCTGCTCGGCGCTGCCTGGCTCCTGTTCTCTCTTGCCAATTGTGACATCTGCCGGCTGTGCAGACAGCTGTTTATGAATTTCTGTCAGGACCCTCCGATGAGTATATTTCCTATGTGCAAAATTACCAAAGTGACCCGCTAGTGCTGCAAAACGCCCTTGCGCTGAAGCAGTGGGAGATGGGAAACTGACAGCAGAGGAGAAGGGGCACGCGATCAGTGCTGTG

>oc_a30 super_13665:-:8598:9929

ATGAAGCCTGCTGCTTGCCTGGTGCTGCTCGGCGCTGCCTGGCTCCTGTTCTCTCTTGCCAATTGTGACATCTGCCGGCTGTGCAGACAGCTGTTTATGAATTCTGTCAGGACCCTCCGATGAGTATATTTCCTATGTGCAAAATTACCAAAGTGACCCGCTAGTGCTGCAAAACGCCCTTGCGCTGAAGCAGTGGGAGATGGGAAACTGACAGCCAGAGGAGAAGGGGCACGCGATCAGTGCTGTGAGTAAAATCTTCTCAGATCCTCTTTGT

>oc_a9 super_176488:-:378:750

ATGAAGCCTGCTGCTTGCCTGGCACTGCTCGGCGCTGCCTGGCTCCTGTTCTCTCTTGCCAATTGTGACATCTGCCGGCTGTGCAGACAGCTGTTTGTGAATTTCTGTCAGGACCCTCCGATGCGTATATTTCCTATGTGCAAAATTACCAAAGTGACCCGCTAGTGCAGCCCTTGCGCTGAAGCAGTGCGAGATGGGAAACTGACAGCAGAGGAGAAGGGGCACGCGATCAGTGCCGTG

>oc_a22 super_116683:-:118078:119396

ATGAAGCCTGCTGCTTGCCTGGCACTGCTCGGCTCTGCCTGGCTCCTGATCTCTCTTGCCAGTTGTGACATCTGCCCAGCTGTGCAGACAGCTGTTTATGAATTTCTGTCAGGACCCTCCGATGAGTATATTTCCTATGTGCAAAATTACCAAAGTGACCCGCTAGTGCTGCAAAACGCCCTGGCGCTGAAGCAGTGCATTGATGGGAAACTGACAGAGGAGGATAAGGGGCATGCAATCAGTGCCGTGAATAAAATCTTCTCAGATCCTCTTTGT

>oc_a3 super_80237:-:4094:5421

ATGAAGCCTGCTGCTTGCCTGGCACTGCTCAGCACTGCCTGGCTCCTGATCTCTGTTGCCAATTGTGACATCTGCCCGGCTGTGCAGACAGCTGTTTATGAATTTCTGTCAGGACCCTCCGATGAGTATATTTCCTATGTGCAAAATTACCAAAGTGACCCACTAGTGCTGTAAAATGCCCTGGCGCTGAAGCAGTGCGTTGATGGGAAACTGACAATGGAGGATAAGGGGCACGCGATCAGTGCTGTGAATAAAATCTTCTCAGATCCTCTTTGT

>oc_a33 super_86679:-:2201:2582

ATGAAGCCTGCTGCTTGCCTGGCACTGCTCGGCTCTGCCTGGCTCCTGATCTCTCTTGCCAGTTGTGACATCTGCCCAGCTGTGCAGACAGCTGTTTATGAATTTCTGTCAGGACCCTCCGATGAGTATATTTCCTATGTGCAAAATTACCAAAGTGACCCGCTAGTGCTGCAAAACGCCCTGGCGCTGAAGCAGTGCGTTGATGGGAAACTGACAGAGGAGGATAAGGGGCATGCGATCAGTGCCGTG

>oc_a38 super_82580:-:7480:8796

ATGAAGCCTGCTGCTTGCCTGGCGCTGCTCGGCACTGCCTGGCTCCTGTTCTCTCTTGCCAATTGTGACATCTGCCGGCTGTGCAGACAGCTGTTTATGAATTTCTGTCAGGACCCTCCGATGAGTATATTTCCTATGTGCAAAATTACCAAAGTGACCCGCTAGTGCAGCCCTTGCGCTGAAGCAGTGCGAGATGGGAAACTGACAGCAGAGGAGAAGGGGCACGCGATCAGTGCCGTGAGTAAAATCTTCTCAGATCCTCTTTGT

>oc_a35 super_111552:-:7885:9213

ATGAAGCCTGCTGCTTGCCTGGTGCTGCTCGGCGCTGCCTGGCTCCTGTTCTCTCTTGCCAATTGTGACATCTGCCGGCTGTGCAGACAGCTGTTTATGAATTTCTGTCAGGACCCTCCGATGAGTATATTTCCTATGTGCAAAATTACCAAAGTGACCCGCTAGTGCTGCAAAACGCCCTGGCGCTGAAGCAGTGCGTGATGGGAAACTGACAGCAGAGGAGAAGGGGCACGCGATCAGTGCTGTGAGTAAAATCTTCTCAGATCCTCTTTGT

>oc_bg1 super_134549:-:9823:10841

ATGAAGGGGACGCTGCTTGTGCTGGCCTTGCTGGTGAGCAGAGAGCTGGGCTTGCAGCCGGGAGAGAGTGGTTGCCCAATATTTTATAAGATCTTTGGGACGCTGCCCATTGGAGACAGGAACCTGTTGAACGCAGCTTTGGATTTGGTCCATGCTAATGAATCGGAAAAAGAAGCCCTTGGAAAAGTCCAGGACTGCTACAATGAGGGAGGGCTTGCAGCCAAGGCCTTGGATCTGAGGGTCATG

>oc_bg3 super_207046:-:12554:13570

ATGAAGGGGACTCTGCTTGTGCTGGCCTTGCTGGTGAGCGGAGAGCTGGGCTTGCAGCTGGGAGAGAGTGGTTGCCCAATATTTTATAAGATCTTTGGGACGCTGCCCATTGGAGACAGGAACCTGTTGAACGCAGCTTTGGATTTGGTCCATGCTAATGAATCGGAAAAAGAAGCCCTTGGAAAAGTCCAGGACTGCTACAATGAGGGAGGGCTTGCAGCCAAGGCCTTGGATCTGAGGGTCATG

>oc_bg5 super_176488:-:191964:193814

ATGAAGGGGACTCTGCTTGTGCTGGCCTTGCTGGTGAGCGGAGAGCTGGGCTTGCAGCTGGGAGAGAGTGGTTGCCCAATATTTTATAAGATCTTTGGGACGCTGTCCATTGGAGACAGGAACCTGTTGAACGCAGCTTTGGATTTGGTCCATGCTAATGAATCGGAAAAAGAAGCCCTTGGAAAAGTCCAGGACTGCTACAATGAGGGAGGGCTTGCAGCCAAGGCCTTGGATCTGAGGGTCATGTCATCCATCACTGGCAGCAAAGAATGCAAACTCCACTTACTGCAGACAGCCAAGGAGGAAGTTATAAAGGCCCTTCCCATTCCCGTAGCACCC

>oc_bg4 :+:104810:106665

ATGAAGGGGACGCTGCTTGTGCTGGCCTTGCTGGTGAGCAGAGAGCTGGGCTTGCAGCCGGGAGAGAGTGGTTGCCCAATATTTTATAAGATCTTTGGGACGCTGCCCATTGGATACAGGAACCTGTTGAACGCAGCTTTGGATTTGGTCCATGCTAATGAATCGGAAAAAGAAGCCCTTGGAAAAGTCCAGGACTGCTACAATGAGGGAGGGCTTGCAGCCAAGGCCTTGGATCTGAGGGTCATGTCATCCATCACTGGCAGCAAAGAATGCAAACTCCACTTACTGCAGACAGCCAAGGAGGAAGTTATAAAGGCCCTTCCCATTCCCGTAGCACCC

>st_a1 contig_534814:-:1969:2382

ATGAAGCCAGCTAGTGCTCTTGTGCTGCTGGGCACTGCTCTCCTCCTGATCTTGGGTGCAAATTGTGATATTTGCCCGGCTGTGAAAAAGGATGTTGAGATTTTCATGAAGGGTACCCTCGAAGAATATGTTGCATACGTGTCAAGATTCCAAAGTAACCCTTTAGTATTGAACAATGCTAAAATACTAAAGCAATGCATTGATGCCAAACTGACAGAAGAGGATAAGGATCTGGTGCTCAGTGGGCTG

>st_bg1 :+:1398:3504

ATGAAGGGGATACTACTTGTGCTGGCCTTGCTGGTGACCAGAGAGCTGGGCTTCCAGACTGCAGAAGCCTGCCCTCTTTTTTATGGAATCTTTTCTACATTAGCTCTTGGAAGCAAGTCATTACTGGATGCTTCCCTTGAAGTGGCTAATTTTACTGAACCAGAAAAGGCAGCCATGGAAAAAATCCAGGATTGCTACAATGAAAATGGATTATCAGCCAAGGGCTTGGATTTGATTGCCATGGTCTCCATTACTGCCAGCCCAGAATGTCTCCTCTTCTATGTGCAATCACTGAAGGACAAAATTTAAAAAGGCCTTTTCAACCTTCCATGACCAG

>tb_a1 contig_830048:-:657:1275

ATGGCGTCCGCGGGTCTTCTCATGCTGCTCTCTGCTGCTGTGTTGGTCCTGATCCCAAGTGGAAATTGTGACAAGATATGCTCATCTGTGATTAAGGACGTTAACCTGTTCCTGATGGGATCACCGGATGAATATATTAATAATTTGAAGAACTATAACTTAAATCCTATTATGCTGTCCCTATCTCGAAAGCTGAAGGAATGTGTTGATGGGAAGTTGACCGAGGAGGATAAGCAGCTAGCACAAAGTGCACTG

>abp_treeshrew5|2|2|PP contig_19463:-:2412:2660

CTAATGCTGGCTACTTCTCTTGTGCTTCTCTGTGCTCTGATGAGTGTTTTCTATGGGAATGTCATCCTTGTGATTTAGAATGTGACATCTGCTCAGTTGTACATGAGGATGTTAGCATATTCCTGACAGGCTTCTGTAATGCCAGAATTCCAAGACAACCTTGTGATGTTGCAAATGTCAGAGAACTAAAGAACTGCTTTAAAAAGAAAATGACATCAGAGAATAGGATATTTAAAATCTTTATAATT

>tb_bg1 :+:420:624

ATTTTTCTGTTTGTGTTTTCAGCGGAATCTTGCTGGATTTTTTATCAAACCTTTGGTCTCTTGGCCTCTGGAAACAGGGACCTGCTGAACAAGTCCCTTGATGAAGTCAATGCTACTGACTCAGAAAAGGCAGTCTTCGGAAAAATCCAGGATTGTTACAATGAGGCTGGAATCGCGGGCAAGCTCTCGGATGCGTTCACCATG

>B6_a3

ATGAAGCTTGCTGGTGCTGTGGTGATCCTCGGGG-CTGCCCTGCTCCTCCTGACTTCAGGGGGAGATTGTGGCATTTGCCCAGCTATAAAAGAGGATGTTCGTCTATTTTTAAACGGAACCTCAGAAGAGTATGTTGAGTACGTGAAACAATACAAA-GATGACCCTGAAATACTGGAAAATACTGCAAAAA---TCAAGCAATGTGTTGATAGCACATTGACAGAGGAAGACAAGGCACATGCAACTGCTTTCATCGAAAAAATAGAAGCCAGCCC-GCTATGT

>B6_a11

ATGAAGCTTGTTGCTGCTGTAGTGATCCTTGGGG-CTGCCCTGCTCCTCCTGACTTCAGGGGGAGATTGTGGCATTTGCCCAGCTATAAAAGAGGATGTTCGTCTATTTTTAAATGGAACCTCAGAGGAGTATGTTGAGTACGTGAAACAATACAAA-GATGACCCTGTAATACTGGAAAATACTGCAAAAA---TCAACCAATGTGTCGATAGCACTTTGACAGAGAAAGATATGACACATACAACTACTTTCTTAAAAAAGATAGAAGACAGCCT-GCTATGT

>B6_a12

ATGAAGCTTGCTGGTGCTATGGTGATCCTCGGGG-CTGCCCTGCTCCTCCTGACTTCAGGGGGAGATTGTGGCATTTGCCCAGCTATAAAAGAGGATGTTCGTCTATTTTTAAATGGAACCTCAGAAGCGTATGTTGAGTACGTGAAACAATACAAA-GATGACCCTGTAACACTGGAAAATACTGCAAAAA---TAAAGCAATGTGTCGATAGCACCTTGACAGAGGAAGACAGGGCACATGCGACTACTTTCATAGAAAAGATAGAAGCCAGCCC-ACTATGT

>B6_a29

ATGAAGCTTGCTGGTGCTGTGGTGATCCTCGGGG-CTGCCCTGCTCCTCCTGACTTCAGGAGGAGATTGTGGCATTTGCCCAGCTATAAAAGAGGATGTTCGTCTATTTTTAAATGGAACCTCAGAGGAGTATGTTGAGTACGTGAAACAATACAAA-GATGACCCTGTAATACTGGAAAATACTGCAAAAA---TCAAGCAATGTGTCGATAGCACCTTGACAGAGGAAGACAAGATACATGCAACTACTTTCATAGAAAAGATAGAAGCCAGCCC-GCTATGT

>B6_a13p

ATGATGCTTGCTGGTGCTGTGGTGATCCTCGGGG-CTGCCCTGTTCCTCCTGACTTCAGGGGAAGATTGTGGCATTTGCCCAGCTATAAAAGAGGATGTTCATCTATTTTTAAATGGAACCTCAGAAGAGTATGTTGAGTATAGAAAAAGATACAAA-GATGATCCTGAAATACTGGAAAATACTGAAAAAAA--TCAAGCAATGTGTTGATAGCACATTGATGGAGGAAGACATGGCACATACAAATGGTTTCATTGAAAAAATAGAAGCCAGCCC-TCTATGT

>B6_a14p

ATGAAGCTTGCTGGTGCTGTGGTGATCCTCGGGG-CTGCCCTCCTCCTCCTGACTTCAGGGGGAGATTGTGGCATTTGCCCAGCTATAAAAGAGGATGTTTGTCTCTTTTTAAATGGAACCTCAGAAGAGTATGTTGAGTACGTGAAACAATACAAA-AATGACCCTGAAATACTGGAAAATACTGAAAAAA---TCAAGCAATGTGTTGATAGCACATTGACAGAGAAAGACAAGGCACATGCAACTGCTTTCATCGAAAAAATAGAAGACAGGCT-GCTATGT

>B6_a15

ATGATGCTTGCTGGTGCTGTGGTGATCTTTGGGG-TTGCCCTGCTCCTCCTCTCTTCATGGGGAGATTGTGGCATTTGCCCAGCTATGAAAGAGGATGTTCATCTATTTTTAAACGGAACCTCAGAAGAGTATGTTGAGTATGTGAAACAATATAAA-GATGATCCTGAAATATTGGAAAATACTGAAAAAA---TCAAGCAATGTGTTGACAGCACATTGATGGAGGAAGACAAGGCACATGCAAATGGTTTCATTGAAAAAATAGAAGCCAGCCC-ACTATGT

>B6_a16p

ATGAAGCTTGCTGGTGCTGTGGTGATCCTCGGGG-CTGCCCTCCTCCTCCTGACTTCAGGGGGAGATTGTGGCATTTGCCCAGCTATAAAAGAGGATGTTTGTCTCTTTTTAAATGGAACCTCAGAAGAGTATGTTGAGTACGTGAAACAATACAAA-AATGACCCTGAAATACTGGAAAATACTGAAAAAA---TCAAGCAATGTGTTGATAGCACATTGACAGAGAAAGACAAGGCACATGCAACTGCTTTCATCGAAAAAATAGAAGACAGGCT-GCTATGT

>B6_a17

ATGATGCTTGCTGGTGCTGTGGTGATCTTTGGGG-TTGCCCTGCTCCTCCTCTCTTCATGGGGAGATTGTGGCATTTGCCCAGCTATGAAAGAGGATGTTCATCTATTTTTAAACGGAACCTCAGAAGAGTATGTTGAGTATGTGAAACAATATAAA-GATGATCCTGAAATATTGGAAAATACTGAAAAAA---TCAAGCAATGTGTTGACAGCACATTGATGGAGGAAGACAAGGCACATGCAAATGGTTTCATTGAAAAAATAGAAGCCAGCCC-ACTATGT

>B6_a18

ATGAAGCTTGTTGCTGCTGTAGTGATCCTTGGGG-CTGCCCTGCTCCTCCTGACTTCAGGGGGAGATTGTGGCATTTGCCCAGCTATAAAAGAGGATGTTCGTCTATTTTTAAATGGAACCTCAGAGGAGTATGTTGAGTACGTGAAACAATACAAA-GATGACCCTGTAATACTGGAAAATACTGCAAAAA---TCAACCAATGTGTCGATAGCACTTTGACAGAGAAAGATATGACACATACAACTACTTTCTTAAAAAAGATAGAAGACAGCCT-GCTATGT

>B6_a19

ATGAAGCTTGCTGGTGCTGTGGTGATCCTCGGGG-CTGCCCTGCTCCTCCTGACTTCAGGGGGAGATTGTGGCATTTGCCCAGCTATAAAAGAGGATGTTCGTCTATTTTTAAATGGAACCTCAGAAGCGTATGTTGAGTACGTGAAACAATACAAA-GATGACCCTGTAGTACTGGAAAATACTGCAAAAA---TAAAGCAATGTGTCAATAGCACCTTGACAGAGGAAGACAGGGCACATGCAACTACTTTCATAGAAAAGATAGAAGCCAGCCC-ACTATGT

>B6_a2

ATGAAGCTCACTGGTGCTCTGGTGATCCTCGGGG-CTACCCTGCTCCTCCTAACTTCAGGGGGAGATTGTGGCATTTGTCCAGCTATAAAAGAGGATGTTCATCTATTTCTTTTCGGGACCCCAGAAGAGTATGTTAATTATGTGGAGAAATACAAA-GATGACCCTGAAACACTGGAAAATACTGAAAAAC---TCAAGATATGTGTCGATAGGACGTTGACGAAGGAAAACAAGGAACATGCGGCTGCTTTTATCGAAAAAATAGAATCCAGTCC-GTTATGT

>B6_a4p

ATGAAGCTTGCTGGTGCTGTGGT--TCTTTGGGG-CTGCCCTGCTCCTCCTGACTTCAGGGGGAGATTGTGGCATTTGCCCAGCTATAAAAGAGGATGTTCGTCTATTTTTAAATGGAACCTCAGAAGAGTATGTTGAGTACGTGAAACAATACAAA-GATGACCCTTAAATACTGGAAAATACTGAAAAAA---TCAAGCAGTGTGTTGATATATTATTGACAGAGGAAGACAAGGCACATGCAACTTCTTTCATCCAAAAAATAGAAGCCAGCCCCACTATGT

>B6_a20

ATGAAGCTTGCTGGTGCTGTGGTGATCCTCGGGG-CTGCCTTGCTCCTCCTGACTTCAGGGGGAGATTGTGGCATTTGCCCAGCTATAAAAGAGGATGTTCGTCTATTTTTAAATGGAACCTCAGAGGAGTATGTTGAGTACGTGAAACAATACAAA-GATGACCCTGTAATACTGGAAAATACTGCAAAAA---TCAAGCAATGTGTCGATAGCACCTTGACAGAGGAAGACAAGATACATGCAACTACTTTCATAGAAAAGATAGAAGCCAGCCC-GATATGT

>B6_a21p

ATGAAGCTTGCTGGTGCTGTGGTGATCCTTGGGG-CTGCCCTGCTCCTCCTGACTTCAGGGGGAGATTGTGGCATTTGCCCAGCTATAAAAGAGGATGTTCACCTATTTTTAAACAGAACCTCAGAAGAGTATGTTGAGTACATGAAACAATACAAA-GATCACCCTGAAATACTGGAAAATACTGAAAAAA---TCAAGCAATGTGTTGATAGCACGTTGACAGAGGCAGACAAGGCACATGTAACTGCTTTCATCGAAAAA-TAGAAGCCAGCCA-GCTATGT

>B6_a22p

ATGAAGCTTGCTGGTGCTGTGGTGATCCTCAGG--CTGCCCTACTCCTCCTGACTTCAGGGGGAGATTGTGGCATTTGTCCAGCTATAAAAGAGGATGTTCATCTATTTTTAAATGGAACTTCAGAAGAGTATGTTGAGTACGTGAAACAATACAAA-GATGACCCTGTAATACTGGAAAATAGTGAAAAAA---TCAAGCAATGTGTTGGTAGCACCTTGACAAAGGAAGACAAGATACATGCAACTACTTTCATAGAAAAAATAGAAGCCAGCCA-GTTATGT

>B6_a23p

ATGAAGCTTGCTGGTGCTTTGATGATCCTTGAGG-CTGCCCTATTCCTCCTGACTTCAGGGGGAGATTGTGGCATTTGCCCAGCTATAAAAGAGGATGTTAATCTATTTTTTAATGGAACCTTAGAAGAGTATGTTGATTATGTGAAACAATAAAAAAGATGACACTGAAATACTGGAAAATACTGAAAAAAAA-TCAAGAAATGTGTGGATAGCACGTTGACAGAGGAAGACAAGGCACATGAAACTGCTTTCATCGAAAAAATAGAAGCCAGCAC-GGCATGT

>B6_a24

ATGAAGCTCGCTGGTGCTCTCTTGCTGCTCAGGG-CTGCCCTG---CTCCTGCATTCAGGGGTATATTGTGGCATTTGCCCAGCTATAAAACAGGATGTTCATCTATTCTTTCACAGGACCTCAGAAGAGTATGTTGAATATGTGAAACAATACAAA-GACGATCCTGAAATACTGGAAAATACTGAAAAGA---TCAAGAAATGTGTCGATAGCACATTGACAGACGAAGACAAGACACATGCAACTGCTTTCATAGAAAAAATAGAAGCCAGACC-AGCATGT

>B6_a25p

TTAAAGCTGGCTGGTGCTTTCGTGATCCTTGGGG-CTGCACTG---CTCCTGACTGCAGGGAGAGA--------TTTGCCCAGCTATAAAATAGTATATTCATCTATTTTTT-ATGGGACCCCAGAAAAGTATGTTGAGTATGTGAAAAAATACAAA-CATAACCCTGAAATAGTGGAAAATACTGAAAAAC---TCAAGAAATGTGTCCAT-TCAAGTTGACAGATGTAGACAAGGCACATGTAACTGCTTTCATCGAAGAAATAGAAGCCAGCCT-GTCATGC

>B6_a26p

ATGAAGCTCGCTGGTGCTCTCTTGCTGCTCGGGG-CTGCCCTG---CTCCTGACTTCAGGGGGAGACTGTGGCCTTTGCCCTGCTTTACAGAAAAAAGTTGATATATTTTTTAATGGGACCACTGAAGAGTATGTTGAGTACCTGAAACCATACAAT-AAAGACACTAACGTACTGGAAAATGCTGCAAATA---TCAAGAAATGTTCTGATAGGACGTTGACCGAGGAAGACAAGGCACAGGCAACCATTCTCATCAATAAAATAACAGCTAGCCG-GACATGT

>B6_a27

ATGAAGCTCACTGGTGCTCTCTTGCTGCTCGGGG-CTGCCCTG---CTCCTGATTTCAGAGGGAGATTGTGGCCTTTGCCCAGCTTTACAGAGAAAAGTTGATTTATTTTTGAATGGGACCACTGAAGAGTATGTTGAGTACCTGAAACAATTCAAT-GAAAACACTAAAGTACTGGAAAATGCTGCAAATA---TCAAGAAATGTTCTGATAGGACCTTGACCGAGGAAGACAAGGCACAGGCAACCAGTCTCATCAATAAAATAACAGCCAGCCG-GACATGT

>B6_a30p

-----------------------------------------------------------------ATTGTGGCCTTTGCCCAGTTTTACAAAAAAAAGTCCATAAATTTTTCGATGGGACCACTGAAGAGTATGTTGAGTACCTGAAACAATACAAT-AATGACCCTCTCGTACTGGAAAATGCTGCAAATA---TCAAGAAATGTTCCGATAGGATGTTGGCCGAGGAAGACAAGACGCAGGCAACCAATTTCATCAATAAAGTAACAGGCAGCCG-GTCATGT

>B6_a28p

ATGAAGCTTGCTGGTGCTGTGGTGATCCTCGGGG-CTGCCCTGATCGTCCTGACTTCAGGGGGAGATTGTGGCATTTGCCTAGCTATAAAAGAGGAGGTTCGTCTATTTTTAAACGGAACCTCAGAAGAGTATGTTGAGTATGTGAAACAATACAAA-GATGACCCTGAAATACTGGAAAATACTGCAAAAG---TCAAGCAATGTGTTGATAGCACATTGACAGAGGAAGACATAACACATGCAACTGTTTTTATCGAAAAAATAGAACCCAGCAC-ACTATGT

>B6_a5p

ATGAAGCTTGCTGGTGCTGTGGTGATTCTAGGTG-CTGCTCTGCTCCTCCTGACTTCAGGGGGGGATTGTGCCATTTGCCCAGTTATAAAAGAGGATGTTCATCTATTTTTAAACGGAATCTCATAAGAGTATGTTGAGTATGTGAAACAATACAAA-GATGACCCTGAAATACTGGAAAATACTAAAAAAAAAATCAAGCAATGTGTTGATAGCACATTGACAGAGGAAGACAAGGCACATACAACTGATTTCATCGAAAAAATAGAAGCTAGCCT-GCTATGA

>B6_a6p

ATGAAGCTTGCTGGTGCTGTGGTGATTCTCGGGGGCTGCCCTGCTCCTCCTGACTTCAGGGGGAGATTGTGGCATTTGCCCAGCTATAAAAGAGGATATTCATCTATTTTTAAATGGAACCTCAGAAGAGTATGTTGAGTATGTGAAACAATACAAA-GATGACCCTGAAATACTGGAAAATACTGAAAAAAA--TCAAGCAATGTGTTGATAGCACATTGATGGAGGAAGACAAGGCACATGCAACTGCTTTCATTGAAAAAATAGAAGCCAGCCT-GCTATGT

>B6_a7

ATGAAGCTTGCTGGTGCTGTGGTGATCCTCGGGG-CTGCCCTGCTCCTCCTGACTTCAGGAGGAGATTGTGGCATTTGCCCAGCTATAAAAGAGGATGTTCGTCTATTTTTAAATGGAACCTCAGAGGAGTATGTTGAGTACGTGAAACAATACAAA-GATGACCCTGTAATACTGGAAAATACTGCAAAAA---TCAAGCAATGTGTCGATAGCACCTTGACAGAGGAAGACAAGATACATGCAACTACTTTCATAGAAAAGATAGAAGCCAGCCC-GCTATGT

>B6_a8p

ATGATGCTTGCTGGTGCTGTGGTGATCCTCGGGG-CTGCCCTGTTCCTCCTGACTTCAGGGGAAGATTGTGGCATTTGCCCAGCTATAAAAGAGGATGTTCATCTATTTTTAAATGGAACCTCAGAAGAGTATGTTGAGTATAGAAAAAGATACAAA-GATGATCCTGAAATACTGGAAAATACTGAAAAAAA--TCAAGCAATGTGTTGATAGCACATTGATGGAGGAAGACATGGCACATACAAATGGTTTCATTGAAAAAATAGAAGCCAGCCC-TCTATGT

>B6_a9p

ATGAAGCTTGCTGGTGCTGTGGTGATCCTCGGGG-CTGCCCTCCTCCTCCTGACTTCAGGGGGAGATTGTGGCATTTGCCCAGCTATAAAAGAGGATGTTTGTCTCTTTTTAAATGGAACCTCAGAAGAGTATGTTGAGTACGTGAAACAATACAAA-AATGACCCTGAAATACTGGAAAATACTGAAAAAA---TCAAGCAATGTGTTGATAGCACATTGACAGAGAAAGACAAGGCACATGCAACTGCTTTCATCGAAAAAATAGAAGACAGGCT-GCTATGT

>B6_a10

ATGATGCTTGCTGGTGCTGTGGTGATCTTTGGGG-TTGCCCTGCTCCTCCTCTCTTCATGGGGAGATTGTGGCATTTGCCCAGCTATGAAAGAGGATGTTCATCTATTTTTAAACGGAACCTCAGAAGAGTATGCTGAGTATGTGAAACAATATAAA-GATGATCCTGAAATATTGGAAAATACTGAAAAAA---TCAAGCAATGTGTTGACAGCACATTGATGGAGGAAGACAAGGCACATGCAAATGGTTTCATTGAAAAAATAGAAGCCAGCCC-ACTATGT

>B6_a1p

ATGAAGCTCACTGGTGCTCTTGTGATCCTCGGGA-CTGTCCTGTTCTTCCTAACTTCAGGAGGAGATTGTGGCATTTGCCTAGCTTCTAAACATGATGTCCAACTATTTCTTTATGGGACCCCAAAAGAGTTTCTTGAGAATGTGGAAAAATACAAA-GATGACTCTGAAACACTGGAAAGTAAGGAAAAAC---TCAAAAAATGTATTGATAGCACATTGACAGAGGAAGACAAGGCACATGCAACTGATTTCGTCGAAAAAAATAGAAGCCACCCTGTCCTGC

>rn_a2

ATGAAGCTCGCTGGTGCTCTGGTGCTGCTCGGGG-CTGCCCTG---CTCCTGACTTCAGGGGGAGACTGTGGCATTTGCCCAGTTATAGAAAAGGATGTTGGCCTATTTCTGTTCGGGACCCCGGAAGAGTATGTTGACTATGTGGCAGAATACGAA-GATGACCCTGAAGTATTGGAAAGTACTGAAAGCA---TCAAGCAATGTGTCGATAGCACCTTGACAGACGAAGACAAGCAAAACGCAGCTGCTGTCATCGAAAAAGTAAAAGCCAACCC-CTTGTGT

>rn_a1

ATGAAGCTCGCTGGTGCTCTGGTGCTGCTCGGGG-CTGCCCTG---CTCCTCACTTCAGGGGGAGACTGTGGCATTTGCCCAGTTATAGAAAAGGATGTTGGCCTATTTCTGTTCGGGACCCCGGAAGAGTATGTTGACTATGTGGCAGAATACGAA-GATGACCCTGAAGTATTGGAAAGTACTGAAAGCA---TCAAGCAATGTGTCGATAGCACCTTGACAGACGAAGACAAGCAAAACGCAGCTGCTGTCATCGAAAAAGTAAAAGCCAACCC-CTTGTGT

>rn_a3

ATGAAGCTCGCTGGTGCTCTGGTGCTGCTCGGGG-CTGCCCTG---CTCCTGACTTCAGGGGGAGACTGTGGCCTTTGCCCAGCTATGCAAACGAAAGTTGACCTATTACTTCATGGGACCGTAGACGACTATGTAGCCTATGTGGAACAATACAAA-GATAACCCTGCCATACTGGCAAATGCTGAGAGCA---TCAAGCAATGTGTCGACAGCAAGTTGACAAAGGAAGACAAGGATCATGCAACCAGTTTGGTCGAAAAAATAAAAGCCAGCCC-GTTGTGT

>B6_bg27

ATGAAGGGGACACTTCTTCTGCTGGCCTTGCTGGTGACTGGAGAGCTGGGCTTCCAGACAACGGAAGCATGTGCTCCTTTTGTCGGAGCCTATGTTAAAATACTGGGTGGAAATAGGCTAGCTCTGAATGCCTACCTTTCGATGTTTCAAGCTACTGCAGCGGAAAGGGT---GGCCTTTGAAAAGA----TCCAGGATTGCTTCAATGAGGAACCATTAACCACCAAATTAAAGAGTCCCCAAATAATGATGTCTATACTCTTCAGCTCTGAATGCAAGGCATACTATCCCGAAGATTCTGTAAACAAAATGGCGGA-TATGTTTAAACTGGATTCAATTAAT

>B6_bg19

ATGAAGGGGACACTTCTTCTGCTGGGCTTGCTGATAACTGGAGAGCTGAGCTTCCAGACAACAGAAGCATGTGTTCCTTTCTTCGAAGGCTATGCAAGTGTTGTCTCAGGAAGTAGGGTGTGGTTGTATCATGAACTCCAATCATTCGATGCTACTGCAGAGGAAAAGGT---GGCCTTGGAAAAAA----TCCAGGGTTGCTACAGAGAGGAAAGATTAAGAAATATACTTCTTGAACCCAAAATTATGGAAGCCATGGTCGCCAGCCCAGAATGCCGGTCATACTATAGTCTTAACAATTTTAGATCCATTTTAGA-CTTTATTTCCAACCTATTAGGAGAA

>B6_bg18

ATGAAGGGGACACTTCTTCTGCTGGGCTTGCTGGTGACTGGAGAACTGAGCTTCCAGACATCAGAAGCATGTGTTTCTTTCTTTGAAGGCTATGCAAGTGTTCTCTCAGGAAGTAGGGTGTGGTTGTATCAAGAACTACAGGCATTCGATGCTACTGCAGAGGAAAAGGT---GGCCTTGGAAAAAA----TCCAGAGTTGCTACAGTGAGGAAAGAATAAGAAATATACTTCTGGAACCCAAAATTATGGAAGCCATGGTCGCCAGCCCAGAATGCCGGTCATACCATAGTTTTAACAATTTTAGATCCATTTTAGA-CTTTATTTCCAACCTATTAGGAGAA

>B6_bg33p

ATGAAAGGGACCCTTCT---GCTGGCCTTGCTGGTGACTGGAGAACTGAGCTTCCAGACAACAGAATCACTTGTTCCTTTTTTCAAAGTCTATGCAAGTGTTCTCTCTGGAAAAAGAT------TGTATCAAGAACTCCAGATATTCAATGCTACTGCAGAGGTAAAGGT---GGCCTTGGGAAAAAA--ATCTATGTCTGCTACAGAGAGGAAAAATTAAGAAACATATTACTAGAACCTCAAATTAAGGAAGCCATGGACGCCAGCCCAGAATCCCAGTCATACTATAGTCTTAACAATTTTAGATCCATTTTAGA-CTTTATTTCCAACCTATTAGGAGAA

>B6_bg15p

ATGAAAGGGACCCTTCT---GCTGGCCTTGCTGGTGACTGGAGAACTGAGCTTCCAGACAACAGAATCACTTGTTCCTTTTTTCAATGTCTATGCAAGTGTTCTCTCTGGAAAAAGAT------TGTATCAAGAACTCCAGACATTCAATGCTACTGCAGAGGTAAAGGT---GGCCTTGGAAAAAAA---TCTGTGGCTGCTAGA----GGAAAATTTAAGAAACATGTTACTGGAACCTCAAATTAAGGAAGCCATGATTGCCAGCCAAGAATCCCAGTCAAACTATGAAGTTGACAATATAAGATCCATATTAGA-CTATATTTCCAGGCTATTAGGAGAG

>B6_bg31p

ATGAAGGGGACACTTCTTCTGCTGGGCTTGCTGGTGACTGGATAGCTGAGCTTCCAGACAACAGAAGCATGTGTTCCTTACTTCAAAGGCTATGCAAGTGTTGTCTCTGGAAGTAGGGTGTGGATGTATCAAGAACTCCAGGCATTCAATGCTACTGCAGAGGAAAAGCT---GGCCTTGGAATAA-----TCCAGGGCTGCTATAGAGAGGAAAGATTAAAAAACAAACTTCTGGAACCCAAAATTATGAAAGCCATGAATGCCAGCCCAGAATGCCAGTCACACTATAGAAGGGGCACTGTAAAATCCTTTTTAGA-CTATTTTTTCAAGCTATTGGGACAA

>B6_bg14p

ATGAATGGGACACTTCTTCTGCTGGACTTTCTGGTGACTGGAGAACTGAACTTCCAGACAACAGAAGCATGTGTTCCTTTCTTCTAAGTCTATTCAAGTGTTGTCTCTGGAAGTAGGCTGTGGATGTATCAAGAACTCCAGGCATTCAATGCTACTGCAGAGGAAAAGGT---GGCCTTGGAAAAAA----TCCAGGGCTGCTATAGAGAGGAAAGTTTAAGAAACATACTTCTGAAGCACAAAATTATGGAAGCCATGGACGCCAGCCCAGAATGCCAGGCACACTATAGAAGGGACACCGTAAAATCCTTTTTAGA-CTATCTTTTCAAGCTATTAGGATAA

>B6_bg17p

ATGAAAGGGACCCTTCT---GCTGGCCTTGCTGGTGACTGGAGAACTGAGCTTCCAGACAACAGAATCACTTGTTCCTTTTTTCAATGTCTATGCAAGTGTTCTCTCTGGAAAAAGAT------TGTATCAAGAACTCCAGACATTCAATGCTACTGCAGAGGTAAAGGT---GGCCTTGGAAAAAAA---TCTGTGGCTGCTAGA----GGAAAATTTAAGAAACATGTTACTGGAACCTCAAATTAAGGAAGCCATGATTGCCAGCCAAGAATCCCAGTCAAACTATGAAGTTGACAATATAAGATCCATATTAGA-CTATATTTCCAGGCTATTAGGAGAG

>B6_bg32p

ATGAAGGGGACACTTCTTCTGCTGGGCTTGCTGGTGACTGGATAGCTGAGCTTCCAGACAACAGAAGCATGTGTTCCTTACTTCAAAGGCTATGCAAGTGTTGTCTCTGGAAGTAGGGTGTGGATGTATCAAGAACTCCAGGCATTCAATGCTACTGCAGAGGAAAAGCT---GGCCTTGGAATAA-----TCCAGGGCTGCTATAGAGAGGAAAGATTAAAAAACAAACTTCTGGAACCCAAAATTATGAAAGCCATGAATGCCAGCCCAGAATGCCAGTCACACTATAGAAGGGGCACTGTAAAATCCTTTTTAGA-CTATTTTTTCAAGCTATTGGGACAA

>B6_bg16p

ATGAATGGGACACTTCTTCTGCTGGACTTTCTGGTGACTGGAGAACTGAACTTCCAGACAACAGAAGCATGTGTTCCTTTCTTCTAAGTCTATTCAAGTGTTGTCTCTGGAAGTAGGCTGTGGATGTATCAAGAACTCCAGGCATTCAATGCTACTGCAGAGGAAAAGGT---GGCCTTGGAAAAAA----TCCAGGGCTGCTATAGAGAGGAAAGTTTAAGAAACATACTTCTGAAGCACAAAATTATGGAAGCCATGGACGCCAGCCCAGAATGCCAGGCACACTATAGAAGGGACACCGTAAAATCCTTTTTAGA-CTATCTTTTCAAGCTATTAGGATAA

>B6_bg13p

ATGAAAGGGACACTTCT---GCTGGCCTTGCTGGTGACTGGAGAACTGAGCTTCCAGACAACAGAAGCACGTGTTCCTTTTTTCAAAGGCTATGCAAGTGTTCTCTCTGGAAAAAGAT------TGTATCAAGAACTCCAGACATTCAATGCTACTGCAGAGGTAAAGGT---GGCCTTGGAAAAAA----TCTGTGGCTGCTACA--GAGGAAAAATTAAGAAACATATTTCTGGAACCCCAAATTAAGGAAGCCATGGTCACAAGCCCAGAATTCAGGGCATACTATGGTAGTGACAATATAAGATCCATTATAGA-TCTTTTTGAAAAATTATTAGAAGAA

>B6_bg1

ATGAAGGGAACAGTTCT---GCTGGCCTTGCTGGTGAGTAGAGAGCTGGGCTTCCTGACAAAGGAAACATGCTTACCTTTCCTCAGTGTCTATTTGGGTGTCGTCTCTGGATACAGGTTTTGGTTACGTAAGGAACTTGCTACATTCCATCCTACTGTTGAGGAAAAAAAAAAGGCTTTTGAAAAAA----TCCAGGACTGCTATGAGGAGGCAGGACTAAAAACCAAAACTCAAGACATGAAATTTATGGCAGCTATACTCTTCAGCCCAGAATGCAAGTCATATTATACCAAAGAAGTCTTGATAAATATTCTGGA-TAAATTTTCTAAGAAATGAACCCAT

>B6_bg26

ATGAAGGGGACACTTCTTCTGCTGGCCTTGCTGGTGATTGGAGAGCTGGGCTTCCAGACAACGGAAGCATGCCTTTCTTTTGCCAGAACTTACGGAGCAATACTTACTCTAAGGAGGACCTTCCTGCATGGTGACCTTTCACAGTTTTATGCTACTGTAGCTGAAAGGGT---GGCCTTTGAAAAAA----TCCAGGATTGCTTTCGTGAGGAAGGACAAAAAACCATAATTCTGAATCCCCAAATTATGTTATCTCTATACTTAAGCCCAGAATGCAAGAAATACTATGGCAATGACCTCTTAAAGAAAATTCAAGA-TTTTCTTAACCAGTCAAATATCCAT

>B6_bg12

ATGAAGGGGATACTTCTTCTGCTGGGCTTGCTGATAACTGGAGAGCTGAGCTTCCAGACAACAGAAGCATGTGTTCCTTTCTTCGAAGTCTATGCAAGTGTTCTCTCAGGAAGTAGGGTGTGGTTGTATCATGAACTCCAATCATTCGATGCTACTGCAGAGGAAAAGGT---GGCCTTGGAAAAAA----TCCAGGGTTGCTACAGAGAGGAAAGATTAAGAAATATACTTCTGGAACCCAAAATTATGGAAGCCATGGTCGCCAGCCCAGAATGCCGGTCATACTATAGTCTTGACAATTTTAGATCCATTTTAGA-CTTTATTTCCAACCTATTAGGAGAA

>B6_bg11

ATGAAGGGGACACTTCTTCTGCTGGGCTTGCTGGTGACTGGAGAACTGAGCTTCCAGACATCAGAAGCATGTGTTTCTTTCTTTGAAGGCTATGCAAGTGTTCTCTCAGGAAGTAGGGTGTGGTTGTATCAAGAACTACAGGCATTCGATGCTACTGCAGAGGAAAAGGT---GGCCTTGGAAAAAA----TCCAGGGTTGCTACAGTGAGGAAAGAATAAGAAATATACTTCTGGAACCCAAAATTATGGAAGCCATGGTCGCCAGCCCAGAATGCCGGTCATACCATAGTCTTAACAATTTTAGATCCATTTTAGA-ATTTATTTCCAACCTATTAGGAGAA

>B6_bg30p

ATGAAAGGGACCCTTCT---GCTGGCCTTGCTGGTGACTGGAGAACTGAGCTTCCAGACAACAGAATCACTTGTTCCTTTTTTCAAAGTCTATGCAAGTGTTCTCTCTGGAAAAAGAT------TGTATCAAGAACTCCAGATATTCAATGCTACTGCAGAGGTAAAGGT---GGCCTTGGGAAAAAAAAATCTATGTCTGCTACAGAGAGGAAAAATTAAGAAACATATTACTAGAACCTCAAATTAAGGAAGCCATGGACGCCAGCCCAGAATCCCAGTCATACTATAGTCTTAACAATTTTAGATCCATTTTAGA-CTTTATTTCCAACCTATTAGGAGAA

>B6_bg10p

ATGAAAGGGACCCTTCT---GCTGGCCTTGCTGGTGACTGGAGAACTGAGCTTCCAGACAACAGAATCACTTGTTCCTTTTTTCAATGTCTATGCAAGTGTTCTCTCTGGAAAAAGAT------TGTATCAAGAACTCCAGACATTCAATGCTACTGCAGAGGTAAAGGT---GGCCTTGGAAAAAAA---TCTGTGGCTGCTAGA----GGAAAATTTAAGAAACATATTACTGGAACCTCAAATTAAGGAAGCCATGATTGCCAGCCAAGAATCCCAGTCAAACTATGAAGTTGACAATATAAGATCCATATTAGA-CTATATTTCCAGGCTATTAGGAGAG

>B6_bg29p

ATGAAGGGGACACTTCTTCTGCTGGGCTTGCTGGTGACTGGATAGCTGAGCTTCCAGACAACAGAAGAATGTGTTCCTTACTTCAAAGGCTATGCAAGTGTTGTCTCTGGAAGTAGGGTGTGGATGTATCAAGAACTCCAGGCATTCAATGCTACTGCAGAGGAAAAGGT---GGCCTTGGAATAA-----TCCAGGGCTGCTATAGAGAGGAAAGATTAAAAAACAAACTTCTGGAACCCAAAATTGTGAAAGCCATGAATGCCAGCCCAGAATGCCAGTCACACTATAGAAGGGGCACTGTAAAATCCTTTTTAGA-CTATTTTTTCAAGCTATTGGGACAA

>B6_bg9p

ATGAATGGGACACTTCTTCTGCTGGACTTTCTGGTGACTGGAGAACTGAACTTCCAGACAACAGAAGCATGTGTTCCTTTCTTCTAAGTCTATTCAAGTGTTGTCTCTGGAAGTAGGCTGTGGATGTATCAAAAATTCCAGGCATTCAATGCTACTGCAGAGGAAAAGGT---GGCCTTGGAAAAAA----TCCAGGGCTGCTATAGAGAGGAAAGATTAAGAAACATACTTCTGAAGCACAAAATTATGGAAGCCATGGACGCCAGCCCAGAATGCCAGGCACACTATAGAAGGGACACCGTAAAATCCTTTTTAGA-CTATCTTTTCAAGCTATTAGGATAA

>B6_bg8p

ATGAAAGGGACACTTCT---GCTGGCCTTGCTGGTGACTGGAGAACTGAGCTTCCAGACAACAGAAGCACGTGTTCCTTTTTTCAAAGGCTATGCAAGTGTTCTCTCTGGAAAAAGAT------TGTATCAAGAACTCCAGACATTCAATGCTACTGCAGAGGTAAAGGT---GGCCTTGGAAAAAA----TCTGTGGCTGCTACA--GAGGAAAAATTAAGAAACGTATTTCTGGAACCCCAAATTAAGGAAGCCATGGTCACAAGCCCAGAATTCAGGGCATACTATGGTAGTGACAATATAAGATCCATTATAGA-TCTTTTTGAAAAATTATTAGAAGAA

>B6_bg7

ATGAAGGGGACACTTCTTCTGCTGGGCTTGCTGGTGACTGGAGAACTGAGCTTCCAGACAACAGAAGCATGTTTTCCTTTCTTTGAAGCCTATGCAAGTGTTCTCTCAGGAAGTAGGGTGTGGTTGTATCAAGAACTCCAGGCATTCGATGCTACTGCAGAGGAAAAGGT---GGCCTTGGAAAAAA----TCCAGGACTGCTACAGTGAGGAAAGTATAAGAAATATACTTCTGGAACCCAAAATTATGGAAGCCATGGTTGCCAGCCCTGAATGCCTGTCATACTATGGTCTTGACAATATAAGATCCATTTTAGA-CTATATTTCCAAGCTATTAGGAGAA

>B6_bg6p

ATGAAGGGGACACTTCTTCTGCTGACCTTGCTGGTGACTGGAGAACTGACCTTCCATACAACAGAAGCACGTGTTCCTTTTTTCAAAGGCTATGCAAGTGTTCTCTCTGGGAAAAGAT------TGTATCAAGAACTCCAGACATTCAATGCTACTGCAGAGGTAAAGGT---GGCCTTAGAAAAAA----TCCAGGGCTGCTACAGAGAGGAAAGATTAAGAAACATATTTCTGGAACCC-ATATTAAGGAAGCCATGGTTTCCAGCCAAGAATCCCAGTCAAACTATGAAGTTGACAATATAAGTTCCATTTTAGA-CTATATTTCCAGGCTATTAGGAGAA

>B6_bg28p

ATGAAGGGGACACTTCTTCTGCTGGGCTTGCTGGTGACTGGATAGCTGAGCTTCCAGACAACAGAAGCATGTGTTCCTTTCTTCAAAGGCTATGCAAGTGTTGTCTCTGGAAGTAGGGTGTGGATGTATCAAGAACTCCAGGCATTCAATGCTACTGCAGAGGAAAAGGT---AGCCTTGGAAAAAA----TCCAGGGCTGCTATAGAGAGGAAAGATTAAGAAACAAACTTCTGGAACCCAAAATTATGGAAGCCATGAATGCCAGCCCAGAATGCCAGTCACACTATAAAGGGG-CACTGTAAAATCCTTTTTAGA-CTATTTTTTCAAGCTATTGGGACAA

>B6_bg2

ATGAAGGGGACACTTCTTCTGCTGGCCTTGCTGGTGACTGGAGAGCTGGGCTTCCAGAGAACGGAAGCATGCATACCTTTCTTCGGCGTATATTTGGGTATTCTCTCTGGAAACAGGATTGGGTTACATACAGAACTTGCTCCATTTGATCCTACTGTGGAGGAAAAGGA---GGCTTTTGAAAAAA----TCCAGGACTGCTATGAGGAGGAAGGACTAAAAGCTAAGACTGAAGATATGAAATTGATGACAACTATACTCTTCAGCTCAGAATGCAGGTCGTACTATACCAAAGAAGTCTTGAAGAACATTCTGGT-TAAGTTTTCCAAGAAATTAACCC-A

>B6_bg25p

ATGAAGAGGACACTTCTTCTGCTGGTCCTGCTGGTGACAGGAGAGCTGGGCTTCCAGAGAACAGAAGCTTGTCATCCTTTCTTCAGTATCTATTTTGCAGTACTCTCTGGACTTAAGATAATTATGTATAACAAACTTTTGCAGTTTGATGTTACTGCCATGGAATTGGA---GACCTTTGGAAAGC----TCCAGGAGTGCTACAATGAGGGAAGATTTGAAACTGAATTTCTGAATCCCTCAATTATGAAAGCCATAACTATCATCCCAGAATGCAGGGAATACTATACCAGTAAAGACATAAGAAAAATTG-GCT-TCTGTTTATCAAGACATGGATGCTT

>B6_bg5p

-------------------------------------------------------------CAGAAGCATATGTTCCTTTCTTTGAAGGCTATGCAAGTGTTGTCTCTGGAAGTAGGGTGTGGATGTATCAAGAACTCCAGGCATTCAATCCTGCTGCAGAGGAAAAGAT---GGCCTTGGAAAAAA----TCCAGGGCTGCTATAGAGAGGAAAGATTAAGAAACATACTTCTGGAACCCAAAAATATGGAAGCCATGGACGCCTGCCCAGAATGCCAGGCACACTATAGAAGGGACAACGTAAAATCCGTTTTAGA-CTATCTTTTCAAGGTATTTAGACAA

>B6_bg4p

ATGAAGGGGACACTTCTTCTGCTGGGCTTGCTGGTGACTGGAGAGCTGACCTTCTGGACAACAGAAGCATGTGTTCCTTTCTTTGAAGTCTATTCAAGTGTTGTCTCTGGAAGTAGGGTGTGGATGTATCAAGAACTCCAGGCATTCAATGCTACTGCAGAGGAAAAGGT---GGCCTTGGAAAAAA----TCCAGGGCTGCTATTGAAAGGAAAGATTAAGAAACATACTTCTGGAACGCAAAATTATGAAAGCCATGGACGCCAGCCCAGAATGCCAGTCACACTATAGAAGGGGCACTGTAAAATCCTATTTAGA-CTATCTTTTCAAGGTATTAAGACAG

>B6_bg3p

ATGAAGGGGACACTTCTTCTGCTGGCCTTCCTGGTGATTGGAGAGCTTGGCTTCCAGACAACAGAAGCATGTGCTTCTTTCTTCGGTGTCTATTTGAGCACTGTCTCTGGGAAAAGGCTTTGGTTGCATCATGAACTTTCTTATTTCAACCCTACTGATGGTGAAACAAA---GAGCTTCAAAAAAA----TCCAGGACTGCTATGAAGAGGCAGGACTAAAAGCTAAAAGTCAGGATGTCCAATTTATGGCAAGCATGTTCTTCAGCTCAGAATGCTTGAAATACTATAGCAATGACACTATGACAAAAATTTTAAG-TGTGATTACCAAGAAATGGATG---

>B6_bg24

ATGAAGGGGACACTTCTTCTGCTGGCCTTGCTGATGATTGGAGAGCTGGGCTTCCATACAACAGAAGCATGTGTTCCTTTCTTTGCTGGATATGCTGGTGTTATCTCAGGAAGCAGGCTGTGGTTGTATCATGAACTTAGCGCATTCAATGGTACACCTAAGGAAACAGT---GGCCTATGAAAAAA----TCCAGGACTGCTACAAAGAACAAGGAGTTAAATCCCAAACACTGGAACCACAAATTTTGGCCTCTATACTTGTCACCCCAGAATGCCTGCAGTACTATTCCGAAGAAACCTTTACGAAAATTAAGGA-TGCATTAAAAAAAATATCACAACAT

>B6_bg23p

ATGAAGGGGATACTTCT---GCTGTGCTTGCTGGTGACTGGAGAGCTGAGCTTCCAGACAACAGAAGTATGTGTTCTTTTCTTCAAATGCTATCCAAGTGCTCTCTCCGTAAAAAGAT------TGTATCAAGAACTCCAGGCATTCAATGCTACTGCAGGGGAAAAGGT---GGCCTTTGAAAAAA----TCCAGGACTGCTAGAAAGAGGGAGGATTAAGAAACATATTTATGGAACCTGAAATTATGGAAGCCATGGACTCCAGCCCTGAGTGCAAGGCATTCTATAATAGTGGCTCCATAAGGTCCATTTTAGA-CCTTCTTTCCAAGTTATTAGAGGAA

>B6_bg22p

ATGAAGGGGACACTTCTTGTGCTGGGCTTGCTGGTGACTGGAGAGCTGAGCTTCCACACAACAGAAGCATGTGTTCCTTACTTCAAAGGCTATACAAGTGTTGTCTCTGGATTAAGGTTTTTGATGCATCAAGAACTCCAGGCATTCAATGCTACTGCAGGAGAAAAGGT---GGCCTTTGAAAAAAA---TCCAGGACTGCTACAAAGAGGAAGGATTAAGAACCGTATTTCTGGAACCCAAAATTATGGAAGCCATGGTCTCCAGCCAAGAATGCCAGGCATTTTATACTACTGAAAACATATTGAACATTTTCGA-CCTTCTAGCTGGTTAATTAGAAGAA

>B6_bg21

ATGAAGGGAACACTTCTTCTGCTGGGCTTGCTGGTGACTGGAGAGCTGAGCTTCCAGACAACAGAAGCATGTGTTCCTTTCTTTGAAGGCTATGCAAGTGTTGTCTCTGGAAGTAGGGTGTGGATGTATCAAGAATTCCAGGCATTCGATGCTACTGCAGAGGAAAAGGT---GGCCTTGGAAAAAA----TCCAGGGCTGCTACAGAGAGGAGAGTTTAAGAAACATACTTCTGGAACCCAAAATTATGGAAGCTATGGTTGCCAGCCCAGAATGCCTGTCATACTATAGTAGTAACAATATAAGATGCATTTTAGA-CCTTCTTTCGAAGCTATTAGGACAA

>B6_bg34p

ATGAAGGA-ACACTTCTTCTGCTAGGCTTGCTGGTGACTGGAGAGCTGAGCTTCCAGACAACAGAAGCATATGTTCCTTTCTTCGAAGGCTATGTAAGTGTTGTCTTAGGAAGTAGGGTGTGGTTGTATCAAGAACTGTAGGCATCCGATGCTACTGCAGAGGAAAAGGT---GGCTTTGGAAAAAA----TCCAGGGCTGATACAGAGAGGAAAGATGAAGAAATATACTTCTGGAACACAAAATTATGGAAGCCATGGTTGCCAGCCCAGAATGGCGGTCATACTATGGTCTTGACAATGTAAGATCCATTTTAGA-CTATATTTTCAAGCTATTAGGAGAA

>B6_bg20

ATGAAGGGGACACTTCTTCTGCTGGGCTTGCTGGTGACTGGAGAACTGAGCTTCCAGACAACAGAAGCATGTCTTCCTTTCTTCGAAGGCTATGCAAGTGTTCTCTCAGGAAGTAGGGTGTGGTTGTATCAAGAACTCCAGGCATTCAATGCTACTGCAGAGGAAAAGGT---GGCCTTGGAAAAAA----TTCAGGACTGCTACAGTGAGGAAAGAATAAGAAATATACTTCTGGAACCCAAAATTATGGAAGCCATGGTTGCCAGCCCTGAATGCCTGTCATACTATGGTCTTGACAATATAAGATCCATTTTAGA-CTATATTTCCAAGCTATTAGGAGAA

>rn_bg1

ATGAAGGGGACACTTCTTCTGCTGACCTTGCTGGTGATTGGAGAGCTGGGCTTCCAGACAACGGAAGCATGCTTTCCTTTCTTTGGTCTCTATTTGGGCACTGTCTCTGGATCCAAGCTGTGGTTGCAGCATGAACTTTCCTACTTCAATCCTACTCCCGGGGAGACTGA---CGCCTATGAAAAAA----TCCAGAACTGCTTCAATGAGGCAGGATCACTTGGCAAATTTCGGGATATCAAAGTCATGGCAACCTTGCTCTTCAGCTCAAAATGCAAGACATACTATAGCAAGGAAGTGCTAACGAAAATTAAGGCTCAATTTACCCAAGCGTTGAAAGCAT

>rn_bg2

ATGAAGGGAACACTTCTTCTGCTGTCTTTGCTGGTGATTGGAGAGCTGGGCTTCCAGACAACGGAAGCGTGCCTTACTTTCTTCGAAGGCTATTGGAGAGTAGCCTTTGCAGGAAAGACACTGCTGAATTCCTTCCTTTCAAAGTTAGACGCTACAGCCGCAGAAAGGGT---GGCCCTTGAAAAAAA---TCCAAGACTGCTACCATGAAGGAGGACTAAAAACCAAACTTCTGGATCTCCAAGTTATGGAAGCCGTGATCACCAGCCAAGAATGCCTGACATACCATGGTGAAGAACTTGTAGCAAAAATTACGGATATATTTAC-CAGGTTAAACAGGC-T

>rn_bg3

ATGAAGGGGACACTTCTTCTGCTGACCTTGCTGGTGATTGGAGAGCTGGGTTTCCAGACAACAGAAGCATGCCTTCCTTTCTTCACTGCCTATGGGGGTGTTCTCTCGGGGAGCAAGCTGTGGTTGAACCATGAGCTTGATGCATACAATGCTACTACCGGGGAAAGGGA---GGCCTATGAAAAAA----TCCAGCAATGCTTCCGTGAAGGAGGACTAAAAGCCAAATTTCTGGAACCCAAAATTTTGAAAGCCATGGTCATCAGCCCAGAATGCCTGTCATACTATGGTAATGAAATTGTAGCCGAAATTACAGCTCTGTTAAG-CAAGTTACAGTTGCGT
