# Supplementary material for: Rapid bursts of androgen-binding protein (Abp) gene duplication occurred independently in diverse mammals
Source: BMC Evol Biol. 2008 Feb 12;8:46. doi: 10.1186/1471-2148-8-46 (PMC2291036; doi:10.1186/1471-2148-8-46)
Supplement: Additional file 2 — Names, strands, and genomic locations of Abp genes. Table showing Abp genes, locations, and genomic locations. Old and new names are listed and cross-referenced. [file 1471-2148-8-46-S2.doc]

| Species | *Name* | *Type* | *Gene* | *Alias* | *Location* | | | | *Comments* | | |
| --- | --- | --- | --- | --- | --- | --- | --- | --- | --- | --- | --- |
| Mouse | B6_a1ψ | alpha | ψG | ABPa1 | chr7 | - | 30,963,851 | 30,965,037 | Intron2:GT-to-AT; Exon3:ins(1bp) | |  |
|  | **B6_bg1** | beta/gamma | G | ABPbg1 | chr7 | + | 30,972,516 | 30,974,517 | Exon3:non-canonical STOP | |  |
|  | **B6_a2** | alpha | G | *ABPa2* | chr7 | - | 30,999,402 | 31,000,560 |  | |  |
|  | B6_bg2 | beta/gamma | G | *ABPbg2* | chr7 | + | 31,011,541 | 31,013,574 | Exon3:del(1bp), non-canonical STOP | |  |
|  | B6_bg3ψ | beta/gamma | ψG | *ABPbg12* | chr7 | - | 31,067,800 | 31,070,832 | Intron1:AG-to-AA | |  |
|  | **B6_a3** | alpha | G |  | chr7 | + | 31,084,367 | 31,085,552 |  | |  |
|  | B6_bg4ψ | beta/gamma | ψG | *ABPbg3* | chr7 | - | 31,109,793 | 31,111,938 | Exon2:STOP | |  |
|  | B6_a4ψ | alpha | ψG | *ABPa3* | chr7 | + | 31,118,434 | 31,119,616 | Exon1:del(2bp);Intron2:GT-to-CT; Exon3:ins(1bp) | |  |
|  | B6_a28ψ | alpha | ψG | *ABPa12* | chr7 | + | 31,152,582 | 31,153,757 | Intron1:AG-to-GG | |  |
|  | B6_bg5ψ | beta/gamma | ψG | *ABPbg4* | chr7 | - | 31,208,943 | 31,211,476 | Exon1: missing | |  |
|  | B6_a5ψ | alpha | ψG | *ABPa4* | chr7 | + | 31,216,200 | 31,216,572 | Intron2:GT-to-AT | |  |
|  | B6_b28ψ | beta/gamma | ψG |  | chr7 | - | 31,253,803 | 31,255,569 | Exon3:del(1bp) | |  |
|  | B6_bg6ψ | beta/gamma | ψG |  | chr7 | - | 31,326,500 | 31,328,286 | Intron1:AG-to-AT; Exon2:del(1bp);Intron2:GT-to-GC | |  |
|  | B6_a6ψ | alpha | ψG |  | chr7 | + | 31,334,359 | 31,335,549 | Exon1:ins(1bp); Exon2:ins(1bp) | |  |
|  | **B6_bg7** | beta/gamma | G |  | chr7 | - | 31,412,703 | 31,414,498 |  | |  |
|  | **B6_a7** | alpha | G |  | chr7 | + | 31,421,441 | 31,422,615 |  | |  |
|  | B6_bg8ψ | beta/gamma | ψG |  | chr7 | - | 31,487,540 | 31,489,318 | Exon2:del(2bp); Intron2:GT-to-GC | |  |
|  | B6_a8ψ | alpha | ψG |  | chr7 | + | 31,497,038 | 31,498,217 | Exon2:ins(1bp) | |  |
|  | B6_bg9ψ | beta/gamma | ψG |  | chr7 | - | 31,623,997 | 31,626,145 | Exon2:STOP | |  |
|  | B6_a9ψ | alpha | ψG |  | chr7 | + | 31,633,102 | 31,634,286 | Intron2:GT-to-CT; Stop:TGA-to-CGA | |  |
|  | B6_bg29ψ | beta/gamma | ψG |  | chr7 | - | 31,721,793 | 31,723,559 | Exon2:del(1bp) | |  |
|  | B6_bg10ψ | beta/gamma | ψG |  | chr7 | - | 31,800,907 | 31,802,699 | Exon2:ins(1bp); Exon2:del(4bp);Intron2:GT-to-GC | |  |
|  | **B6_a10** | alpha | G |  | chr7 | + | 31,809,628 | 31,810,807 |  | |  |
|  | BG_bg30ψ | beta/gamma | ψG |  | chr7 | - | 31,855,002 | 31,856,792 | Exon2:ins(1bp); Intron2:GT-to-GC | |  |
|  | **B6_bg11** | beta/gamma | G |  | chr7 | - | 31,918,097 | 31,919,883 |  | |  |
|  | **B6_a11** | alpha | G |  | chr7 | + | 31,931,616 | 31,932,794 |  | |  |
|  | **B6_bg12** | beta/gamma | G |  | chr7 | - | 32,034,204 | 32,035,989 |  | |  |
|  | **B6_a12** | alpha | G |  | chr7 | + | 32,042,957 | 32,044,139 |  | |  |
|  | **B6_a29** | alpha | G | *ABPa13* | chr7 | + | 32,150,300 | 32,151,479 |  | |  |
|  | B6_bg13ψ | beta/gamma | ψG |  | chr7 | - | 32,216,263 | 32,218,040 | Exon2:del(2bp); Intron2:GT-to-GC | |  |
|  | B6_a13ψ | alpha | ψG |  | chr7 | + | 32,225,770 | 32,226,949 | Exon2:ins(1bp) | |  |
|  | B6_bg14ψ | beta/gamma | ψG |  | chr7 | - | 32,356,412 | 32,358,561 | Exon2:STOP | |  |
|  | B6_a14ψ | alpha | ψG |  | chr7 | + | 32,365,524 | 32,366,709 | Intron2:GT-to-CT; Stop: TGA-to-CGA | |  |
|  | BG_bg31ψ | beta/gamma | ψG |  | chr7 | - | 32,454,056 | 32,455,822 | Exon2:del(1bp) | |  |
|  | B6_bg15ψ | beta/gamma | ψG |  | chr7 | - | 32,536,608 | 32,538,400 | Exon2:ins(1bp); Exon2:del(4bp); Intron2:GT-to-GC | |  |
|  | **B6_a15** | alpha | G |  | chr7 | + | 32,545,329 | 32,546,508 |  | |  |
|  | B6_bg16ψ | beta/gamma | ψG |  | chr7 | - | 32,583,302 | 32,585,451 | Exon2:STOP | |  |
|  | B6_a16ψ | alpha | ψG |  | chr7 | + | 32,592,414 | 32,593,599 | Intron2:GT-to-CT; Stop: TGA-to-CGA | |  |
|  | BG_bg32ψ | beta/gamma | ψG |  | chr7 | - | 32,680,955 | 32,682,721 | Exon2:del(1bp) | |  |
|  | B6_bg17ψ | beta/gamma | ψG |  | chr7 | - | 32,763,507 | 32,765,299 | Exon2:ins(1bp); Exon2:del(4bp);Intron2:GT-to-GC | |  |
|  | **B6_a17** | alpha | G |  | chr7 | + | 32,772,224 | 32,773,403 |  | |  |
|  | BG_bg33ψ | beta/gamma | ψG |  | chr7 | - | 32,820,431 | 32,822,218 | Exon2:del(1bp); Intron2:GT-to-GC | |  |
|  | **B6_bg18** | beta/gamma | G |  | chr7 | - | 32,880,816 | 32,882,602 |  | |  |
|  | **B6_a18** | alpha | G |  | chr7 | + | 32,894,327 | 32,895,505 |  | |  |
|  | **B6_bg19** | beta/gamma | G |  | chr7 | - | 32,987,294 | 32,989,079 |  | |  |
|  | **B6_a19** | alpha | G |  | chr7 | + | 32,996,066 | 32,997,248 |  | |  |
|  | **B6_bg20** | beta/gamma | G |  | chr7 | - | 33,073,267 | 33,075,060 |  | |  |
|  | **B6_a20** | alpha | G |  | chr7 | + | 33,082,009 | 33,083,193 |  | |  |
|  | BG_bg34ψ | beta/gamma | ψG | *ABPbg13* | chr7 | - | 33,151,786 | 33,153,577 | Exon1:del(1bp) | |  |
|  | **B6_bg21** | beta/gamma | G | *ABPbg5* | chr7 | - | 33,227,406 | 33,229,200 |  | |  |
|  | B6_a21ψ | alpha | ψG | *ABPa5* | chr7 | + | 33,236,135 | 33,237,318 | Exon3:del(1bp) | |  |
|  | B6_bg22ψ | beta/gamma | ψG | *ABPbg6* | chr7 | - | 33,313,041 | 33,314,812 | Exon2:ins(1bp) | |  |
|  | B6_a22ψ | alpha | ψG | *ABPa6* | chr7 | + | 33,323,381 | 33,324,564 | Exon1:del(1bp); Intron2:AG-to-AA | |  |
|  | B6_bg23ψ | beta/gamma | ψG | *ABPbg7* | chr7 | - | 33,334,073 | 33,335,861 | Exon2:STOP | |  |
|  | B6_a23ψ | alpha | ψG | *ABPa7* | chr7 | + | 33,343,921 | 33,345,092 | Intron1:AG-to-AT; Exon2:ins(1bp);Exon2:ins(2bp); Intron2:GT-to-AT | |  |
|  | **B6_bg24** | beta/gamma | G | *ABPbg8* | chr7 | - | 33,446,109 | 33,448,045 |  | |  |
|  | **B6_a24** | alpha | G | *ABPa8* | chr7 | + | 33,452,558 | 33,453,739 |  | |  |
|  | B6_bg25ψ | beta/gamma | ψG | *ABPbg9* | chr7 | - | 33,561,794 | 33,563,578 | Exon3:ins(1bp); Exon3:del(1bp) | |  |
|  | B6_a25ψ | alpha | ψG | *ABPa9* | chr7 | + | 33,576,700 | 33,577,844 | Init_Met:ATG-to-TTA; Exon2:del(8bp);Exon2:del(1bp); Exon2:del(1bp);Intron2:GT-to-AT | |  |
|  | **B6_bg26** | beta/gamma | G | *ABPbg10 = gamma* | chr7 | - | 33,651,928 | 33,653,724 |  | |  |
|  | B6_a26ψ | alpha | ψG | *ABPa10* | chr7 | + | 33,667,564 | 33,668,743 | Intron1:AG-to-AT; Intron2:GT-to-AT | |  |
|  | **B6_bg27** | beta/gamma | G | *ABPbg11 = beta* | chr7 | - | 33,720,846 | 33,722,626 |  | |  |
|  | **B6_a27** | alpha | G | *ABPa11 = alpha* | chr7 | + | 33,730,327 | 33,731,509 |  | |  |
|  | B6_a30ψ | alpha | ψG | *ABPa14 (33804274-33809465)* | chr7 | + | 33,804,273 | 33,809,465 | Exon1:missing | |  |
|  |  |  |  |  |  |  |  |  |  | |  |
| Rat | a1 | alpha | G |  | chr1 | - | 86,328,781 | 86,329,929 |  | |  |
|  | bg1 | beta/gamma | G |  | chr1 | + | 86,335,465 | 86,337,420 |  | |  |
|  | bg2 | beta/gamma | G |  | chr1 | - | 86,393,329 | 86,394,239 |  | |  |
|  | a2 | alpha | G |  | chr1 | + | 86,401,016 | 86,402,164 |  | |  |
|  | bg3 | beta/gamma | ψG |  | chr1 | - | 86,536,945 | 86,538,717 |  | |  |
|  | a3 | alpha | G |  | chr1 | + | 86,546,704 | 86,547,845 |  | |  |
|  |  |  |  |  |  |  |  |  |  | |  |
| Bushbaby | 1 | beta/gamma | ψG |  | contig_392979 | + | 917 | 1,605 | ³ | |  |
|  |  |  |  |  |  |  |  |  |  | |  |
|  |  |  |  |  |  |  |  |  |  | |  |
| Cat | 1 | beta/gamma | G |  | contig_678245 | - | 849 | 1,604 |  | |  |
|  | 1 | alpha | G |  | contig_678245 | + | 5,589 | 5,994 |  | |  |
|  |  |  |  |  |  |  |  |  |  | |  |
| Cattle | 1 | beta/gamma | ψG |  | chr18 | - | 40,145,149 | 40,145,892 | ³ | |  |
|  | 2 | beta/gamma | ψG |  | scaffold1810 | - | 12,790 | 14,859 |  | |  |
|  | 3 | alpha | ψG |  | scaffold1810 | + | 20,682 | 22,039 |  | |  |
|  | 4 | beta/gamma | ψG |  | scaffold1810 | - | 72,114 | 74,143 |  | |  |
|  | 5 | beta/gamma | ψG |  | scaffold1810 | - | 84,776 | 85,550 | ³ | |  |
|  | 6 | beta/gamma | G |  | scaffold1810 | - | 115,335 | 117,385 |  | |  |
|  | 7 | alpha | ψG |  | scaffold1810 | + | 121,868 | 123,257 |  | |  |
|  | 8 | beta/gamma | ψG |  | scaffold2740 | + | 13,416 | 16,115 | insert before second exon | |  |
|  | 9 | alpha | G |  | scaffold2740 | - | 41,729 | 43,109 |  | |  |
|  | 10 | beta/gamma | G |  | scaffold2740 | + | 55,274 | 57,322 |  | |  |
|  | 11 | alpha | G |  | scaffold2740 | - | 80,322 | 81,706 |  | |  |
|  | 12 | beta/gamma | G |  | scaffold2740 | + | 89,716 | 91,772 |  | |  |
|  | 13 | alpha | G |  | scaffold5723 | - | 17,208 | 18,582 |  | |  |
|  | 14 | beta/gamma | ψG |  | scaffold5723 | + | 28,905 | 30,967 |  | |  |
|  | 15 | beta/gamma | ψG |  | scaffold5723 | + | 38,084 | 40,190 |  | |  |
|  | 16 | beta/gamma | G |  | scaffold7757 | - | 7,905 | 9,923 |  | |  |
|  | 17 | beta/gamma | G |  | scaffold8101 | - | 20,737 | 23,255 |  | |  |
|  | 18 | alpha | ψG |  | scaffold8101 | + | 29,050 | 30,413 |  | |  |
|  |  |  |  |  |  |  |  |  |  | |  |
| Dog | 1 | beta/gamma | G |  | chr1 | - | 120,703,846 | 120,706,093 |  | |  |
|  | 2 | alpha | G |  | chr1 | + | 120,709,581 | 120,710,859 |  | |  |
|  |  |  |  |  |  |  |  |  |  | |  |
| Guinea pig | 1 | beta/gamma | G |  | contig_334648 | - | 1,030 | 1,784 | ³ | |  |
|  |  |  |  |  |  |  |  |  |  | |  |
| Horse | 1 | alpha | G |  | scaffold_71.5000001-9474437 | - | 610,438 | 611,887 |  | |  |
|  | 2 | beta/gamma | G |  | scaffold_71.5000001-9474437 | + | 616,040 | 616,807 |  | |  |
|  | 3 | alpha | ψG |  | scaffold_71.5000001-9474437 | + | 928,533 | 933,256 |  | |  |
|  | 4 | alpha | ψG |  | scaffold_71.5000001-9474437 | + | 971,957 | 973,268 |  | |  |
|  |  |  |  |  |  |  |  |  |  | |  |
| Little brown bat | 1 | beta/gamma | G |  | contig_298238 | - | 1,007 | 3,167 |  | |  |
|  | 2 | alpha | G |  | contig_298238 | + | 6,596 | 7,918 |  | |  |
|  |  |  |  |  |  |  |  |  |  | |  |
| Opossum | 1 | alpha | G |  | chr1 | - | 425,284,993 | 425,287,729 |  | |  |
|  | 2 | alpha | G |  | chr1 | - | 425,359,186 | 425,362,870 |  | |  |
|  | 3 | beta/gamma | G |  | chr1 | + | 425,395,433 | 425,395,612 | only second exon | |  |
|  | 4 | beta/gamma | G |  | chr1 | + | 425,401,716 | 425,401,760 | only first exon | |  |
|  | 5 | beta/gamma | G |  | chr1 | + | 425,417,288 | 425,417,446 | only second exon | |  |
|  | 6 | alpha | ψG |  | chr1 | + | 425,454,167 | 425,458,814 | first and second exon fused | |  |
|  |  |  |  |  |  |  |  |  |  | |  |
| Rabbit | 1 | alpha | ψG |  | super_110373 | - | 5,432 | 6,317 |  | |  |
|  | 2 | beta/gamma | G |  | super_111552 | - | 547 | 1,565 | ³ | |  |
|  | 3 | alpha | G |  | super_111552 | + | 5,731 | 6,535 | second exon truncated | |  |
|  | 4 | alpha | ψG |  | super_116683 | - | 3,748 | 5,075 |  | |  |
|  | 5 | alpha | ψG |  | super_118378 | + | 1,454 | 2,781 |  | |  |
|  | 6 | beta/gamma | G |  | super_125878 | + | 10,170 | 12,025 |  | |  |
|  | 7 | alpha | ψG |  | super_129236 | - | 3,670 | 4,996 |  | |  |
|  | 8 | alpha | ψG |  | super_130945 | + | 1,753 | 5,164 |  | |  |
|  | 9 | alpha | ψG |  | super_133954 | - | 879 | 4,401 |  | |  |
|  | 10 | beta/gamma | G |  | super_134549 | - | 585 | 1,601 | ³ | |  |
|  | 11 | alpha | G |  | super_134549 | + | 5,719 | 6,522 |  | |  |
|  | 12 | alpha | ψG |  | super_13665 | - | 518 | 1,229 |  | |  |
|  | 13 | alpha | ψG |  | super_137086 | + | 1,009 | 4,376 | insert at first intron. | |  |
|  | 14 | alpha | ψG |  | super_138316 | - | 5,201 | 13,188 |  | |  |
|  | 15 | alpha | G |  | super_142518 | + | 1,564 | 2,363 |  | |  |
|  | 16 | alpha | ψG |  | super_148034 | - | 10,542 | 11,862 |  | |  |
|  | 17 | alpha | ψG |  | super_149645 | + | 9,253 | 12,690 |  | |  |
|  | 18 | alpha | ψG |  | super_150613 | - | 1,954 | 5,308 | mispredicted first exon | |  |
|  | 19 | alpha | ψG |  | super_153398 | + | 1,269 | 2,589 |  | |  |
|  | 20 | alpha | ψG |  | super_163925 | + | 10,127 | 13,492 |  | |  |
|  | 21 | alpha | ψG |  | super_164348 | + | 8,518 | 9,847 |  | |  |
|  | 22 | alpha | ψG |  | super_168942 | + | 1,713 | 3,591 | deletion in secon exon | |  |
|  | 23 | alpha | ψG |  | super_173890 | - | 16,365 | 17,688 |  | |  |
|  | 24 | alpha | ψG |  | super_175060 | + | 44,639 | 45,359 |  | |  |
|  | 25 | alpha | G |  | super_176488 | - | 2,050 | 3,368 |  | |  |
|  | 26 | beta/gamma | G |  | super_176488 | - | 14,781 | 16,636 |  | |  |
|  | 27 | alpha | ψG |  | super_187678 | + | 4,560 | 5,433 |  | |  |
|  | 28 | alpha | ψG |  | super_200987 | - | 57,371 | 58,696 |  | |  |
|  | 29 | alpha | ψG |  | super_203750 | - | 5 | 340 | ³, second exon truncated | |  |
|  | 30 | beta/gamma | G |  | super_207046 | - | 8,516 | 10,366 |  | |  |
|  | 31 | alpha | G |  | super_207046 | + | 14,465 | 15,270 |  | |  |
|  | 32 | alpha | ψG |  | super_25061 | - | 173 | 545 | ³ | |  |
|  | 33 | alpha | ψG |  | super_37356 | - | 994 | 4,355 |  | |  |
|  | 34 | alpha | G |  | super_64544 | + | 1,265 | 2,068 |  | |  |
|  | 35 | alpha | ψG |  | super_64951 | - | 2,453 | 3,784 |  | |  |
|  | 36 | alpha | ψG |  | super_65402 | - | 2,230 | 3,548 |  | |  |
|  | 37 | alpha | ψG |  | super_74358 | - | 284 | 663 | ³ | |  |
|  | 38 | alpha | G |  | super_80237 | - | 199 | 580 | ³ | |  |
|  | 39 | alpha | ψG |  | super_80321 | - | 719 | 4,085 |  | |  |
|  | 40 | alpha | ψG |  | super_82580 | - | 4,848 | 6,176 |  | |  |
|  | 41 | alpha | ψG |  | super_83292 | - | 4,916 | 6,242 |  | |  |
|  | 42 | alpha | ψG |  | super_84618 | - | 3,477 | 4,792 |  | |  |
|  | 43 | alpha | ψG |  | super_86679 | - | 3,842 | 5,158 |  | |  |
|  |  |  |  |  |  |  |  |  |  | |  |
| Squirrel | 1 | beta/gamma | ψG |  | contig_534814 | - | 1,854 | 3,960 | ³ | |  |
|  | 2 | alpha | G |  | contig_735823 | - | 51 | 464 |  | |  |
|  |  |  |  |  |  |  |  |  |  | |  |
| Tree shrew | 1 | beta/gamma | G |  | contig_19463 | - | 1,328 | 1,532 | ³ | |  |
|  | 2 | alpha | G |  | contig_822596 | + | 657 | 1275 | ³, first exon missing | |  |
| ³: third exon not predicted | |  |  |  |  |  |  |  |  |  | |
