# Supplementary material for: Rapid bursts of androgen-binding protein (Abp) gene duplication occurred independently in diverse mammals
Source: BMC Evol Biol. 2008 Feb 12;8:46. doi: 10.1186/1471-2148-8-46 (PMC2291036; doi:10.1186/1471-2148-8-46)
Supplement: Additional file 4 — PAML values for tests of positive selection on Abp genes. Table of PAML log likelihood ratio test statistics comparing models M7/M8 and M1/M2 for Abpa or Abpbg genes. [file 1471-2148-8-46-S4.doc]

|  |  |  |  | M1/M2 | | | | M7/M8 | | | |
| --- | --- | --- | --- | --- | --- | --- | --- | --- | --- | --- | --- |
|  |  | Parts | #sequences | Chi2 | df | P-value | Test | Chi2 | df | P-value | Test |
| Alphas | All | Genes | 35 | 2.89 | 2 | 2.36E-001 | failed | 147.76 | 2 | 8.20E-033 | passed |
|  | Mouse | Genes | 14 | 0.56 | 2 | 7.57E-001 | failed | 1.17 | 2 | 5.57E-001 | failed |
|  | All | Genes + Pseudogenes | 88 | 6.86 | 2 | 3.24E-002 | passed | 0 | 2 | 1.00E+000 | failed |
|  | Rabbit | Genes | 7 | 2.75 | 2 | 2.53E-001 | failed | 2.75 | 2 | 2.53E-001 | failed |
| Betas | Cow | Genes | 5 | 27.54 | 2 | 1.05E-006 | passed | 27.83 | 2 | 9.04E-007 | passed |
|  | All | Genes | 29 | 81.3 | 2 | 2.22E-018 | passed | 89.81 | 2 | 3.14E-020 | passed |
|  | Mouse | Genes | 10 | 16.86 | 2 | 2.18E-004 | passed | 19.8 | 2 | 5.01E-005 | passed |
|  | All | Genes + Pseudogenes | 64 | 184.18 | 2 | 1.01E-040 | passed | 188.83 | 2 | 9.92E-042 | passed |
|  | Rabbit | Genes | 5 | 10.53 | 2 | 5.17E-003 | passed | 10.53 | 2 | 5.17E-003 | passed |

Only sequences with at least five genes are shown, thus there is no entry for cow alphas.

#Sequences: sequences in multiple alignment

Chi2: chi squared value

P-Value: p-value of log likelihood ratio test

Df: degrees of freedom

Test: passed/failed at level P < 0.05
