# Supplementary figures and images for: Rapid bursts of androgen-binding protein (Abp) gene duplication occurred independently in diverse mammals
Source: BMC Evol Biol. 2008 Feb 12;8:46. doi: 10.1186/1471-2148-8-46 (PMC2291036; doi:10.1186/1471-2148-8-46)

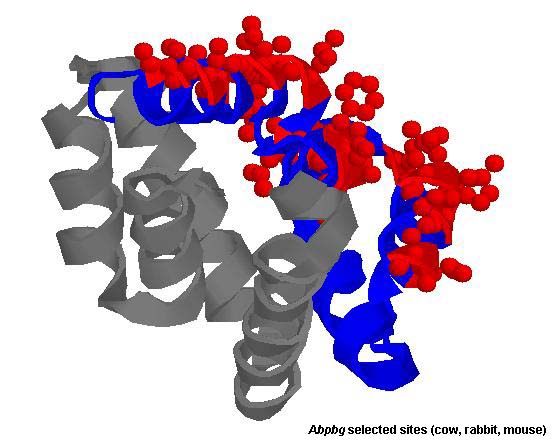

Supplement: Additional file 5 — Selected sites on Abpbg superimposed on ribbon structure of cat Fel dI. Beta subunit backbone is shown in blue with side chains of selected amino acids colored red. Analysis was performed with all cow, rabbit, and mouse paralogs. [file 1471-2148-8-46-S5.JPEG]

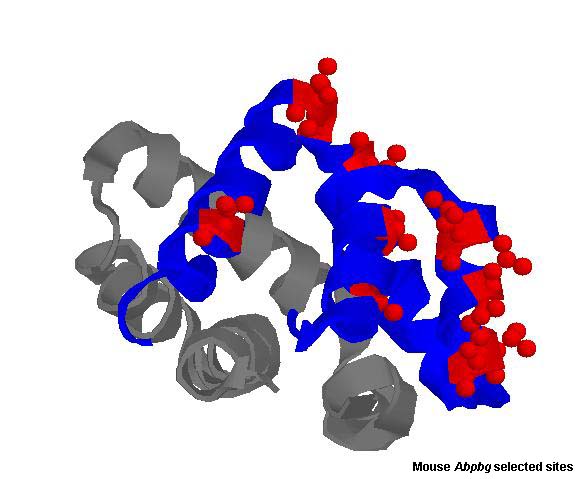

Supplement: Additional file 6 — Selected sites on Abpbg superimposed on ribbon structure of cat Fel dI. Beta subunit backbone is shown in blue with side chains of selected amino acids colored red. Analysis was performed with only mouse paralogs. [file 1471-2148-8-46-S6.JPEG]

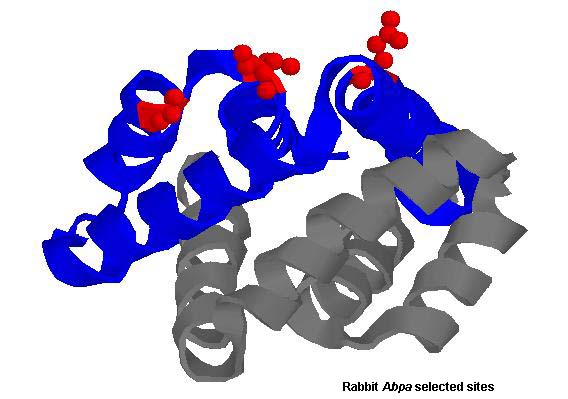

Supplement: Additional file 7 — Selected sites on Abpa superimposed on ribbon structure of cat Fel dI. Alpha subunit backbone is shown in blue with side chains of selected amino acids colored red. Analysis was performed with only rabbit paralogs. [file 1471-2148-8-46-S7.JPEG]

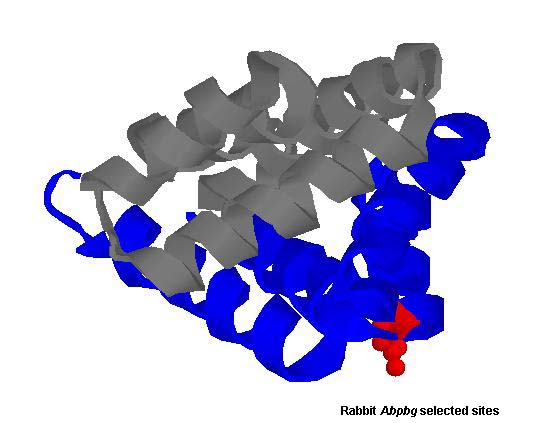

Supplement: Additional file 8 — Selected sites on Abpbg superimposed on ribbon structure of cat Fel dI. Beta subunit backbone is shown in blue with side chains of selected amino acids colored red. Analysis was performed with only rabbit paralogs. [file 1471-2148-8-46-S8.JPEG]

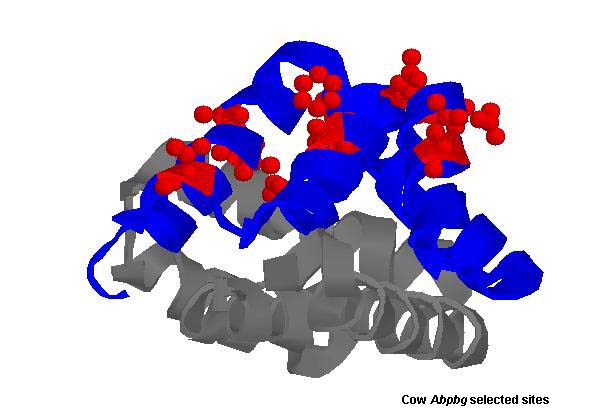

Supplement: Additional file 9 — Selected sites on Abpbg superimposed on ribbon structure of cat Fel dI. Beta subunit backbone is shown in blue with side chains of selected amino acids colored red. Analysis was performed with only cow paralogs. [file 1471-2148-8-46-S9.JPEG]

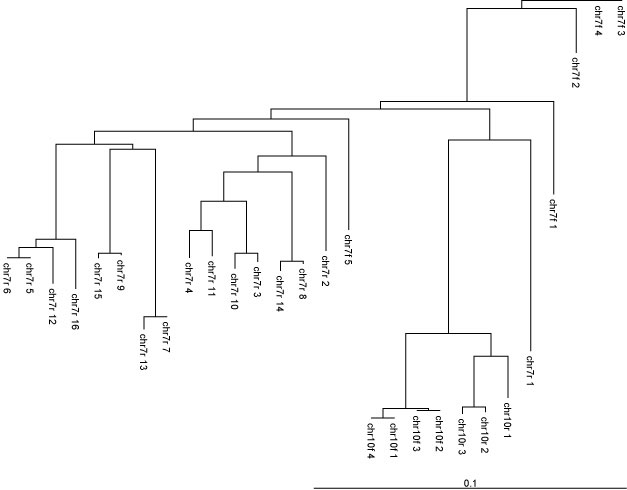

Supplement: Additional file 10 — L23a pseudogene tree. Neighbor-joining tree showing phylogenetic relationships of L23a pseudogenes found in the Abp-containing region of mouse chromosome 7. [file 1471-2148-8-46-S10.JPEG]
