# Supplementary material for: Rapid bursts of androgen-binding protein (Abp) gene duplication occurred independently in diverse mammals
Source: BMC Evol Biol. 2008 Feb 12;8:46. doi: 10.1186/1471-2148-8-46 (PMC2291036; doi:10.1186/1471-2148-8-46)
Supplement: Additional file 11 — Abp EST references for non-rodent taxa. References to ESTs corresponding to Abp genes in non-rodent taxa. [file 1471-2148-8-46-S11.doc]

**Alphas**

1: EE852773 wool follicle

021030OSPA1020063HT OSPA Ovis aries cDNA, mRNA sequence

gi|114742888|gb|EE852773.1|[114742888]

2: EE757651 Orf infected skin

020605OCS411018052HT OCS4 Ovis aries cDNA, mRNA sequence

gi|114444767|gb|EE757651.1|[114444767]

3: EE753243 Louse infected skin

020502OCSI1025057HT OCS1 Ovis aries cDNA, mRNA sequence

gi|114439887|gb|EE753243.1|[114439887]

4: EE849019 wool follicle

030729OSCA3019045HT OSCA Ovis aries cDNA, mRNA sequence

gi|114735628|gb|EE849019.1|[114735628]

5: CD052146 Cashmere goat skin

PLY385 Cashmere goat skin cDNA library Capra hircus cDNA 5-, mRNA sequence

gi|31088829|gb|CD052146.1|[31088829]

6: EE847635 wool follicle

030729OSCA3007044HT OSCA Ovis aries cDNA, mRNA sequence

gi|114734244|gb|EE847635.1|[114734244]

7: EE848854 wool follicle

030729OSCA3006094HT OSCA Ovis aries cDNA, mRNA sequence

gi|114735463|gb|EE848854.1|[114735463]

8: EE852548 wool follicle

021030OSPA1029022HT OSPA Ovis aries cDNA, mRNA sequence

gi|114742663|gb|EE852548.1|[114742663]

**Beta/Gamma**

1: CF768578

CES002347 Bos taurus **skin** cDNA library Bos taurus cDNA clone CCL002347 5-, mRNA sequence

gi|37717797|gb|CF768578.1|[37717797]

2: CF769035

CES000322 Bos taurus **skin** cDNA library Bos taurus cDNA clone CCL000322 5-, mRNA sequence

gi|37718254|gb|CF769035.1|[37718254]

3: DW521262

CES004391 Bos taurus **fat** cDNA library Bos taurus cDNA clone CCL004391, mRNA sequence

gi|84979369|gb|DW521262.1|[84979369]

4: CF763323

CES006167 Bos taurus **skin** cDNA library Bos taurus cDNA clone CCL006167 5-, mRNA sequence

gi|37712541|gb|CF763323.1|[37712541]

5: CD052155

PLY394 Cashmere goat **skin** cDNA library Capra hircus cDNA 5-, mRNA sequence

gi|31088838|gb|CD052155.1|[31088838]

6: CD052153

PLY392 Cashmere goat **skin** cDNA library Capra hircus cDNA 5-, mRNA sequence

gi|31088836|gb|CD052153.1|[31088836]

7: EE757505

020731OCS411054041HT OCS4 Ovis aries **skin** cDNA, mRNA sequence

gi|114444621|gb|EE757505.1|[114444621]

8: CF763196

CES006312 Bos taurus **skin** cDNA library Bos taurus cDNA clone CCL006312 5-, mRNA sequence

gi|37712414|gb|CF763196.1|[37712414]
